# Toward redesigning the PEG surface of nanocarriers for tumor targeting: impact of inner functionalities on size, charge, multivalent binding, and biodistribution†
†Electronic supplementary information (ESI) available: Experimental section, tables, synthetic schemes, NMR and mass spectra, SAXS profiles, cytotoxicity assay results, confocal fluorescence micrographs, and SPECT images and movies. See DOI: 10.1039/c6sc05640g



**DOI:** 10.1039/c6sc05640g

**Published:** 2017-04-20

**Authors:** Ju Young Heo, Se Hun Kang, Young-Hwa Kim, Suyeon You, Kyeong Sik Jin, Seung Won Kim, Hye-youn Jung, Kyung Oh Jung, Chul-Hee Lee, Mi Jung Kim, Soo-Eun Sung, Boram Kim, Insung S. Choi, Hyewon Youn, June-Key Chung, Seok-ki Kim, Yoonkyung Kim

**Affiliations:** a Korea Research Institute of Bioscience and Biotechnology , Daejeon , 34141 , Korea . Email: ykim@kribb.re.kr; b Department of Chemistry , Korea Advanced Institute of Science and Technology , Daejeon , 34141 , Korea; c Molecular Imaging and Therapy Branch , National Cancer Center , Goyang , 10408 , Korea . Email: skkim@ncc.re.kr; d Department of Biomedical Sciences , Seoul National University College of Medicine , Seoul , 03080 , Korea . Email: jkchung@snu.ac.kr; e Cancer Research Institute , Seoul National University College of Medicine , Seoul , 03080 , Korea; f Pohang Accelerator Laboratory , Pohang University of Science and Technology , Pohang , 37673 , Korea; g Department of Nuclear Medicine , Seoul National University Hospital , Seoul , 03080 , Korea; h Korea University of Science and Technology (UST) , Daejeon , 34113 , Korea

## Abstract

Achieving accurate and efficacious tumor targeting with minimal off-target effects is of paramount importance in designing diagnostic and therapeutic agents for cancer. In this respect, nanocarriers have gained enormous popularity because of their attainable multifunctional features, as well as tumor-targeting potential by extravasation. However, once administered into the bloodstream, nanocarriers face various *in vivo* obstacles that may significantly impair their performance needed for clinical translation. Herein, we demonstrate a strategy to enhance tumor-targeting efficiency by embedding functionalities in the interior region of partially PEGylated nanocarriers (*ca.* 10 nm in diameter), intended for active or passive targeting. The cooperative impact of these topologically inner functional groups (IFGs) was marked: enhancements of >100-fold in IC_50_ *in vitro* (*e.g.*, a high-avidity ligand with cationic IFGs) and >2-fold in tumor accumulation at 2 h post-injection *in vivo* (*e.g.*, a high-avidity ligand with anionic IFGs), both against the fully PEGylated counterpart. Analogous to allosteric modulators, properly employed IFGs may substantially improve the process of effectively directing nanocarriers to tumors, which is otherwise solely dependent on avidity or extravasation.

## Abstract 2

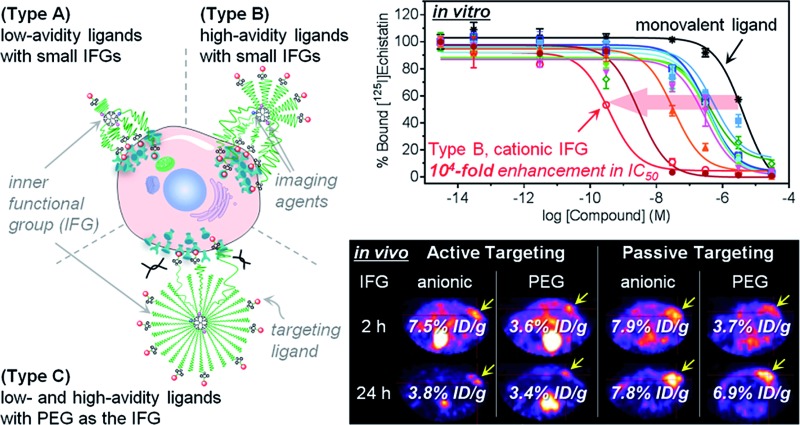
A simple strategy to enhance the tumor-targeting efficiency of PEGylated nanocarriers is demonstrated.

## Introduction

In cancer nanomedicine,^1–6^ most nano-sized agents are administered intravascularly, where active and passive targeting methods are currently the two most frequently used strategies for specific localization at the tumor site. Passive tumor targeting is fulfilled by the enhanced permeability and retention (EPR) effect,^7,8^ utilizing the leaky nature of the tumor vascular endothelium, through which the long-circulating nano-sized agents can extravasate and be increasingly deposited in the tumor interstitium over time. Here, the prolonged circulation of nano-sized agents in the vascular system is achieved almost indiscriminately by densely packing the surface of nanocarriers with long poly(ethylene glycol) (PEG)^9^ chains for a stealth effect. The tumor targeting by these EPR-based agents, however, is reported to be relatively inefficient for the early-stage primary and unvascularized metastatic cancers, and exhibited tumor-type dependency as well as intra- and inter-species heterogeneity.^6,10,11^ Active targeting is achieved by the tumor-specific binding of targeting ligands (*e.g.*, small molecules, peptides, and antibodies) usually attached to the surface of nanocarriers, and is generally reinforced by the multivalent effect (*i.e.*, avidity).^12–14^ Although some of the organic polymer-based nanoparticles and dendrimers have their ligands attached directly to the surface,^15^ most of the nanocarriers (*e.g.*, liposomes, micelles, and inorganic nanoparticles) have them attached through PEG groups as spacers (or linkers).^10,16,17^ Typically, the degree of surface derivatization with targeting ligands is driven maximally without any intended stoichiometric control, and thus depends on the efficiency of the conjugation chemistry and the properties (*e.g.*, steric and electronic effects) of neighboring groups. In fact, multivalent binding in tumor targeting is not exhaustive, but is limited by many factors:^4,5,12–14^ the size, shape, hardness, and surface properties of a nanocarrier, conformational flexibility and length of the spacer, inter-ligand distance on a nanocarrier, and inter-receptor distance and receptor density on a target cell. We therefore hypothesized that restricting the number of ligands on a nano-sized agent and covering the remainder of its surface into a functional group (other than PEG) that could cooperatively impact the ligand–receptor binding might consequently enhance the overall tumor-targeting efficiency.

**Fig. 1:**
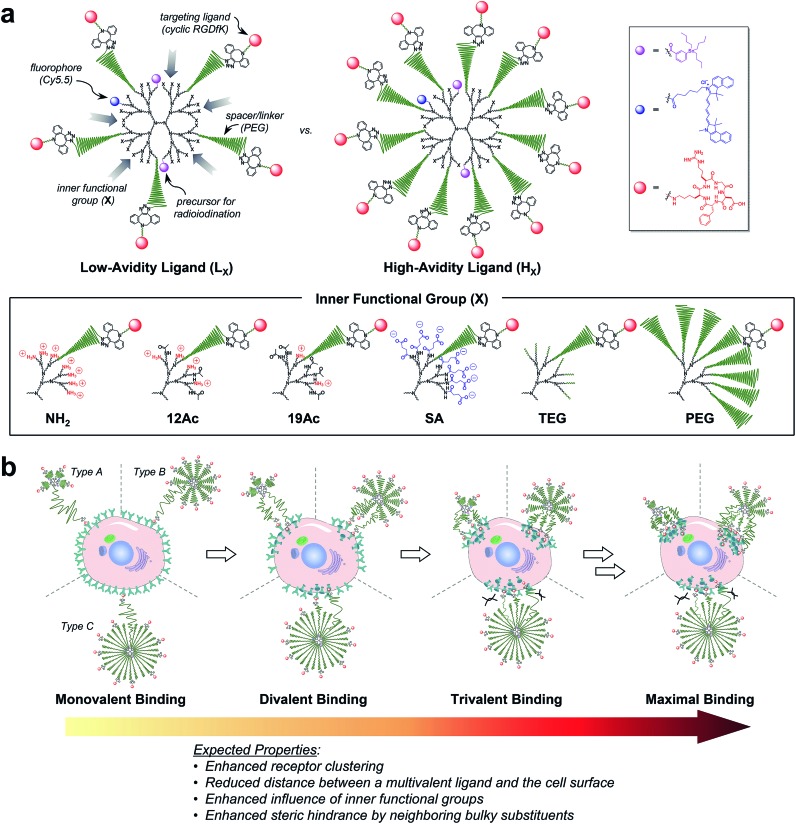
Nano-sized PEGylated dendritic multivalent ligands for tumor targeting. (a) Schematic of low-avidity ligands (**L_X_**; left), in which *ca.* 14% (4–5 out of 32) of the G3 polyamidoamine (PAMAM) dendrimer surface is substituted with α_V_β_3_ integrin-specific c(RGDfK) moieties (red circles) through long PEG spacers (green wavy lines), and high-avidity ligands (**H_X_**; right), in which *ca.* 32% (10–11 out of 32) of the surface is substituted with c(RGDfK) moieties in the same manner. In all these structures, TBSB (a precursor for *in situ* radioiodination; pink circles) and Cy5.5 (a fluorophore; blue circles) moieties for *in vivo* and *in vitro* tracking, respectively, were attached to the dendrimer surface, each in a small equimolar portion. Additionally, the residual surface amino groups of the PAMAM dendrimers were converted into different types of inner functional group (IFG; **X**). The untargeted agents (**PL_X_** and **PH_X_**; not shown) used in this study are the synthetic precursors of the respective targeted agents lacking terminal c(RGDfK)–DBCO moieties (see Fig. S1†). (b) Proposed profiles of the binding of our three different types of multivalent ligand (by PEG density) to α_V_β_3_ integrin receptors expressed on a target tumor cell: low-avidity ligands with small IFGs adopting a collapsed mushroom-like PEG conformation (type A); high-avidity ligands with small IFGs adopting a brush-like PEG conformation (type B); and densely PEGylated low- and high-avidity ligands adopting a fully stretched brush-like PEG conformation (type C).

α_V_β_3_ integrin is a heterodimeric transmembrane receptor that is crucial for cell adhesion.^18–20^ The fact that the α_V_β_3_ integrin receptor is overexpressed in both tumoral endothelium and various tumor cells has made it a preferential target for active tumor targeting. Upon binding of a ligand in the extracellular domain, clustering of α_V_β_3_ integrin receptors and intracellular signal transduction are initiated.^13,21,22^ The tripeptide motif of l-arginine–glycine–l-aspartate (RGD) and its higher-affinity cyclic peptide derivatives are α_V_β_3_ integrin-specific antagonists that have been widely used as targeting ligands in cancer nanomedicine.^18,19^ Here, using the cyclic RGD-d-phenylalanine–l-lysine (c(RGDfK)) as a targeting ligand, we prepared a series of multivalent ligands with PEG spacers (*i.e.*, targeted agents; **L_X_** and **H_X_**; see Fig. 1a and S1†) differing in their IFGs and avidity to systematically investigate the surface compositions beneficial for tumor targeting. Moreover, we used the synthetic precursors of multivalent ligands that are devoid of targeting ligands (*i.e.*, untargeted agents; **PL_X_** and **PH_X_**; see Fig. S1†) to examine the influence of IFGs on EPR-based tumor targeting.

## Results and discussion

### Design

For a proper evaluation, we intended to vary only the IFGs in designing our tumor-targeting agents and keep all other structural features similar (Fig. 1a and S1†), such as the hydrodynamic diameter (*ca.* 10 nm) and molar contents of functional moieties (*e.g.*, targeting ligand, imaging agents, and PEG spacer). A third-generation (G3) polyamidoamine (PAMAM) dendrimer^23–27^ was used as a nanocarrier that has a robust shape and size (*ca.* 2–3 nm (ref. 28) in diameter) and enables precise molecular characterization by NMR.^29,30^ All of our tumor-targeting agents were made to have two types of imaging agents, each in a small equimolar portion, as substituted to the dendrimer surface for *in vitro* and *in vivo* imaging: cyanine 5.5 (Cy5.5; *ca.* 0.5 out of 32 peripheral groups) and (tri-*n*-butylstannyl)benzoyl (TBSB) moiety as a precursor for *in situ* radioiodination (*ca.* 2 out of 32). The targeting ligand c(RGDfK) was attached to the terminus of a PEG spacer to impart conformational flexibility and extend the pitch for facile multivalent binding. For the IFG, a simple amine group (**NH_2_**; the bare surface group of the dendrimer), succinate (**SA**), tetra(ethylene glycol) (**TEG**), and a conventional long PEG chain (**PEG**; with a discrete molecular weight (MW) of *ca.* 1700 Da, *i.e.*, a polydispersity index of 1) were employed. To facilitate ligand binding, here the IFG **PEG** was shorter (*ca.* 13 nm for the fully stretched conformation; Fig. S2†) than the spacer unit (*ca.* 16 nm). Furthermore, portions of the amino groups were acetylated (**12Ac** and **19Ac**) to examine the effect of cationic strength on tumor targeting.

Our strategy of embedding functional groups in the interior of partially PEGylated tumor-targeting agents, which would normally be occupied by PEG groups or ligands attached through PEG spacers, may compromise the EPR or multivalent effect. Accordingly, we also prepared high-avidity ligands (**H_X_**) in which *ca.* 32% of the dendrimer surface groups were substituted with c(RGDfK) (10–11 out of 32 peripheral groups), in addition to the initial low-avidity ligands (**L_X_**) with *ca.* 14% c(RGDfK) (4–5 out of 32) (Table S1†). As illustrated in Fig. 1b, our multivalent ligands designed as such can be classified into one of three categories in terms of PEG density:^17,31^ low-avidity ligands with small IFGs (**NH_2_**, **12Ac**, **19Ac**, **SA**, and **TEG**) adopting a collapsed mushroom-like PEG conformation (type A); high-avidity ligands with small IFGs adopting a brush-like PEG conformation (type B); and densely PEGylated ligands (**L_PEG_** and **H_PEG_**) adopting a fully stretched brush-like PEG conformation (type C). Given that α_V_β_3_ integrin receptors oligomerize upon binding to a ligand,^13,18,21,22^ we envisioned that the binding of subsequent c(RGDfK) moieties from the same dendritic multivalent ligand would be feasible, particularly for the less sterically demanding types A and B. Moreover, with increasing numbers of ligand–receptor tethers formed, the distance between a multivalent ligand and the cell surface is expected to be shorter, potentially enhancing the influence of the IFGs on tumor targeting.

### Synthesis and characterization

The synthesis of our multivalent ligands was planned so that the shared structural components were attached earlier in one batch, and the varied IFGs were incorporated later after dividing into individual portions (Fig. S3–S8†). To prepare the low- and high-avidity ligands, azide-terminated PEG spacers were first implanted into the dendrimer surface while controlling the stoichiometry. The resulting PEGylated dendrimers, **PPL** and **PPH**, which were fractionated by size using preparative size-exclusion chromatography (SEC), had *ca.* 5 and 11 PEG groups attached, respectively, as determined by the analysis of NMR integration (Fig. S12 and Table S1†). Next, TBSB and Cy5.5 for imaging were attached sequentially in one batch, followed by the IFGs separately. For **PL_12Ac_** and **PL_19Ac_**, approximately 50% and 75%, respectively, of the residual surface amino groups in **PL_NH2_** were acetylated based on the NMR integration (Fig. S5, S14a, and S15a†). The targeting ligand c(RGDfK) was attached to the azido terminus of a PEG spacer in the final step by orthogonal strain-promoted cycloaddition reaction^32^ as an azadibenzocyclooctyne (DBCO) adduct, **DBCO–c(RGDfK)** (Fig. S4, S9–S11, and S30†), which, after two rounds, exhibited overall conversions of 79–98%. For all targeted agents (**L_X_** and **H_X_**), three sets of isomers were found in their ^1^H NMR spectra (Fig. S13–S23†): a regioisomeric pair in comparable amounts as major products (isomers A and B, Fig. S17†) and a minor isomer (isomer C) that is presumably a conformer of isomer A as determined by the analysis of COSY and NOESY spectra. The average MWs of our PAMAM dendrimer conjugates by MALDI mass spectrometry were slightly underestimated^29^ compared with those determined by NMR (Fig. S32 and S33 and Table S1†).

**Fig. 2:**
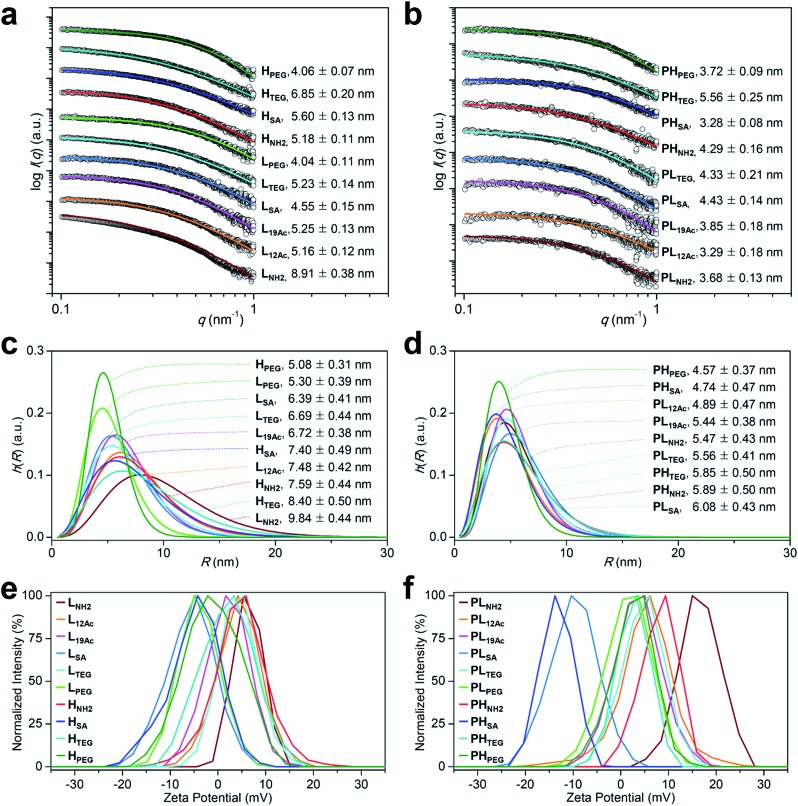
Physicochemical properties of our (a, c, and e) targeted (**L_X_** and **H_X_**) and (b, d, and f) untargeted agents (**PL_X_** and **PH_X_**). (a–d) Size (radius) estimated by SAXS at 25 °C in 2.5 mM NaCl solution (450 μM, pH 7.4; Table S2†). (a and b) The open symbols indicate experimental data and the solid lines indicate fits obtained using the SCATTER program. The values of *R*_g,G_ (radius of gyration; mean ± standard deviation (SD)) were estimated from the slope of the linear scattering data in the *q* ^2^-region using Guinier analysis. For clarity, each plot is shifted along the log *I*(*q*) axis. (c and d) Particle size distribution *h*(*R*) functions (homogeneous sphere model), which are characterized by the average sphere radius (*R*) and relative SD (*σ*_R_) obtained using the SCATTER program (listed as *R* ± *σ*_R_). (e and f) Surface charge measured as the zeta potential at 25 °C in 10 mM NaCl solution (100 μg mL^–1^, pH 7.4; Table S3†).

Next, the size of our tumor-targeting agents was investigated in an ionic aqueous solution similar to the physiological conditions. Our attempts to measure the hydrodynamic diameters of the targeted (**L_X_** and **H_X_**) and untargeted agents (**PL_X_** and **PH_X_**) by dynamic light scattering (DLS) failed, because Cy5.5 moieties apparently absorbed the irradiation light (633 nm) of the instrument used. Instead, the diameters of the PEGylated precursors without Cy5.5 (5.90 nm for **PPL** and 8.49 nm for **PPH**) and a fully PEGylated species (9.37 nm for **G3–32PEG**), which roughly represent types A, B, and C, respectively, as shown in Fig. 1b, were measured by DLS in an ionic aqueous solution (10 mM NaCl, pH 7.4, 25 °C; Fig. S31b†). Alternatively, we also estimated the size of our Cy5.5-substituted tumor-targeting agents by solution small-angle X-ray scattering (SAXS)^28,33^ (Fig. 2a–d and Table S2†). In general, the size of the targeted agents (mean radius of gyration (*R*_g,G_) of 4.04–8.91 nm; Fig. 2a and b) was larger than that of the untargeted counterparts (3.28–5.56 nm) without c(RGDfK) moieties. The increments in size (Δ*R*_g,G_) from the untargeted to the targeted species were the smallest for **L_SA_** (0.12 nm) and **H_PEG_** (0.34 nm), and the largest for **H_SA_** (2.32 nm) and, much more significantly, **L_NH2_** (5.23 nm). In fact, unlike the untargeted agents, the targeted agents with small IFGs appeared to aggregate (**L_NH2_** being the most drastic example) in an ionic aqueous solution (2.5 mM NaCl, pH 7.4, 25 °C), as shown by the size distribution profiles (homogeneous sphere model; Fig. 2c and d). In contrast, the fully PEGylated species (**H_PEG_**, **L_PEG_**, and **PH_PEG_**; type C in Fig. 1b), regardless of the presence of c(RGDfK), had the smallest sizes and the most symmetrical globular shapes, seemingly without any aggregation. Additionally, we examined the serum stability of our tumor-targeting agents—which are ultimately intended for intravascular administration—by measuring their sizes by SAXS in a solution (2.5 mM NaCl, pH 7.4, 25 °C) containing 10% (v/v) fetal bovine serum (FBS; Fig. S34 and S35†). Our preliminary studies indicated that all of our tested compounds retained their original sizes under the experimental conditions (incubation with FBS for 5 min at 25 °C), in which each compound and, for instance, bovine serum albumin (BSA; the most abundant protein in FBS)^34^ existing as separate entities (*i.e.*, the sum of the individual SAXS profiles coincided with that of the mixture).

We next estimated the surface charge (in zeta potential) of our tumor-targeting agents in an ionic aqueous solution (Fig. 2e and f and Table S3†). Interestingly, the magnitude of the charge of the untargeted agents (ranging from –13.3 mV for **PH_SA_** to 16.5 mV for **PL_NH2_**) was substantially attenuated in the targeted counterparts (ranging from –5.8 mV for **L_SA_** to 6.3 mV for **L_NH2_**), presumably due to the presence of c(RGDfK) moieties in the outermost layer. In this regard, we examined, by performing NOESY experiments in D_2_O, whether c(RGDfK) ligands in our targeted agents (*i.e.*, multivalent ligands) are interacting with any interior moieties such as the dendrimer segments through backfolding.^35^ As shown in Fig. S24–S29,† indeed no NOE cross-peaks were detected between the peaks of PAMAM and c(RGDfK) moieties in the NOESY spectra, suggesting that the c(RGDfK) ligands are likely positioned at the exterior for effective binding to receptors.

### *In vitro* studies

The safety of our multivalent ligands was evaluated by cytotoxicity assay (Cell Counting Kit-8 (CCK-8)) using human malignant glioblastoma U87MG cells (Fig. S36†). Although our tumor-targeting agents are intended for *in vivo* administration under microdosing conditions after radioiodination, a relatively high cell survival rate (*ca.* 60–90%) was achieved when the cells were directly exposed to our compounds at 0.1 μM for up to 72 h.

**Fig. 3:**
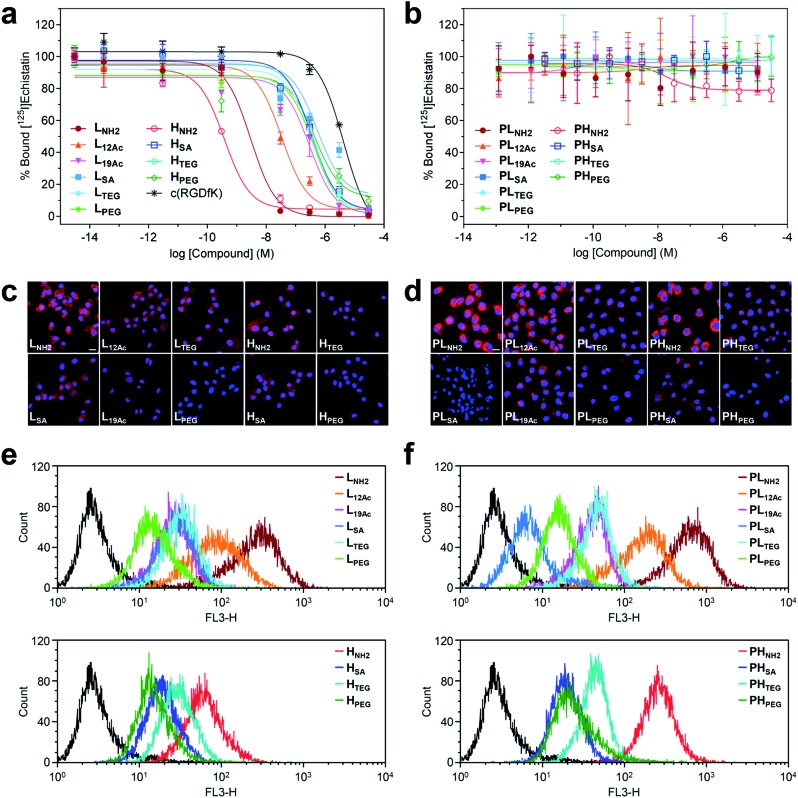
Results of *in vitro* assays on U87MG cells using our (a, c, and e) targeted (**L_X_** and **H_X_**) and (b, d, and f) untargeted agents (**PL_X_** and **PH_X_**). (a and b) Inhibitory effect of our nano-sized agents on the binding of [^125^I]echistatin to α_V_β_3_ integrin receptors expressed on U87MG cells. The IC_50_ values are listed in Table S4.† (c and d) Confocal laser fluorescence micrographs (400 × magnification) of U87MG cells incubated with each compound (1.8 μM) for 24 h at 37 °C in culture media. Cy5.5: red fluorescence; DAPI: blue fluorescence. Scale bars: 20 μm. (e and f) Flow cytometry histograms obtained from U87MG cells incubated with each compound (1.8 μM) for 24 h at 37 °C in culture media (control: black line).

Next, the binding strength of our multivalent ligands at α_V_β_3_ integrin receptors was measured by a competitive binding assay against the radiolabeled echistatin, an α_V_β_3_-specific antagonist, using U87MG cells^36,37^ (Fig. 3a and Table S4†). Strikingly, a high-avidity ligand with amine as the IFG, **H_NH2_**, exhibited a sub-nanomolar IC_50_ value of 3.77 × 10^–10^ M, which was more than 10^4^-fold enhancement over the monovalent control, c(RGDfK), with an IC_50_ of 4.22 × 10^–6^ M under the same conditions. This corresponds to more than 10^3^-fold enhancement in IC_50_ per ligand, considering that **H_NH2_** has *ca.* 10 c(RGDfK) moieties, as determined by NMR analysis (Fig. S20b and Table S1†). A low-avidity analog, **L_NH2_**, with approximately four c(RGDfK) moieties was the runner-up with about an order of magnitude lower IC_50_ of 2.95 × 10^–9^ M. Similarly, **L_12Ac_**, in which 50% of the amine (IFG) in **L_NH2_** was acetylated, displayed *ca.* 100-fold enhancement in IC_50_ against c(RGDfK), whereas all other multivalent ligands exhibited more or less the same IC_50_ values of only about 10-fold enhancement, irrespective of the type of IFG or avidity. Essentially, none of the untargeted counterparts without the c(RGDfK) moieties (**PL_X_** and **PH_X_**) were found to bind to α_V_β_3_ integrin receptors under the same assay conditions (Fig. 3b). Taken together, the α_V_β_3_-specific multivalent binding of our targeted agents was truly in effect (**H_NH2_** > **L_NH2_**), and the cooperative impact of the IFGs upon ligand–receptor binding was evident (**L_NH2_** > **L_12Ac_** > **L_19Ac_**).

Intriguingly, the cellular uptake profiles of our targeted and untargeted agents using U87MG cells, as investigated by confocal fluorescence microscopy (Fig. 3c and d and S37†) and quantitatively by flow cytometry (Fig. 3e and f), were significantly different from the results of competitive binding assays, which only considered the binding strength at the specific target (*i.e.*, the α_V_β_3_ integrin receptor). Obviously, in the binding assay, a higher avidity ligand with a favorable electrostatic-interaction potential (*i.e.*, amine as the IFG) and less steric issues for tighter binding to the cellular surface exhibited the lowest IC_50_ value (**H_NH2_**), as proposed in Fig. 1b (Type B). In contrast, in the confocal fluorescence micrographs, the untargeted agents, particularly with amine as the IFG, exhibited much stronger Cy5.5 fluorescence than their targeted counterparts (**PL_NH2_** > **L_NH2_**; **PH_NH2_** > **H_NH2_**). Indeed, the efficiency of internalization into the cells (**PL_NH2_** > **L_NH2_** ≥ **PH_NH2_** ≥ **PL_12Ac_** > **L_12Ac_** ≥ **H_NH2_**) appeared to depend more on the order of cationic strength^38^ (zeta potential: **PL_NH2_** > **PH_NH2_** > **L_NH2_** > **PL_12Ac_** > **H_NH2_** ≈ **L_12Ac_**) rather than on the degree (or presence) of avidity. In general, internalization was not efficient for the nano-sized agents with **SA** and **PEG** as the IFGs. As illustrated in the flow cytometry histograms (Fig. 3e and f), the difference between the highest and lowest fluorescence intensity levels (*i.e.*, the internalization efficiency) exhibited by the two extreme examples of untargeted agents declined substantially in their targeted counterparts: a tendency similar to that found in the surface charge profiles (Fig. 2e and f). In particular, the fluorescence intensities exhibited by the four high-avidity ligands were similar to each other and were relatively weak even for the strongest binder (**H_NH2_**) compared with that by the untargeted counterpart (**PH_NH2_**). The fluorescence intensity as measured by flow cytometry, however, cannot differentiate between the signals from surface-bound fluorophores and those from internalized fluorophores. At the time point of 24 h after incubating the cells with our targeted agents (when these confocal micrographs and flow cytometry results were obtained), Cy5.5 fluorescence from the surface-bound agents, potentially arising from specific ligand–receptor interactions, was not observed, as confirmed by the magnified confocal micrographs (Fig. S37†). In fact, at earlier time points (*e.g.*, 1 h after incubation), these adherent U87MG cells were somewhat unstable (or detached; data not shown), presumably due to the strong association with many of these multivalent ligands simultaneously. Although further investigations are needed, we envision that high-affinity multivalent ligands may more likely be retained on the cellular surface as opposed to entering into the cells,^5,35,39,40^ leading to more probable dissociation (*i.e.*, being washed off) from the cells over time, eventually to result in lower internalization efficiency compared with low-affinity ligands or untargeted agents.

### *In vivo* studies

**Fig. 4:**
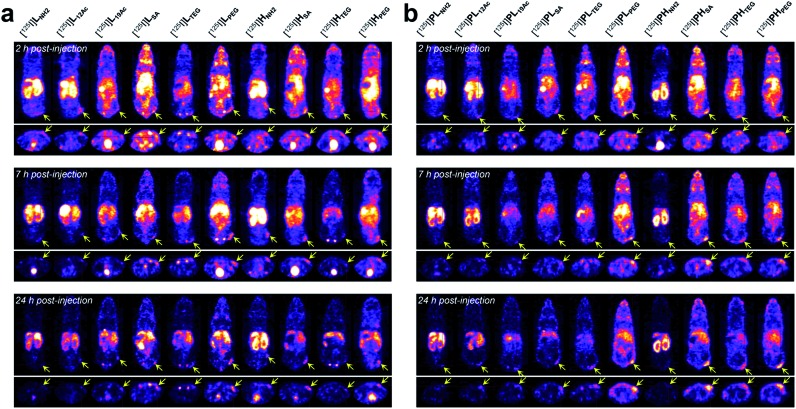
*In vivo* tumor-targeting profiles of our nano-sized dendritic agents by SPECT imaging. Mice (BALB/c nude, female) bearing U87MG tumors were injected with (a) targeted (**L_X_** and **H_X_**) and (b) untargeted agents (**PL_X_** and **PH_X_**) radiolabeled with iodine-125, and their SPECT images (top: coronal views; bottom: axial views) were obtained at 2 hpi, 7 hpi, and 24 hpi. See Fig. S38 and Movies S1 and S2† for details.

Next, the tumor-targeting efficiency of our nano-sized dendrimer conjugates was evaluated using U87MG tumor-bearing mice by single-photon emission computed tomography (SPECT) imaging.^37,41^ To this end, the TBSB moieties in both targeted and untargeted agents were substituted with γ-ray-emitting iodo groups *in situ* (radiolabeling efficiency of >95%), and the resulting ^125^I-labeled agents were intravenously injected. A clear discrepancy in the tumor radioactivity level was observed among our radiolabeled nano-sized agents with varied IFGs and avidities (or PEG density) (Fig. 4, S38, and S39, and Movies S1 and S2†). Most notably, the high-avidity ligand **H_SA_** with the anionic IFG and its untargeted counterpart **PH_SA_** with the most negatively charged surface as determined by the zeta potential measurements, manifested unexpectedly intense tumor signals in the SPECT images. Interestingly, for the anionic agents, tumor targeting was more effective for those with higher PEG density (**PH_SA_** and **H_SA_** > **PL_SA_** and **L_SA_**). Tumor imaging was also enabled by nano-sized agents with **PEG** as the IFG, but with somewhat weaker intensity than that by anionic agents. Here, the time-course profiles of two fully PEGylated untargeted agents, **PL_PEG_** and **PH_PEG_**, were nearly identical, suggesting that the presence of terminal azide groups did not practically affect the *in vivo* behavior (Fig. S1†). In contrast, the cationic high-avidity ligand **H_NH2_**, which performed best in the *in vitro* binding assay, exhibited only modest tumor-targeting efficiency, and other cationic agents (**L_NH2_**, **PL_NH2_**, and **PH_NH2_**) were essentially ineffective. For these amine-containing agents, predominant uptake in the kidney, as well as in the liver, was detected explicitly from earlier time points (a greater extent in the kidney than in the liver: **PH_NH2_** > **H_NH2_** > **PL_NH2_** and **L_NH2_**), apparently causing dramatically low net availability (*i.e.*, concentration) in the vascular system for tumor targeting. In general, off-target uptake^3–5,42,43^ in the liver was more pronounced for the targeted agents than for the untargeted agents. Strong uptake in the kidney was also observed for **L_12Ac_** and **PL_12Ac_**, both with relatively high amine contents, and more significantly for the anionic agent **L_SA_**. Uptake in the kidney of the high-avidity anionic analog **H_SA_** was also observed, but this was much less prominent than for **L_SA_**, and was not detected for the untargeted anionic counterparts, **PL_SA_** and **PH_SA_**. The tumor-targeting efficiency of nano-sized agents with **TEG**—a small and neutral IFG—was either modest (**PH_TEG_**) or low (**L_TEG_**, **H_TEG_**, and **PL_TEG_**). In fact, the *in vivo* clearance of these **TEG**-containing agents appeared to occur more rapidly than that of the agents with other IFGs, as evidenced by their relatively low uptake in major organs (*e.g.*, liver, spleen, and kidney). Overall, tumor targeting was more successful at earlier time points (*e.g.*, 2 h post-injection (hpi)) for the targeted agents and at later time points (*e.g.*, 24 hpi) for the untargeted agents. This was in agreement with other relevant studies on tumor imaging using nano-sized agents for active and passive targeting.^16,44^ To summarize our findings, for a successful *in vivo* tumor targeting using nano-sized agents, it appeared that prolonging the blood circulation time is a prerequisite, as demonstrated by the agents with **SA** or **PEG** as the IFG. Unlike the PEG groups, which are used frequently to extend blood half-lives, we speculate that tumor targeting by those with the anionic IFG was effective probably because of their lower propensity to adsorb proteins (to form protein corona^34,45–48^) upon intravascular injection, leading to lower macrophage uptake. Furthermore, a wide discrepancy in the tumor-targeting efficiency was demonstrated between the two anionic untargeted agents, presumably because **PH_SA_** with a higher PEG density (*ca.* 35%) more effectively blocked protein adsorption than **PL_SA_** (*ca.* 16%).^49,50^ This clearly indicates that there is a subtle physicochemical preference for the optimal stoichiometric composition of surface functionalities. Finally, it is interesting to note that whereas the tumor accumulation of the fully PEGylated untargeted agents, **PL_PEG_** and **PH_PEG_**, gradually increased over time (*i.e.*, the EPR effect), that of the partially PEGylated (*ca.* 35%) and anionic **PH_SA_** was already evident at the earlier time point (2 hpi). Considering that these untargeted agents had similar hydrodynamic sizes (mean *R*_g,G_ by SAXS of 3.3 nm for **PH_SA_** *vs.* 3.7 nm for **PH_PEG_**; Fig. 2b) and similar profiles of off-target uptake at 2 hpi, this may conceivably indicate that derivatizing the partially PEGylated surfaces of nanocarriers with a small and negatively charged IFG (**SA**; *ca.* 57% of the surface) could substantially accelerate the extravasation process to reach the tumor interstitium compared with PEGylation of the entire surface.

**Fig. 5:**
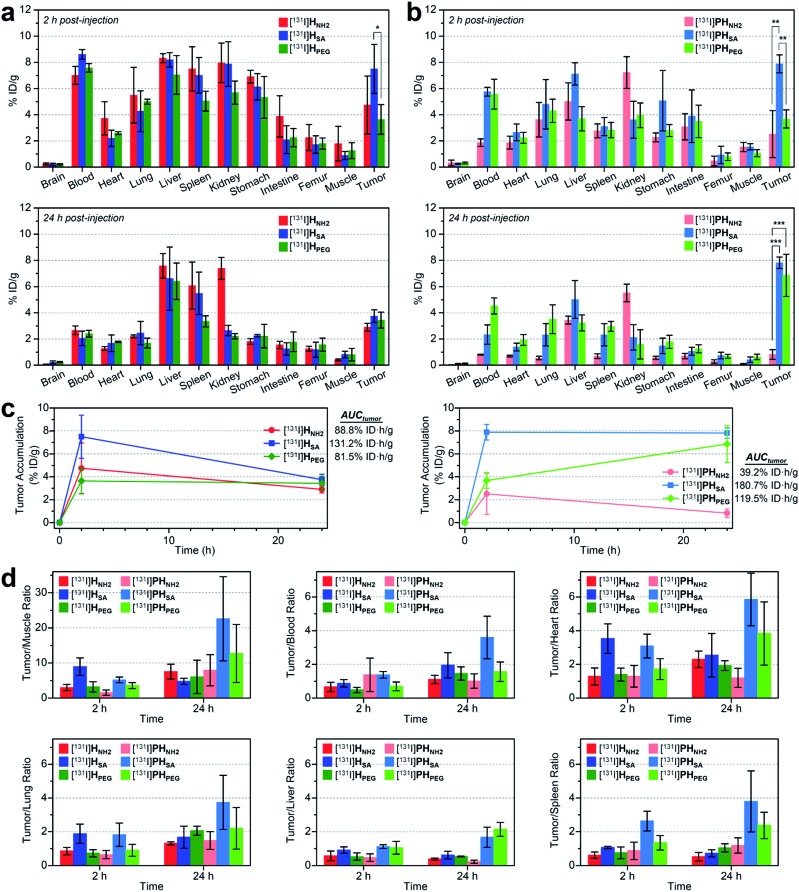
(a and b) Biodistribution (mean ± SD, *n* = 3) of selected nano-sized dendritic agents at 2 hpi and 24 hpi (Table S5†). Mice (BALB/c nude, female) bearing U87MG tumors were injected with (a) high-avidity ligands (targeted agents) and (b) their untargeted counterparts (synthetic precursors without c(RGDfK)) radiolabeled with iodine-131. Statistical analysis was performed by an unpaired *t*-test (*p < 0.05, **p < 0.01, and ***p < 0.001). (c) Tumor-targeting efficiency estimated quantitatively as the area-under-the-curve values (AUC_tumor_; total time period: 24 h) based on the non-compartmental linear trapezoidal analysis model.^3^ For all compounds, one additional time point, 0 hpi (*i.e.*, before injection, 0% ID g^–1^), was considered for the estimation of the AUC_tumor_. (d) Relative tumor-targeting efficiency estimated as the tumor-to-organ ratio (mean ± SD, *n* = 3; Table S6†).

We also conducted biodistribution studies using selected nano-sized agents at two time points (2 hpi and 24 hpi) following intravenous injection (Fig. 5a and b and Table S5†). To this end, three high-avidity ligands (**H_NH2_**, **H_SA_**, and **H_PEG_**) and their untargeted counterparts (**PH_NH2_**, **PH_SA_**, and **PH_PEG_**), which displayed the most dramatic profiles in SPECT imaging, were chosen. Quantitative results acquired using ^131^I-labeled agents corroborated our findings from SPECT imaging: (1) anionic agents were most effective in tumor localization (*ca.* 8% ID g^–1^) for both targeted and untargeted strategies; (2) for passive targeting, deposition of **PH_SA_** at tumors was obvious from the earlier time point (7.89% ID g^–1^ at 2 hpi *vs.* 7.82% ID g^–1^ at 24 hpi), whereas **PH_PEG_** exhibited a typical EPR-based profile (3.68% ID g^–1^ at 2 hpi *vs.* 6.85% ID g^–1^ at 24 hpi); (3) off-target uptake was generally higher for the targeted agents than for the untargeted agents; and (4) marked kidney uptake was verified for the cationic agents at both 2 hpi and 24 hpi.

To quantitatively assess the tumor-targeting efficiency, we determined the total tumor accumulation over a period of 24 h as the area-under-the-curve values (AUC_tumor_)^3^ by including pre-contrast intensities (*i.e.*, no tumor radioactivity at 0 hpi; Fig. 5c). Here, the superior tumor-targeting efficiency of the anionic agents was unequivocally confirmed for both targeted (131.2% ID h g^–1^ for **H_SA_**) and untargeted groups (180.7% ID h g^–1^ for **PH_SA_**), which was both 50% or more effective than their fully PEGylated counterparts (81.5% ID h g^–1^ for **H_PEG_** and 119.5% ID h g^–1^ for **PH_PEG_**). Moreover, whereas the cationic multivalent ligand **H_NH2_** with the highest affinity for active targeting performed only marginally better than the fully PEGylated analog **H_PEG_**, the cationic agent **PH_NH2_** for passive targeting was virtually ineffective. This is likely due to the high off-target uptake, particularly in the kidney and liver, of the cationic agents (Table S5†). The excellent tumor-targeting efficiency of the high-avidity (or high PEG density) anionic agents was further validated by estimating the accumulation in the tumor with respect to that in other organs (Fig. 5d and Table S6†). Notably, the tumor-to-muscle (*i.e.*, background) ratio^30^ of the anionic agents was higher at 2 hpi for the targeted agent (**H_SA_**, 8.94 at 2 hpi *vs.* 4.75 at 24 hpi) and at 24 hpi for the untargeted agent (**PH_SA_**, 5.17 at 2 hpi *vs.* 22.58 at 24 hpi). For the targeted agents, owing to their relatively high concentration in the blood at 2 hpi (7.01–8.61% ID g^–1^), their tumor-to-blood ratios were higher at 24 hpi than at 2 hpi. The highest tumor-to-blood ratio was achieved by the anionic untargeted agent **PH_SA_** at 24 hpi (3.59), which was nearly twice as high as that of its targeted counterpart **H_SA_**. In fact, **PH_SA_** excelled in most tumor-to-organ ratios relevant to commonly known off-target sites for nanocarriers (*e.g.*, liver, spleen, and lung), suggesting that minimizing the off-target uptake is imperative for the success of nanocarrier-based tumor targeting.

## Conclusions

In summary, to rationally improve the conventional design of nanocarriers for the active or passive tumor targeting by a systematic approach, functional groups that varied in their electronic and steric properties were embedded in the interior of partially PEGylated nanocarriers. To ensure a reliable structure–activity relationship, all of our compounds were made while strictly controlling the stoichiometry and homogeneity, and were characterized precisely using NMR. The influence of the IFGs was demonstrated explicitly, although with contrasting efficacies *in vitro* and *in vivo*. In the *in vitro* setting, nanocarriers were directly exposed to tumor cells, and, as expected, those with the cationic IFG for favorable electrostatic interactions performed best in both multivalent binding (**H_NH2_** and **L_NH2_**) and internalization into cells (**PL_NH2_**, **L_NH2_**, and **PH_NH2_**). Conversely, upon intravascular administration, severe off-target accumulation (in the liver and kidney) was observed for nanocarriers with cationic IFGs, essentially being ineffective for the *in vivo* tumor targeting purposes. Anionic agents of high-avidity (**H_SA_**) and high PEG density (**PH_SA_**; *ca.* 35% of the surface), which exhibited modest or poor efficiencies comparable to those of fully PEGylated species *in vitro*, rather demonstrated superb tumor-targeting efficiency *in vivo*, which is presumably due to their low off-target uptake, prolonged blood circulation, and/or fast extravasation process relative to those of other nano-sized agents. Consequently, in order to enhance the accuracy and efficacy of tumor targeting for clinically desirable diagnostic and therapeutic effects, it appears to be a promising endeavor to design the PEG surface of nanocarriers with similar anionic compositions (**H_SA_** and **PH_SA_**), which could be converted into a cationic surface upon reaching the tumor site^51–53^ for (i) strong and selective multivalent binding (**H_NH2_** and **L_NH2_**; *e.g.*, therapeutic effects by binding to the surface receptors), (ii) effective internalization (**PL_NH2_**, **L_NH2_**, and **PH_NH2_**; *e.g.*, intracellular anticancer drug/gene delivery), or (iii) both (**L_NH2_**). Of note, whereas active targeting is important to achieve specificity (*i.e.*, selectivity), the internalization of the strongest multivalent binder **H_NH2_** (sub-nanomolar IC_50_) into tumor cells was less efficient than that of a cationic ligand of lower avidity (**L_NH2_**) or its untargeted counterpart (**PH_NH2_**). This may be more critical when receptors (*e.g.*, α_V_β_3_ integrin receptor) present in both the tumoral endothelium and the tumoral interstitium (tumor cells) are targeted by a single multivalent ligand.^5,40^ Although further studies are needed and our findings may be more applicable to relatively small (*ca.* 10 nm) organic dendritic nanocarriers, we have demonstrated that the tumor-targeting efficiency of commonly used, fully PEGylated nanocarriers can be improved significantly by replacing some of the PEG groups with properly chosen IFGs. Given the need for surface PEGylation, we envision that our strategy may be useful in various fields of nanomedicine.
