# Supplementary material for: Toward redesigning the PEG surface of nanocarriers for tumor targeting: impact of inner functionalities on size, charge, multivalent binding, and biodistribution
Source: Chem Sci. 2017 Apr 20;8(7):5186–95. doi: 10.1039/c6sc05640g (PMC5618790; doi:10.1039/c6sc05640g)
Supplement: Supplementary file 1 [file SC-008-C6SC05640G-s001.pdf]

Electronic Supplementary Material (ESI) for Chemical Science.
This journal is © The Royal Society of Chemistry 2017

Electronic Supplementary Information for *Chemical Science*

Toward redesigning the PEG surface of nanocarriers for tumor targeting: impact of inner functionalities on size, charge, multivalent binding, and biodistribution

Ju Young Heo,[†ab] Se Hun Kang,[†c] Young-Hwa Kim,[†de] Suyeon You,[†a] Kyeong Sik Jin,[†f] Seung Won Kim,[c] Hye-youn Jung,[a] Kyung Oh Jung,[de] Chul-Hee Lee,[de] Mi Jung Kim,[a] Soo-Eun Sung,[a] Boram Kim,[a] Insung S. Choi,[b] Hyewon Youn,[deg] June-Key Chung,*[deg] Seok-ki Kim*[c] and Yoonkyung Kim*[ah]

[a] Korea Research Institute of Bioscience and Biotechnology, Daejeon, 34141, Korea
[b] Department of Chemistry, Korea Advanced Institute of Science and Technology, Daejeon, 34141, Korea
[c] Molecular Imaging and Therapy Branch, National Cancer Center, Goyang, 10408, Korea
[d] Department of Biomedical Sciences, Seoul National University College of Medicine, Seoul, 03080, Korea
[e] Cancer Research Institute, Seoul National University College of Medicine, Seoul, 03080, Korea
[f] Pohang Accelerator Laboratory, Pohang University of Science and Technology, Pohang, 37673, Korea
[g] Department of Nuclear Medicine, Seoul National University Hospital, Seoul, 03080, Korea
[h] Korea University of Science and Technology (UST), Daejeon, 34113, Korea
[†] These authors contributed equally to this work.
*E-mail: jkchung@snu.ac.kr; skkim@ncc.re.kr; ykim@kribb.re.kr.

Table of Contents

Experimental Section	pp. 3-27
**Table S1.** Structural analysis of synthesized dendrimer conjugates by NMR and MALDI-TOF MS.	p. 28
**Table S2.** Structural parameters obtained from SAXS data of targeted and untargeted agents in aqueous solution.	p. 29
**Table S3.** Surface charge of targeted and untargeted agents.	p. 30
**Table S4.** IC_{50} values of multivalent dendritic ligands and c(RGDfK) against binding of [^{125}I]echistatin to the $\alpha_v\beta_3$ integrin receptors on U87MG cells.	p. 31
**Table S5.** Biodistribution (% ID/g) of selected tumor-targeting agents radiolabeled with iodine-131.	p. 32
**Table S6.** Tumor-to-organ ratios of selected tumor-targeting agents.	p. 33
**Fig. S1.** Schematic illustrations of targeted and untargeted agents.	p. 34
**Fig. S2.** Comparison of the length of the IFG **PEG** and the spacer unit in the multivalent ligands by computer modeling.	p. 35
**Fig. S3.** Synthetic scheme to prepare ***p*NP-PEG-N$_3$**.	p. 36
**Fig. S4.** Synthetic scheme to prepare **DBCO-c(RGDfK)**.	p. 36
**Fig. S5.** Synthetic scheme to prepare precursors of six low-avidity ligands.	p. 37
**Fig. S6.** Synthetic scheme to prepare six low-avidity ligands.	p. 38
**Fig. S7.** Synthetic scheme to prepare precursors of four high-avidity ligands.	p. 39
**Fig. S8.** Synthetic scheme to prepare four high-avidity ligands.	p. 40
**Fig. S9-S11.** ^{1}H NMR, COSY, and NOESY spectra of c(RGDfK), DBCO-NHS ester, **DBCO-c(RGDfK)** in DMSO-*d*$_6$.	pp. 41-43
**Fig. S12.** ^{1}H NMR spectra of **PPL** and **PPH**.	p. 44
**Fig. S13-S16 & S18-S19.** ^{1}H NMR spectra of **PL$_X$** and **L$_X$** in DMSO-*d*$_6$.	pp. 45-48 & 50-51
**Fig. S17.** COSY and NOESY spectra of **L$_{SA}$** in DMSO-*d*$_6$.	p. 49
**Fig. S20-S23.** ^{1}H NMR spectra of **PH$_X$** and **H$_X$** in DMSO-*d*$_6$.	pp. 52-55
**Fig. S24-S29.** NOESY spectra of **L$_{NH2}$**, **L$_{SA}$**, **L$_{PEG}$**, **H$_{NH2}$**, **H$_{SA}$**, and **H$_{PEG}$** recorded in D_2O.	pp. 56-61
**Fig. S30.** ESI HRMS spectra of **DBCO-c(RGDfK)** and ***p*NP-PEG-N$_3$**.	p. 62
**Fig. S31.** Characterization of **PPL** and **PPH** by DLS and MALDI MS.	p. 63
**Fig. S32-S33.** MALDI-TOF mass spectra of untargeted and targeted agents.	pp. 64-65
**Fig. S34-S35.** SAXS profiles of untargeted and targeted agents with 10% FBS.	pp. 66-67
**Fig. S36.** Cytotoxicity assay results of targeted agents using U87MG cells.	p. 68
**Fig. S37.** Confocal laser fluorescence micrographs (1000×) of U87MG cells incubated for 24 h with **L$_{NH2}$**, **H$_{NH2}$**, **PL$_{NH2}$**, and **PH$_{NH2}$**.	p. 69
**Fig. S38.** SPECT images of mice injected with targeted agents at earlier time points.	p. 70
**Fig. S39.** Position of each organ marked in the SPECT images.	p. 71
**Movies S1 & S2.** Whole-body 3D SPECT images of mice injected with targeted and untargeted agents at 2 hpi, 7 hpi, and 24 hpi.	p. 72
References	p. 73

EXPERIMENTAL SECTION

Materials

Glassware was oven-dried and cooled to room temperature in a desiccator before use. All reactions were carried out under a dry argon (Ar) atmosphere. Solvents were purchased from Sigma-Aldrich as anhydrous grade and used without further purification. Suppliers of the commercial compounds are listed as follows: amine-terminated third generation polyamidoamine (G3 PAMAM) dendrimer with the ethylenediamine core was purchased from Dendritech; azido-dPEG®$_{36}$-alcohol, m-dPEG®$_4$-NHS ester (*N*-hydroxysuccinimide (NHS) ester of tetra(ethylene glycol) methyl ether; mTEG-NHS ester), and m-dPEG®$_{37}$-NHS ester (NHS ester of poly(ethylene glycol) methyl ether; mPEG-NHS ester) were purchased from Quanta BioDesign; 4-nitrophenyl chloroformate, trimethylamine, *N*,*N*-diisopropylethylamine (DIEA), acetic anhydride (Ac_2O), succinic anhydride, tetrahydrofuran (THF), *N*,*N*-dimethylformamide (DMF), and dimethyl sulfoxide (DMSO) were purchased from Sigma-Aldrich; DMSO-d_6, methanol-d_4 (CD_3OD), and deuterium oxide (D_2O) were purchased from Cambridge Isotope Laboratories; azadibenzocyclooctyne (DBCO)-PEG5-NHS ester (catalog no. A102P) was purchased from Click Chemistry Tools; cyclic Arg-Gly-Asp-(D-Phe)-Lys bistrifluoroacetic acid (c(RGDfK)·2TFA) (catalog no. FC-2204) was purchased from FutureChem; succinimidyl 3-(tri-*n*-butylstannyl)benzoate was purchased Texas Biochemicals; Cyanine5.5 (Cy5.5) NHS ester was purchased Lumiprobe.

General Methods

Analytical thin layer chromatography (TLC) was performed on 0.2 mm silica glass coated sheets (E. Merck) with F-254 indicator. Visualization of the products on TLC plate was performed by UV light, iodine (I_2), potassium permanganate ($KMnO_4$), and ninhydrin. Flash column chromatography was performed on Merck 40-63 μm silica gel. Preparative size-exclusion chromatography (SEC) was performed at ambient pressure on Bio-Beads S-X1 (exclusion limit 14000 Da, 200-400 mesh, Bio-Rad) with DMF as an eluent, Sephadex LH-20 (exclusion limit 4000-5000 Da, 18-111 μm, GE Healthcare) with methanol as an eluent, or Sephadex G-25 Medium (exclusion limit 5000 Da, 50 μm, GE Healthcare) with deionized water as an eluent.

Nuclear magnetic resonance (NMR) spectra were recorded on a Varian INOVA 600 spectrometer at 25.0 °C under an optimized parameter setting for each sample. ^{1}H NMR chemical shifts were measured relative to the residual solvent peak at 2.50 ppm in DMSO-d_6, at 7.26 ppm in $CDCl_3$, at 3.31 ppm in CD_3OD, and at 4.80 ppm in D_2O. Generally, complete peak assignments for ^{1}H NMR were made possible with 2D COSY and NOESY experiments. The peaks for the dendrimer conjugates were assigned using the labeling method shown in Fig. S12-S23 and S31c. The relative integration values of the PAMAM dendrimer conjugates were determined by normalizing the integral of a PAMAM methylene peak "c" at *ca.* 2.18 ppm (*i.e.*, an internal standard) in DMSO-d_6 to 120 H. The integration values of the dendrimer conjugates were reported only for the peaks clearly resolved (*i.e.*, with a relatively good baseline-separation) in the ^{1}H NMR spectra and in two decimal places. Here, the PAMAM dendrimer conjugates were not unimolecular, and thus the characterization based on the NMR integration represented the average value from its polymeric distribution. Additionally, the commercial G3 PAMAM dendrimer had a significant amount of structural defects,[1,2] and thus the average molecular weights (MWs) of its conjugates were generally overestimated by NMR integration (assuming theoretical 32 peripheral groups). Detailed methods for the structural analysis of PAMAM dendrimer conjugates by NMR were reported previously.[1] The stoichiometry and the average MWs of the dendrimer conjugates determined by NMR integration are summarized in Table S1.

Matrix-assisted laser desorption ionization time-of-flight mass spectrometry (MALDI-TOF MS) experiments were performed on an Applied Biosystems Voyager-DE STR spectrometer at the Daejeon Technopark Bio Venture Town. 2,5-Dihydroxybenzoic acid (DHB) was used as a matrix for the MALDI samples. The peak-average molar mass (M_p) values were assigned approximately at the center of the bandwidth at the half-height of each peak. Also, the number-average molar mass (M_n), weight-average molar mass (M_w), and polydispersity index (PDI, M_w/M_n) were calculated within the specified mass range around the major peaks (*i.e.*, calculated region) using the Data Explorer software (see Fig. S31d, S32, and S33). Electrospray ionization (ESI) MS experiments were performed on a Quadrupole time-of-flight (Q-TOF) Ultima mass spectrometer at the Mass Spectrometry Laboratory of the University of Illinois.

Small-angle X-ray scattering (SAXS) measurements were carried out at a power of 3 GeV using the 4C SAXS II beamline (BL) of the Pohang Light Source II (PLS II) at the Pohang University of Science and Technology. A light source from an In-vacuum Undulator 20 (IVU20: 1.4 m length, 20 mm period) of the PLS II storage ring was focused with a vertical focusing toroidal mirror coated with rhodium and monochromatized with a Si (111) double crystal monochromator (DCM), yielding an X-ray beam wavelength of 0.734 Å. The X-ray beam size at the sample stage was 0.1 (V) × 0.3 (H) mm^2. A two-dimensional charge-coupled detector (Mar USA, Inc.) was employed, and the sample-to-detector distance (SDD) was 4.00 m. The magnitude of the scattering vector, $q = (4\pi/\lambda) \sin \theta$, was $0.10\ nm^{-1} < q < 1.00\ nm^{-1}$, where 2θ is the scattering angle and λ is the wavelength of the X-ray beam source. The scattering angle was calibrated with a polystyrene-*b*-poly(ethylene-*co*-butylene)-*b*-polystyrene (SEBS) block copolymer standard. For the solution sample cell, a quartz capillary with an outside diameter of 1.5 mm and wall thickness of 0.01 mm was used. All scattering measurements were carried out at 25 °C. The SAXS data were collected in 10 s. Our sample solutions (450 μM, 40 μL) for SAXS experiments were prepared by mixing 30 μL (18 nmol) of stock solutions (600 μM) of targeted ($\mathbf{L_X}$ and $\mathbf{H_X}$) or untargeted ($\mathbf{PL_X}$ and $\mathbf{PH_X}$) agents in deionized water and 10 μL of an aqueous solution of NaCl (10 mM, pH 7.4). Each 2D SAXS pattern was radially averaged from the beam center and was normalized to the transmitted X-ray beam intensity, which was monitored with a scintillation counter placed behind the sample. The scattering of a blank solution (*i.e.*, 2.5 mM NaCl, pH 7.4) was used as the experimental background. The $R_{g,G}$ (radius of gyration) values were estimated from the slope of the linear scattering data in the q^2-region using Guinier analysis[3] (Fig. 2a,b and Table S2). The SCATTER program was used for the analysis of one-dimensional X-ray scattering data, providing more detailed information on particle shape, size, and size distribution[4,5] (Fig. 2c,d). Additionally, the serum stability of our tumor-targeting agents (450 μM) was examined by measuring the size by SAXS similarly in the solution (2.5 mM NaCl solution, pH 7.4, 25 °C) containing 10% (v/v) fetal bovine serum (FBS; incubation time: 5 min). Extending the incubation time (1 h, 2 h, and 4 h) with FBS using one of our compounds ($\mathbf{H_{SA}}$; data not shown) did not alter the SAXS-estimated size. The results are shown in Fig. S34 and S35.

The hydrodynamic diameters were measured at 25.0 °C by dynamic light scattering (DLS; scattering angle: 90°) using a Zetasizer Nano ZS90 from Malvern Instruments. Three independent measurements (*n* = 3) were made on separately prepared samples (at 2 mg/mL in 10 mM NaCl solution, pH 7.4). The numerical results are shown as the mean ± standard deviation (SD) in Fig. S31b.

The zeta potentials were measured to determine the surface charge of dendrimer conjugates at 25.0 °C using a Zetasizer Nano ZS90 from Malvern Instruments. Three independent measurements (*n* = 3) were made using separately prepared samples (at 100 μg/mL in 10 mM NaCl, pH 7.4). The overlaid representative plots of targeted and untargeted agents and the numerical results are shown as the mean ± SD in Fig. 2e,f and Table S3, respectively.

Synthesis of *p*NP-PEG-N_3

Azido-dPEG®$_{36}$-alcohol (**1**, 935 mg, 0.574 mmol) and 4-nitrophenyl chloroformate (**2**, 233 mg, 1.12 mmol) was dissolved in THF (57 mL), to which triethylamine (0.160 mL, 1.15 mmol) was added slowly. The reaction mixture was stirred at room temperature for 41 h under a dry Ar atmosphere. After removal of the solvent under reduced pressure, the crude mixture was loaded on a size-exclusion chromatography (SEC) column (Bio-Beads S-X1, H 41 cm × O.D. 4.5 cm) in DMF for purification. The SEC column fractions confirmed to contain the desired product by the analysis of ^{1}H NMR were combined to give 436 mg of ***p*NP-PEG-N_3** (0.243 mmol, 42%) as a pale yellowish solid. For ^{1}H NMR peak assignments, see Fig. S3 for the labeling method. The desired azido derivative, ***p*NP-PEG-N_3**, was obtained as contaminated with the unreacted starting material, azido-dPEG®$_{36}$-alcohol, which was difficult to remove by SEC. Thus, the molar content of the desired compound was calculated based on the ^{1}H NMR integration for its stoichiometrically controlled addition to the reactions to prepare **PPL** and **PPH**. ^{1}H NMR (600 MHz, $CDCl_3$) δ 8.27 (d, 2H, J = 9.0 Hz, H_{8NP}), 7.39 (d, 2H, J = 9.1 Hz, H_{7NP}), 4.43 (m, 2H, H_{6NP}), 3.81 (m, 2H, H_{5NP}), 3.79-3.48 (m, 138H, H_{2NP}, H_{3NP}, and H_{4NP}), 3.38 (t, 2H, J = 5.0 Hz, H_{1NP}); HRMS (ESI) Calcd for $C_{79}H_{148}N_4O_{40}Na$ $(M + Na)^+$: 1815.9568, Found: 1815.9537.

Synthesis of DBCO-c(RGDfK)

To a solution of DBCO-PEG5-NHS ester (118 mg, 0.169 mmol) and c(RGDfK)·2TFA (142 mg, 0.171 mmol) in DMSO-d_6 (1.6 mL) was slowly added DIEA (91 μL, 0.52 mmol). The reaction mixture was stirred at room temperature for 5 h under a dry Ar atmosphere and concentrated under reduced pressure. The crude mixture was chromatographed on a silica gel column (70:30:6 CH_2Cl_2/MeOH/H_2O) to give 138 mg (117 μmol, 69%) of **DBCO-c(RGDfK)**. See Fig. S11 for ^{1}H NMR peak assignments. R_f 0.45 [silica gel, 70:30:6 CH_2Cl_2/MeOH/H_2O]; ^{1}H NMR (600 MHz, DMSO-d_6) δ 8.50 (d, 1H, J = 8.6 Hz, H_1), 8.25 (m, 1H, H_{11}), 8.24 (m, 1H, H_9), 8.21 (m, 1H, H_6), 8.20 (m, 1H, H_{21}), 7.89 (t, 1H, J = 5.5 Hz, H_{27}), 7.70 (d, 1H, J = 5.6 Hz, H_{15}), 7.69 (t, 1H, J = 5.5 Hz, $H_{12'}$), 7.63 (d, 1H, J = 8.0 Hz, $H_{8'}$), 7.59 (m, 1H, $H_{1'}$), 7.49 (m, 1H, $H_{3'}$/$H_{4'}$), 7.46 (m, 2H, $H_{3'}$/$H_{4'}$ and $H_{2'}$), 7.39 (td, 1H, J = 7.4, 1.5 Hz, $H_{7'}$), 7.35 (td, 1H, J = 7.2, 1.0 Hz, $H_{6'}$), 7.30 (dd, 1H, J = 7.5, 1.1 Hz, $H_{5'}$), 7.22 (t, 2H, J = 7.5 Hz, H_{19}), 7.17 (d, 2H, J = 7.3 Hz, H_{18}), 7.15 (t, 1H, J = 7.2 Hz, H_{20}), 7.28-6.91 (m, 4H, H_7 and H_8), 5.04 (d, 1H, J = 14.0 Hz, $H_{9eq'}$), 4.65 (td, 1H, J = 9.4, 5.6 Hz, H_{16}), 4.53 (q, 1H, J = 7.2 Hz, H_2), 4.34 (q, 1H, J = 7.6 Hz, H_{22}), 4.17 (m, 1H, H_{12}), 4.15 (m, 1H, H_{10}), 3.63 (d, 1H, J = 14.1 Hz, $H_{9ax'}$), 3.58 (t, 2H, J = 6.6 Hz, $H_{23'}$), 3.50-3.39 (m, 18H, $H_{14'}$, $H_{15'}$, $H_{16'}$, $H_{17'}$, $H_{18'}$, $H_{19'}$, $H_{20'}$, $H_{21'}$, and $H_{22'}$), 3.34 (m, 1H, H_{17}), 3.09 (m, 1H, $H_{11'}$), 3.09 (q, 2H, J = 6.5 Hz, H_5), 2.98 (m, 2H, H_{26}), 2.94 (m, 1H, $H_{11'}$), 2.74 (d, 1H, J = 15.2 Hz, H_{13}), 2.61 (m, 1H, H_{17}), 2.42 (m, 1H, $H_{10'}$), 2.30 (t, 2H, J = 6.5 Hz, $H_{24'}$), 2.17 (t, 2H, J = 6.5 Hz, $H_{13'}$), 2.06 (d, 1H, J = 15.7 Hz, H_{13}), 1.80 (m, 1H, $H_{10'}$), 1.69 (m, 2H, H_3), 1.63, 1.58 (m, 2H, H_{23}), 1.53, 1.46 (m, 2H, H_4), 1.37 (m, 2H, H_{25}), 1.24, 1.17 (m, 2H, H_{24}); HRMS (ESI) Calcd for $C_{59}H_{80}N_{11}O_{15}$ $(M + H)^+$: 1182.5835, Found: 1182.5829.

Synthesis of PPL

The methanolic solution of G3 PAMAM dendrimer was dried *in vacuo* overnight and the resulting solid was weighed (266 mg, 38.6 μmol). The anhydrous DMSO (20.0 mL) was added to this dried G3 PAMAM dendrimer to dissolve completely, and then to this stirred solution was slowly added a solution of ***p*NP-PEG-N$_3$** (415 mg, 0.231 mmol) in DMSO (5.7 mL). The reaction was stirred at room temperature for 33 h under a dry Ar atmosphere. Next, the reaction mixture was dialyzed (Spectra/Por Biotech Regenerated

Cellulose (RC) membrane, MWCO 3500, Spectrum Laboratories) against methanol (× 2, for 2 h each) with stirring to remove DMSO and small molecular reagents such as ***p*NP-PEG-N_3**. After removal of the solvent under reduced pressure, the crude mixture was loaded on a SEC column (Bio-Beads S-X1, H 41 cm × O.D. 4.5 cm) in DMF to isolate the desired **PPL**. The yellowish SEC fractions were combined, concentrated under reduced pressure, and dried *in vacuo* to give 516 mg of **PPL**. See Fig. S12a for ^{1}H NMR peak assignments. ^{1}H NMR (600 MHz, DMSO-d_6) δ 7.22 (br s, 5.03H, NH_{NP} of major isomer), 6.81 (br s, 0.41H, NH_{NP} of minor isomer), 4.04 (t, 9.49H, J = 4.7 Hz, H_{6NP}), 3.63-3.38 (m, H_{1NP}, H_{2NP}, H_{3NP}, H_{4NP}, and H_{5NP}), 3.13-3.00 (m, d, f, f_{NP}, and g_{NP}), 2.65 (s, 116.69H, H_b), 2.57 (s, 47.97H, H_g), 2.43 (m, 55.64H, H_e and H_a), 2.20 (m, 120.00H, H_c); MS (MALDI-TOF, DHB matrix) M_n 15214, M_w 15615, PDI 1.03 (calcd. region: 11041-21296).

Synthesis of PPH

The methanolic solution of G3 PAMAM dendrimer was dried *in vacuo* for 2 h and the resulting solid was weighed (237 mg, 34.4 μmol). The anhydrous DMSO (23.0 mL) was added to this dried G3 PAMAM dendrimer to dissolve completely, and then to this stirred solution was added ***p*NP-PEG-N_3** (617 mg, 344 μmol) in one portion. The reaction was stirred at room temperature for 20 h under a dry Ar atmosphere. Next, the reaction mixture was dialyzed (Spectra/Por Biotech RC membrane, MWCO 3500, Spectrum Laboratories) against methanol (× 2, for 12 h and 2 h each) with stirring to remove DMSO and small molecular reagents such as ***p*NP-PEG-N_3**. After removal of the solvent under reduced pressure, the crude mixture was loaded on a SEC column (Bio-Beads S-X1, H 41.5 cm × O.D. 4.5 cm) in DMF to isolate the desired **PPH**. The yellowish SEC fractions were combined, concentrated under reduced pressure, and dried *in vacuo* to give 390 mg of **PPH**. See Fig. S12b for ^{1}H NMR peak assignments. ^{1}H NMR (600 MHz, CD_3OD) δ 4.16 (m, 21.57H, H_{6NP}), 3.76-3.51 (m, 1540.27H, H_{2NP}, H_{3NP}, H_{4NP}, and H_{5NP}), 3.38 (t, 22.47H, J = 5.0 Hz, H_{1NP}), 3.30-3.21 (m, d, f, f_{NP}, and g_{NP}), 2.80, 2.76 (m, 165.51H, H_b and H_g), 2.61-2.58 (m, 61.81H, H_e and H_a), 2.38 (m, 120.00H, H_c); MS (MALDI-TOF, DHB matrix) M_n 18658, M_w 19848, PDI 1.06 (calcd. region: 10783-29397).

Synthesis of PL_{NH2}

PPL (220 mg, 14.3 μmol) was dissolved completely in DMSO (8.0 mL) with sonication. To this stirred solution was added DIEA (10.0 μL, 57.4 μmol) followed by a solution of succinimidyl 3-(tri-*n*-butylstannyl)benzoate (16.4 mg, 95% purity, 29.5 μmol) in DMSO (1.8 mL) dropwise over a 4-min period. The reaction was stirred at room temperature for 24 h under a dry Ar atmosphere. Subsequently, to this stirred solution was slowly added Cy5.5 NHS ester (5.53 mg, 95% purity, 7.33 μmol) in DMSO-d_6 (400 μL). The reaction was protected from light and continued to stir at room temperature for 77 h under a dry Ar atmosphere. The crude reaction mixture was divided into six equal portions by volume (1.70 mL, each) and five of them were used for consecutive surface modification reactions without purification (*vide infra*). One of six divided portions containing **PL_{NH2}** (1.70 mL, *ca.* 2.39 μmol) was first filtered through a short SEC column (Bio-Beads S-X1, H 5 cm × O.D. 0.7 cm) in DMF, and then purified by a preparative SEC (Bio-Beads S-X1, H 37.5 cm × O.D. 3.0 cm) in DMF. The intense bluish band which eluted first was confirmed to contain the desired product as determined by ^{1}H NMR. The corresponding SEC fractions were combined and dried extensively *in vacuo* to give 44.8 mg of **PL_{NH2}**. See Fig. S13a for ^{1}H NMR peak assignments. ^{1}H NMR (600 MHz, DMSO-d_6) δ 4.03 (m, 10.14H, H_{6NP}), 2.64, 2.57 (m, 141.75H, H_b and H_g), 2.43 (m, 64.85H, H_e and H_a), 2.19 (m, 120.00H, H_c), 0.84 (t, 17.40H, J = 7.2 Hz, H_{1Sn}); MS (MALDI-TOF, DHB matrix) M_n 13858, M_w 14292, PDI 1.03 (calcd. region: 9547-21418).

Synthesis of PL_{12Ac}

To a portion of the crude reaction mixture of **PL_{NH2}** (1.70 mL, *ca.* 2.39 μmol) was slowly added a 0.106 M solution of Ac_2O in DMSO-d_6 (230 μL, 24.3 μmol) followed by DIEA (9.00 μL, 51.7 μmol). The reaction was protected from light and stirred at room temperature for 12 h under a dry Ar atmosphere. In a darkroom, the crude mixture was first filtered through a short SEC column (Bio-Beads S-X1, H 5 cm × O.D. 0.7 cm) in DMF, and then purified by a preparative SEC (Bio-Beads S-X1, H 37.5 cm × O.D. 3.0 cm) in DMF. The intense bluish band which eluted first was confirmed to contain the desired product as determined by ^{1}H NMR. The corresponding SEC fractions were combined and dried extensively *in vacuo* to give 35.3 mg of **PL_{12Ac}**. See Fig. S14a for ^{1}H

NMR peak assignments. ^{1}H NMR (600 MHz, DMSO-d_6) δ 4.03 (m, 9.98H, H_{6NP}), 3.63-3.38 (m, 724.13H, H_{1NP}, H_{2NP}, H_{3NP}, H_{4NP}, and H_{5NP}), 2.65 (m, H_b and H_g), 2.42 (m, 60.00H, H_a and H_e), 2.18 (m, 120.00H, H_c), 1.79 (s, 36.41H, H_{Ac}), 0.84 (t, 16.77H, J = 6.9 Hz, H_{1Sn}); MS (MALDI-TOF, DHB matrix) M_n 13888, M_w 14177, PDI 1.02 (calcd. region: 9761-21045).

Synthesis of PL_{19Ac}

To a portion of the crude reaction mixture of **PL_{NH2}** (1.70 mL, *ca.* 2.39 μmol) was added Ac_2O (17.0 μL, 180 μmol) followed by DMSO-d_6 (200 μL) and DIEA (21.0 μL, 121 μmol). The reaction was protected from light and stirred at room temperature for 12 h under a dry Ar atmosphere. In a darkroom, the crude mixture was first filtered through a short SEC column (Bio-Beads S-X1, H 5 cm × O.D. 0.7 cm) in DMF, and then purified by a preparative SEC (Bio-Beads S-X1, H 35.5 cm × O.D. 3.0 cm) in DMF. The intense bluish band which eluted first was confirmed to contain the desired product as determined by ^{1}H NMR. The corresponding SEC fractions were combined and dried extensively *in vacuo* to give 35.2 mg of **PL_{19Ac}**. See Fig. S15a for ^{1}H NMR peak assignments. ^{1}H NMR (600 MHz, DMSO-d_6) δ 4.03 (m, 9.77H, H_{6NP}), 3.63-3.38 (m, 726.68H, H_{1NP}, H_{2NP}, H_{3NP}, H_{4NP}, and H_{5NP}), 2.65, 2.58 (m, 118.47H, H_b and H_g), 2.42 (m, 60.00H, H_a and H_e), 2.18 (m, 120.00H, H_c), 1.79 (s, 55.96H, H_{Ac}), 0.84 (t, 18.36H, J = 7.0 Hz, H_{1Sn}); MS (MALDI-TOF, DHB matrix) M_n 13411, M_w 13967, PDI 1.04 (calcd. region: 8367-21553).

Synthesis of PL_{SA}

To a portion of the crude reaction mixture of **PL_{NH2}** (1.70 mL, *ca.* 2.39 μmol) was added succinic anhydride (19.6 mg, 196 μmol) followed by DIEA (21.0 μL, 121 μmol). The reaction was protected from light and stirred at room temperature for 60 h under a dry Ar atmosphere. In a darkroom, the crude mixture was first filtered through a short SEC column (Bio-Beads S-X1, H 5 cm × O.D. 0.7 cm) in DMF, and then purified by a preparative SEC (Bio-Beads S-X1, H 38.5 cm × O.D. 3.0 cm) in DMF. The intense bluish band which eluted first was confirmed to contain the desired product as determined by ^{1}H NMR. The corresponding SEC fractions were combined and dried

extensively *in vacuo* to give 37.9 mg of $\mathbf{PL_{SA}}$. See Fig. S16a for ^{1}H NMR peak assignments. ^{1}H NMR (600 MHz, DMSO-d_6) δ 4.03 (m, 10.15H, H_{6NP}), 3.63-3.38 (m, H_{1NP}, H_{2NP}, H_{3NP}, H_{4NP}, and H_{5NP}), 2.65 (m, 105.39H, H_b and H_g), 2.43 (m, 60.00H, H_a and H_e), 2.38 (t, J = 6.1 Hz, H_{1SA}), 2.29 (t, J = 6.3 Hz, H_{2SA}), 2.19 (m, 120.00H, H_c), 0.84 (t, 17.40H, J = 7.3 Hz, H_{1Sn}); MS (MALDI-TOF, DHB matrix) M_n 15426, M_w 15686, PDI 1.02 (calcd. region: 10574-20693).

Synthesis of $\mathbf{PL_{TEG}}$

To a portion of the crude reaction mixture of $\mathbf{PL_{NH2}}$ (1.70 mL, *ca.* 2.39 μmol) was added mTEG-NHS ester (59.7 mg, 179 μmol) in DMSO-d_6 (200 μL) followed by DIEA (21.0 μL, 121 μmol). The reaction was protected from light and stirred at room temperature for 12 h under a dry Ar atmosphere. In a darkroom, the crude mixture was first filtered through a short SEC column (Bio-Beads S-X1, H 5 cm × O.D. 0.7 cm) in DMF, and then purified by a preparative SEC (Bio-Beads S-X1, H 39 cm × O.D. 3.0 cm) in DMF. The intense bluish band which eluted first was confirmed to contain the desired product as determined by ^{1}H NMR. The corresponding SEC fractions were combined and dried extensively *in vacuo* to give 42.0 mg of $\mathbf{PL_{TEG}}$. See Fig. S18a for ^{1}H NMR peak assignments. ^{1}H NMR (600 MHz, DMSO-d_6) δ 4.03 (m, 10.11H, H_{6NP}), 3.63-3.38 (m, 1011.59H, H_{1NP}, H_{2NP}, H_{3NP}, H_{4NP}, H_{5NP}, H_{2TEG}, H_{3TEG}, H_{4TEG}, H_{5TEG}, H_{6TEG}, H_{7TEG}, and H_{8TEG}), 3.23 (s, 68.93H, H_{1TEG}), 2.65 (m, H_b and H_g), 2.42 (m, 63.01H, H_a and H_e), 2.30 (t, 41.92H, J = 6.3 Hz, H_{9TEG}), 2.18 (m, 120.00H, H_c), 0.84 (t, 17.73H, J = 6.9 Hz, H_{1Sn}); MS (MALDI-TOF, DHB matrix) M_n 16888, M_w 17711, PDI 1.05 (calcd. region: 10431-25879).

Synthesis of $\mathbf{PL_{PEG}}$

To a portion of the crude reaction mixture of $\mathbf{PL_{NH2}}$ (1.70 mL, *ca.* 2.39 μmol) was added mPEG-NHS ester (328 mg, 184 μmol) followed by DIEA (21.0 μL, 121 μmol) and DMSO-d_6 (200 μL). The reaction was protected from light and stirred at room temperature for 12 h under a dry Ar atmosphere. In a darkroom, the crude mixture was first filtered through a short SEC column (Bio-Beads S-X1, H 5 cm × O.D. 0.7 cm) in DMF, and then purified by a preparative SEC (Bio-Beads S-X1, H 38.5 cm × O.D. 4.5

cm) in DMF. The intense bluish band which eluted first was confirmed to contain the desired product as determined by ^{1}H NMR. The corresponding SEC fractions were combined and dried extensively *in vacuo* to give 89.9 mg of **PL$_{PEG}$**. See Fig. S19a for ^{1}H NMR peak assignments. ^{1}H NMR (600 MHz, DMSO-d_6) δ 4.03 (m, 9.44H, H_{6NP}), 3.63-3.38 (m, 3616.54H, H_{1NP}, H_{2NP}, H_{3NP}, H_{4NP}, H_{5NP}, H_{2PEG}, H_{3PEG}, and H_{4PEG}), 3.24 (s, 61.93H, H_{1PEG}), 2.64 (m, H_b and H_g), 2.42 (m, H_a and H_e), 2.30 (t, 40.36H, J = 6.2 Hz, H_{5PEG}), 2.18 (m, 120.00H, H_c), 0.84 (t, 17.99H, J = 6.9 Hz, H_{1Sn}); MS (MALDI-TOF, DHB matrix) M_n 40921, M_w 41946, PDI 1.03 (calcd. region: 27620-59976).

Synthesis of PH$_{NH2}$

PPH (379 mg, 15.2 μmol) was dissolved completely in DMSO (8.0 mL) with sonication. To this stirred solution was added DIEA (11.6 μL, 66.6 μmol) followed by a solution of succinimidyl 3-(tri-*n*-butylstannyl)benzoate (15.9 mg, 95% purity, 28.6 μmol) in DMSO (3.0 mL) dropwise over a 7-min period. The reaction was stirred at room temperature for 25 h under a dry Ar atmosphere. Subsequently, to this stirred solution was slowly added Cy5.5 NHS ester (6.28 mg, 95% purity, 8.33 μmol) in DMSO-d_6 (300 μL). The reaction was protected from light and continued to stir at room temperature for 28 h under a dry Ar atmosphere. The crude reaction mixture was divided into six portions by volume and three of them were used for consecutive surface modification reactions without purification (*vide infra*). One of six divided portions containing **PH$_{NH2}$** (1.74 mL, *ca.* 2.34 μmol) was first filtered through a short SEC column (Bio-Beads S-X1, H 5 cm × O.D. 0.7 cm) in DMF, and then purified by a preparative SEC (Bio-Beads S-X1, H 37 cm × O.D. 3.0 cm) in DMF. The intense bluish band which eluted first was confirmed to contain the desired product as determined by ^{1}H NMR. The corresponding SEC fractions were combined and dried extensively *in vacuo* to give 67.4 mg of **PH$_{NH2}$**. See Fig. S20a for ^{1}H NMR peak assignments. ^{1}H NMR (600 MHz, DMSO-d_6) δ 7.21 (br s, 10.98H, NH_{NP} of major isomer), 6.81 (br s, 0.77H, NH_{NP} of minor isomer), 4.03 (s, 21.96H, H_{6NP}), 2.65, 2.56 (m, 161.32H, H_b and H_g), 2.43 (m, 59.78H, H_e and H_a), 2.19 (m, 120.00H, H_c), 0.84 (t, 14.32H, J = 6.6 Hz, H_{1Sn}); MS (MALDI-TOF, DHB matrix) M_n 19790, M_w 20302, PDI 1.03 (calcd. region: 12710-28720).

Synthesis of PH_{SA}

To a portion of the crude reaction mixture of $\mathbf{PH_{NH2}}$ (1.74 mL, *ca.* 2.34 μmol) was added succinic anhydride (15.4 mg, 153 μmol) followed by DIEA (18.0 μL, 103 μmol). The reaction was protected from light and stirred at room temperature for 48 h under a dry Ar atmosphere. In a darkroom, the crude mixture was first filtered through a short SEC column (Bio-Beads S-X1, H 5 cm × O.D. 0.7 cm) in DMF, and then purified by a preparative SEC (Bio-Beads S-X1, H 39 cm × O.D. 3.0 cm) in DMF. The intense bluish band which eluted first was confirmed to contain the desired product as determined by ^{1}H NMR. The corresponding SEC fractions were combined and dried extensively *in vacuo* to give 61.7 mg of $\mathbf{PH_{SA}}$. See Fig. S21a for ^{1}H NMR peak assignments. ^{1}H NMR (600 MHz, DMSO-d_6) δ 7.19 (br s, 11.29H, NH_{NP} of major isomer), 6.78 (br s, 1.16H, NH_{NP} of minor isomer), 4.03 (m, 24.17H, H_{6NP}), 3.63-3.38 (m, 1664.93H, H_{1NP}, H_{2NP}, H_{3NP}, H_{4NP}, and H_{5NP}), 2.65 (m, H_b and H_g), 2.43 (m, 60.00H, H_e and H_a), 2.40 (t, J = 6.7 Hz, H_{1SA}), 2.29 (t, J = 6.6 Hz, H_{2SA}) 2.19 (m, 120.00H, H_c), 0.84 (t, 16.35H, J = 7.3 Hz, H_{1Sn}); MS (MALDI-TOF, DHB matrix) M_n 21796, M_w 22785, PDI 1.05 (calcd. region: 13615-32728).

Synthesis of PH_{TEG}

To a portion of the crude reaction mixture of $\mathbf{PH_{NH2}}$ (2.60 mL, *ca.* 3.50 μmol) was added mTEG-NHS ester (180 mg, 539 μmol) in DMSO-d_6 (180 μL) followed by DIEA (26.7 μL, 154 μmol). The reaction was protected from light and stirred at room temperature for 48 h under a dry Ar atmosphere. In a darkroom, the crude mixture was first filtered through a short SEC column (Bio-Beads S-X1, H 5 cm × O.D. 0.7 cm) in DMF, and then purified by a preparative SEC (Bio-Beads S-X1, H 40.5 cm × O.D. 4.5 cm) in DMF. The intense bluish band which eluted first was confirmed to contain the desired product as determined by ^{1}H NMR. The corresponding SEC fractions were combined and dried extensively *in vacuo* to give 102 mg of $\mathbf{PH_{TEG}}$. See Fig. S22a for ^{1}H NMR peak assignments. ^{1}H NMR (600 MHz, DMSO-d_6) δ 7.18 (br s, 10.81H, NH_{NP} of major isomer), 6.76 (br s, 1.27H, NH_{NP} of minor isomer), 4.03 (m, 22.42H, H_{6NP}), 3.62-3.38 (m, 1741.89H, H_{1NP}, H_{2NP}, H_{3NP}, H_{4NP}, H_{5NP}, H_{2TEG}, H_{3TEG}, H_{4TEG}, H_{5TEG}, H_{6TEG}, H_{7TEG}, and H_{8TEG}), 3.23 (s, 54.78H, H_{1TEG}), 2.65 (m, H_b and H_g), 2.42 (m, 60.00H, H_e and

H_a), 2.30 (t, 34.55H, J = 6.3 Hz, H_{9TEG}), 2.18 (m, 120.00H, H_c), 0.84 (t, 14.32H, J = 7.2 Hz, H_{1Sn}); MS (MALDI-TOF, DHB matrix) M_n 22545, M_w 23275, PDI 1.03 (calcd. region: 14789-33369).

Synthesis of PH_{PEG}

To a portion of the crude reaction mixture of $\mathbf{PH_{NH2}}$ (1.74 mL, *ca.* 2.34 μmol) was added mPEG-NHS ester (182 mg, 102 μmol) followed by DIEA (18.0 μL, 103 μmol). The reaction was protected from light and stirred at room temperature for 48 h under a dry Ar atmosphere. In a darkroom, the crude mixture was first filtered through a short SEC column (Bio-Beads S-X1, H 5 cm × O.D. 0.7 cm) in DMF, and then purified by a preparative SEC (Bio-Beads S-X1, H 41 cm × O.D. 4.5 cm) in DMF. The intense bluish band which eluted first was confirmed to contain the desired product as determined by ^{1}H NMR. The corresponding SEC fractions were combined and dried extensively *in vacuo* to give 107 mg of $\mathbf{PH_{PEG}}$. See Fig. S23a for ^{1}H NMR peak assignments. ^{1}H NMR (600 MHz, DMSO-d_6) δ 7.18 (br s, 12.08H, NH_{NP} of major isomer), 6.76 (br s, 2.22H, NH_{NP} of minor isomer), 4.03, 3.63-3.38 (m, 3860.42H, H_{1NP}, H_{2NP}, H_{3NP}, H_{4NP}, H_{5NP}, H_{6NP}, H_{2PEG}, H_{3PEG}, and H_{4PEG}), 3.24 (s, 49.66H, H_{1PEG}), 2.65 (m, H_b and H_g), 2.42 (m, 58.50H, H_e and H_a), 2.30 (t, 31.65H, J = 6.0 Hz, H_{5PEG}), 2.18 (m, 120.00H, H_c), 0.84 (t, 15.65H, J = 7.2 Hz, H_{1Sn}); MS (MALDI-TOF, DHB matrix) M_n 41295, M_w 42156, PDI 1.02 (calcd. region: 28790-58923).

Synthesis of L_{NH2}

To a solution of $\mathbf{PL_{NH2}}$ (35.3 mg, 2.16 μmol) in DMSO-d_6 (1.24 mL) was added a solution of **DBCO-c(RGDfK)** (20.7 mg, 17.5 μmol) in DMSO-d_6 (160 μL). The reaction was protected from light and stirred at room temperature for 24 h under a dry Ar atmosphere. In a darkroom, the crude mixture was purified by a preparative SEC (Bio-Beads S-X1, H 39.5 cm × O.D. 3.0 cm) in DMF. The intense bluish band which eluted first was confirmed to contain the desired product as determined by ^{1}H NMR. The corresponding SEC fractions were combined and dried extensively *in vacuo* to give $\mathbf{L_{NH2}'}$ (structure not shown). Unfortunately, the analysis of $\mathbf{L_{NH2}'}$ by ^{1}H NMR in DMSO-d_6 indicated that only *ca.* 3.5 RGD units were attached by cycloaddition reaction out of

approximately five available azide groups of $\mathbf{PL_{NH2}}$. In an effort to bring the RGD attachment to completion, another round of cycloaddition (*i.e.*, click) reaction was pursued by adding freshly made **DBCO-c(RGDfK)** (14.8 mg, 12.5 μmol) to the solution of $\mathbf{L_{NH2}'}$ (*ca.* 2.16 μmol) in DMSO-d_6 (1.4 mL). The reaction was protected from light and stirred at room temperature for 24 h under a dry Ar atmosphere. In a darkroom, the crude mixture was purified by the preparative SEC using three different columns consecutively: Bio-Beads S-X1 (H 31 cm × O.D. 3.0 cm) in DMF, Sephadex LH-20 (H 37 cm × O.D. 3.0 cm) in methanol, and Sephadex G-25 (H 37 cm × O.D. 4.5 cm) in deionized water. The SEC fractions containing the intense bluish band which eluted first were combined and dried extensively *in vacuo* to give 16.6 mg of $\mathbf{L_{NH2}}$. See Fig. S13b for ^{1}H NMR peak assignments. ^{1}H NMR (600 MHz, DMSO-d_6) δ 5.90 (d, 1.86H, J = 18.2 Hz, $H_{9'(A)}$), 5.84 (d, 2.14H, J = 16.7 Hz, $H_{9'(B)}$), 5.52 (d, 0.64H, J = 19.7 Hz, $H_{9'(C)}$), 4.95 (d, 0.26H, J = 18.9 Hz, $H_{9'(C)}$), 4.47 (d, J = 16.7 Hz, $H_{9'(A)}$), 4.46 (d, J = 16.1 Hz, $H_{9'(B)}$), 0.84 (br s, 17.40H, H_{1Sn}); MS (MALDI-TOF, DHB matrix) M_n 24549, M_w 25697, PDI 1.05 (calcd. region: 15689-35085).

Synthesis of $\mathbf{L_{12Ac}}$

To a solution of $\mathbf{PL_{12Ac}}$ (31.2 mg, 1.86 μmol) in DMSO-d_6 (1.07 mL) was added a solution of **DBCO-c(RGDfK)** (16.9 mg, 14.3 μmol) in DMSO-d_6 (130 μL). The reaction was protected from light and stirred at room temperature for 24 h under a dry Ar atmosphere. In a darkroom, the crude mixture was purified by a preparative SEC (Bio-Beads S-X1, H 36 cm × O.D. 3.0 cm) in DMF. The intense bluish band which eluted first was confirmed to contain the desired product as determined by ^{1}H NMR. The corresponding SEC fractions were combined and dried extensively *in vacuo* to give $\mathbf{L_{12Ac}'}$ (structure not shown). Unfortunately, the analysis of $\mathbf{L_{12Ac}'}$ by ^{1}H NMR in DMSO-d_6 indicated that only *ca.* 3.4 RGD units were attached by cycloaddition reaction out of approximately five available azide groups of $\mathbf{PL_{12Ac}}$. In an effort to bring the RGD attachment to completion, another round of cycloaddition (*i.e.*, click) reaction was pursued by adding freshly made **DBCO-c(RGDfK)** (21.6 mg, 18.2 μmol) to the solution of $\mathbf{L_{12Ac}'}$ (*ca.* 1.86 μmol) in DMSO-d_6 (1.2 mL). The reaction was protected from light and stirred at room temperature for 44 h under a dry Ar atmosphere. In a darkroom, the

crude mixture was purified by the preparative SEC first using Bio-Beads S-X1 (H 32.5 cm × O.D. 3.0 cm) in DMF and then using Sephadex LH-20 (H 37.5 cm × O.D. 3.0 cm) in methanol. The SEC fractions containing the intense bluish band which eluted first were combined and dried extensively *in vacuo* to give 31.7 mg of $\mathbf{L_{12Ac}}$. See Fig. S14b for ^{1}H NMR peak assignments. ^{1}H NMR (600 MHz, DMSO-d_6) δ 5.90 (d, 1.76H, J = 17.5 Hz, $H_{9'(A)}$), 5.84 (d, 2.02H, J = 16.9 Hz, $H_{9'(B)}$), 5.52 (d, 0.24H, J = 19.2 Hz, $H_{9'(C)}$), 4.95 (d, 0.20H, J = 20.7 Hz, $H_{9'(C)}$), 4.47 (d, J = 18.0 Hz, $H_{9'(A)}$), 4.46 (d, J = 16.9 Hz, $H_{9'(B)}$), 1.79 (s, 35.79H, H_{Ac}), 0.84 (t, 16.77 H, J = 6.1 Hz, H_{1Sn}); MS (MALDI-TOF, DHB matrix) M_n 20286, M_w 21159, PDI 1.04 (calcd. region: 12879-29112).

Synthesis of $\mathbf{L_{19Ac}}$

To a solution of $\mathbf{PL_{19Ac}}$ (30.6 mg, 1.79 μmol) in DMSO-d_6 (1.07 mL) was added a solution of **DBCO-c(RGDfK)** (16.9 mg, 14.3 μmol) in DMSO-d_6 (130 μL). The reaction was protected from light and stirred at room temperature for 24 h under a dry Ar atmosphere. In a darkroom, the crude mixture was purified by a preparative SEC (Bio-Beads S-X1, H 38 cm × O.D. 3.0 cm) in DMF. The intense bluish band which eluted first was confirmed to contain the desired product as determined by ^{1}H NMR. The corresponding SEC fractions were combined and dried extensively *in* v*acuo* to give $\mathbf{L_{19Ac}'}$ (structure not shown). Unfortunately, the analysis of $\mathbf{L_{19Ac}'}$ by ^{1}H NMR in DMSO-d_6 indicated that only *ca.* 3.6 RGD units were attached by cycloaddition reaction out of approximately five available azide groups of $\mathbf{PL_{19Ac}}$. In an effort to bring the RGD attachment to completion, another round of cycloaddition (*i.e.*, click) reaction was pursued by adding freshly made **DBCO-c(RGDfK)** (10.5 mg, 8.86 μmol) to the solution of $\mathbf{L_{19Ac}'}$ (*ca.* 1.79 μmol) in DMSO-d_6 (1.2 mL). The reaction was protected from light and stirred at room temperature for 67 h under a dry Ar atmosphere. In a darkroom, the crude mixture was purified by the preparative SEC first using Bio-Beads S-X1 (H 39.5 cm × O.D. 3.0 cm) in DMF and then using Sephadex LH-20 (H 36.5 cm × O.D. 3.0 cm) in methanol. The SEC fractions containing the intense bluish band which eluted first were combined and dried extensively *in vacuo* to give 34.3 mg of $\mathbf{L_{19Ac}}$. See Fig. S15b for ^{1}H NMR peak assignments. ^{1}H NMR (600 MHz, DMSO-d_6) δ 5.91 (d, 1.92H, J = 17.9 Hz, $H_{9'(A)}$), 5.84 (d, 2.09H, J = 16.7 Hz, $H_{9'(B)}$), 5.52 (d, 0.17H, J = 18.3 Hz, $H_{9'(C)}$), 4.95 (d,

0.22H, J = 18.8 Hz, $H_{9'(C)}$), 4.47 (d, J = 17.1 Hz, $H_{9'(A)}$), 4.46 (d, J = 16.7 Hz, $H_{9'(B)}$), 1.79 (s, 59.72H, H_{Ac}), 0.84 (t, 18.36 H, J = 6.8 Hz, H_{1Sn}); MS (MALDI-TOF, DHB matrix) M_n 18994, M_w 20065, PDI 1.06 (calcd. region: 11313-28487).

Synthesis of L_{SA}

To a solution of **PL_{SA}** (33.8 mg, 1.85 μmol) in DMSO-d_6 (1.06 mL) was added a solution of **DBCO-c(RGDfK)** (18.2 mg, 15.4 μmol) in DMSO-d_6 (140 μL). The reaction was protected from light and stirred at room temperature for 24 h under a dry Ar atmosphere. In a darkroom, the crude mixture was purified by a preparative SEC (Bio-Beads S-X1, H 38.5 cm × O.D. 3.0 cm) in DMF. The intense bluish band which eluted first was confirmed to contain the desired product as determined by ^{1}H NMR. The corresponding SEC fractions were combined and dried extensively *in vacuo* to give **L_{SA}'** (structure not shown). Unfortunately, the analysis of **L_{SA}'** by ^{1}H NMR in DMSO-d_6 indicated that only *ca.* 3.4 RGD units were attached by cycloaddition reaction out of approximately five available azide groups of **PL_{SA}**. In an effort to bring the RGD attachment to completion, another round of cycloaddition (*i.e.*, click) reaction was pursued by adding freshly made **DBCO-c(RGDfK)** (10.5 mg, 8.88 μmol) to the solution of **L_{SA}'** (*ca.* 1.85 μmol) in DMSO-d_6 (1.2 mL). The reaction was protected from light and stirred at room temperature for 43 h under a dry Ar atmosphere. In a darkroom, the crude mixture was purified by the preparative SEC first using Bio-Beads S-X1 (H 38 cm × O.D. 3.0 cm) in DMF and then using Sephadex LH-20 (H 37 cm × O.D. 3.0 cm) in methanol. The SEC fractions containing the intense bluish band which eluted first were combined and dried extensively *in vacuo* to give 31.7 mg of **L_{SA}**. See Fig. S16b and S17 for ^{1}H NMR peak assignments. ^{1}H NMR (600 MHz, DMSO-d_6) δ 5.91 (d, 2.15H, J = 17.1 Hz, $H_{9'(A)}$), 5.84 (d, 2.26H, J = 16.4 Hz, $H_{9'(B)}$), 5.52 (d, 0.62H, J = 18.9 Hz, $H_{9'(C)}$), 4.95 (d, 0.48H, J = 18.9 Hz, $H_{9'(C)}$), 4.47 (d, J = 17.8 Hz, $H_{9'(A)}$), 4.46 (d, J = 16.4 Hz, $H_{9'(B)}$), 0.84 (t, 17.40H, J = 7.2 Hz, H_{1Sn}); MS (MALDI-TOF, DHB matrix) M_n 20772, M_w 22092, PDI 1.06 (calcd. region: 11386-32665).

Synthesis of L_{TEG}

To a solution of **PL_{TEG}** (35.6 mg, 1.70 μmol) in DMSO-d_6 (0.98 mL) was added a

solution of **DBCO-c(RGDfK)** (15.6 mg, 13.2 μmol) in DMSO-d_6 (120 μL). The reaction was protected from light and stirred at room temperature for 24 h under a dry Ar atmosphere. In a darkroom, the crude mixture was purified by a preparative SEC (Bio-Beads S-X1, H 38.5 cm × O.D. 3.0 cm) in DMF. The intense bluish band which eluted first was confirmed to contain the desired product as determined by ^{1}H NMR. The corresponding SEC fractions were combined and dried extensively *in* v*acuo* to give **L_{TEG}′** (structure not shown). Unfortunately, the analysis of **L_{TEG}′** by ^{1}H NMR in DMSO-d_6 indicated that only *ca.* 2.8 RGD units were attached by cycloaddition reaction out of approximately five available azide groups of **PL_{TEG}**. In an effort to bring the RGD attachment to completion, another round of cycloaddition (*i.e.*, click) reaction was pursued by adding freshly made **DBCO-c(RGDfK)** (12.8 mg, 10.8 μmol) to the solution of **L_{TEG}′** (*ca.* 1.70 μmol) in DMSO-d_6 (1.1 mL). The reaction was protected from light and stirred at room temperature for 24 h under a dry Ar atmosphere. In a darkroom, the crude mixture was purified by the preparative SEC first using Bio-Beads S-X1 (H 31.5 cm × O.D. 3.0 cm) in DMF and then using Sephadex LH-20 (H 38 cm × O.D. 3.0 cm) in methanol. The SEC fractions containing the intense bluish band which eluted first were combined and dried extensively *in vacuo* to give 38.2 mg of **L_{TEG}**. See Fig. S18b for ^{1}H NMR peak assignments. ^{1}H NMR (600 MHz, DMSO-d_6) δ 5.91 (d, 2.07H, J = 17.1 Hz, $H_{9'(A)}$), 5.84 (d, 2.09H, J = 16.5 Hz, $H_{9'(B)}$), 5.52 (d, 0.37H, J = 18.1 Hz, $H_{9'(C)}$), 4.95 (d, 0.34H, J = 19.3 Hz, $H_{9'(C)}$), 4.47 (d, J = 17.5 Hz, $H_{9'(A)}$), 4.46 (d, J = 16.7 Hz, $H_{9'(B)}$), 0.84 (t, 17.73H, J = 7.3 Hz, H_{1Sn}); MS (MALDI-TOF, DHB matrix) M_n 21848, M_w 23172, PDI 1.06 (calcd. region: 12410-33816).

Synthesis of L_{PEG}

To a solution of **PL_{PEG}** (85.6 mg, 1.73 μmol) in DMSO-d_6 (0.88 mL) was added a solution of **DBCO-c(RGDfK)** (15.6 mg, 13.2 μmol) in DMSO-d_6 (120 μL). The reaction was protected from light and stirred at room temperature for 24 h under a dry Ar atmosphere. In a darkroom, the crude mixture was purified by a preparative SEC (Bio-Beads S-X1, H 40 cm × O.D. 4.5 cm) in DMF. The intense bluish band which eluted first was confirmed to contain the desired product as determined by ^{1}H NMR. The corresponding SEC fractions were combined and dried extensively *in* v*acuo* to give

$\mathbf{L_{PEG}'}$ (structure not shown). Unfortunately, the analysis of $\mathbf{L_{PEG}'}$ by ^{1}H NMR in DMSO-d_6 indicated that only *ca.* 2.9 RGD units were attached by cycloaddition reaction out of approximately five available azide groups of $\mathbf{PL_{PEG}}$. In an effort to bring the RGD attachment to completion, another round of cycloaddition (*i.e.*, click) reaction was pursued by adding freshly made **DBCO-c(RGDfK)** (11.7 mg, 9.87 μmol) to the solution of $\mathbf{L_{PEG}'}$ (*ca.* 1.73 μmol) in DMSO-d_6 (1.0 mL). The reaction was protected from light and stirred at room temperature for 67 h under a dry Ar atmosphere. In a darkroom, the crude mixture was purified by the preparative SEC first using Bio-Beads S-X1 (H 38.5 cm × O.D. 4.5 cm) in DMF and then using Sephadex LH-20 (H 30 cm × O.D. 4.5 cm) in methanol. The SEC fractions containing the intense bluish band which eluted first were combined and dried extensively *in vacuo* to give 86.2 mg of $\mathbf{L_{PEG}}$. See Fig. S19b for ^{1}H NMR peak assignments. ^{1}H NMR (600 MHz, DMSO-d_6) δ 5.91 (d, 1.95H, J = 16.9 Hz, $H_{9'(A)}$), 5.85 (d, 1.69H, J = 15.9 Hz, $H_{9'(B)}$), 5.53 (d, 0.49H, J = 19.2 Hz, $H_{9'(C)}$), 5.00-4.93 (m, 0.76H, $H_{9'(C)}$), 4.47 (d, J = 18.1 Hz, $H_{9'(A)}$), 4.46 (d, J = 17.0 Hz, $H_{9'(B)}$), 0.84 (t, 17.99H, J = 7.4 Hz, H_{1Sn}); MS (MALDI-TOF, DHB matrix) M_n 45533, M_w 47176, PDI 1.04 (calcd. region: 29427-65644).

Synthesis of $\mathbf{H_{NH2}}$

To a solution of $\mathbf{PH_{NH2}}$ (53.7 mg, 2.12 μmol) in DMSO-d_6 (1.20 mL) was added a solution of **DBCO-c(RGDfK)** (31.4 mg, 26.5 μmol) in DMSO-d_6 (160 μL). The reaction was protected from light and stirred at room temperature for 42 h under a dry Ar atmosphere. In a darkroom, the crude mixture was purified by a preparative SEC (Bio-Beads S-X1, H 37.5 cm × O.D. 3.0 cm) in DMF. The intense bluish band which eluted first was confirmed to contain the desired product as determined by ^{1}H NMR. The corresponding SEC fractions were combined and dried extensively *in vacuo* to give $\mathbf{H_{NH2}'}$ (structure not shown). Unfortunately, the analysis of $\mathbf{H_{NH2}'}$ by ^{1}H NMR in DMSO-d_6 indicated that only *ca.* 4.2 RGD units were attached by cycloaddition reaction out of *ca.* 11 available azide groups of $\mathbf{PH_{NH2}}$. In an effort to bring the RGD attachment to completion, another round of cycloaddition (*i.e.*, click) reaction was pursued by adding a solution of freshly made of **DBCO-c(RGDfK)** (34.0 mg, 28.8 μmol) in DMSO-d_6 (580 μL) to the solution of $\mathbf{H_{NH2}'}$ (*ca.* 2.12 μmol) in DMSO-d_6 (800 μL). The reaction was

protected from light and stirred at room temperature for 24 h under a dry Ar atmosphere. In a darkroom, the crude mixture was purified by the preparative SEC first using Bio-Beads S-X1 (H 33.5 cm × O.D. 3.0 cm) in DMF and then using Sephadex LH-20 (H 39.5 cm × O.D. 3.0 cm) in methanol. The SEC fractions containing the intense bluish band which eluted first were combined and dried extensively *in vacuo* to give 48.6 mg of $\mathbf{H_{NH2}}$. See Fig. S20b for ^{1}H NMR peak assignments. ^{1}H NMR (600 MHz, DMSO-d_6) δ 5.90 (d, 4.03H, J = 17.5 Hz, $H_{9'(A)}$), 5.84 (d, 5.17H, J = 16.2 Hz, $H_{9'(B)}$), 5.52 (d, 1.06H, J = 19.8 Hz, $H_{9'(C)}$), 4.95 (d, 1.25H, J = 17.2 Hz, $H_{9'(C)}$), 4.47 (d, J = 17.3 Hz, $H_{9'(A)}$), 4.46 (d, J = 16.4 Hz, $H_{9'(B)}$), 0.84 (t, 14.32 H, J = 6.5 Hz, H_{1Sn}); MS (MALDI-TOF, DHB matrix) M_n 31426, M_w 33447, PDI 1.06 (calcd. region: 17613-48150).

Synthesis of $\mathbf{H_{SA}}$

To a solution of $\mathbf{PH_{SA}}$ (51.2 mg, 1.81 μmol) in DMSO-d_6 (1.10 mL) was added a solution of **DBCO-c(RGDfK)** (29.4 mg, 24.9 μmol) in DMSO-d_6 (150 μL). The reaction was protected from light and stirred at room temperature for 42 h under a dry Ar atmosphere. In a darkroom, the crude mixture was purified by a preparative SEC (Bio-Beads S-X1, H 39 cm × O.D. 3.0 cm) in DMF. The intense bluish band which eluted first was confirmed to contain the desired product as determined by ^{1}H NMR. The corresponding SEC fractions were combined and dried extensively *in* v*acuo* to give $\mathbf{H_{SA}'}$ (structure not shown). Unfortunately, the analysis of $\mathbf{H_{SA}'}$ by ^{1}H NMR in DMSO-d_6 indicated that only *ca.* 4.1 RGD units were attached by cycloaddition reaction out of *ca.* 11 available azide groups of $\mathbf{PH_{SA}}$. In an effort to bring the RGD attachment to completion, another round of cycloaddition (*i.e.*, click) reaction was pursued by adding a solution of freshly made **DBCO-c(RGDfK)** (31.7 mg, 26.8 μmol) in DMSO-d_6 (0.50 mL) to the solution of $\mathbf{H_{SA}'}$ (*ca.* 1.81 μmol) in DMSO-d_6 (0.80 mL). The reaction was protected from light and stirred at room temperature for 24 h under a dry Ar atmosphere. In a darkroom, the crude mixture was purified by the preparative SEC first using Bio-Beads S-X1 (H 32.5 cm × O.D. 3.0 cm) in DMF and then using Sephadex LH-20 (H 39 cm × O.D. 3.0 cm) in methanol. The SEC fractions containing the intense bluish band which eluted first were combined and dried extensively *in vacuo* to give 68.3 mg of $\mathbf{H_{SA}}$. See Fig. S21b for ^{1}H NMR peak assignments. ^{1}H NMR (600 MHz, DMSO-d_6) δ 5.91 (d,

4.36H, J = 17.0 Hz, $H_{9'(A)}$), 5.84 (d, 4.79H, J = 16.9 Hz, $H_{9'(B)}$),), 5.52 (d, 0.99H, J = 18.9 Hz, $H_{9'(C)}$), 4.95 (d, 1.01H, J = 19.3 Hz, $H_{9'(C)}$), 4.47 (d, J = 17.6 Hz, $H_{9'(A)}$), 4.46 (d, J = 17.1 Hz, $H_{9'(B)}$), 0.84 (t, 16.35H, J = 7.1 Hz, H_{1Sn}); MS (MALDI-TOF, DHB matrix) M_n 30584, M_w 33414, PDI 1.09 (calcd. region: 14638-49553).

Synthesis of $\mathbf{H_{TEG}}$

To a solution of **$\mathbf{PH_{TEG}}$** (92.9 mg, 3.16 μmol) in DMSO-d_6 (1.80 mL) was added a solution of **DBCO-c(RGDfK)** (47.0 mg, 39.8 μmol) in DMSO-d_6 (240 μL). The reaction was protected from light and stirred at room temperature for 42 h under a dry Ar atmosphere. In a darkroom, the crude mixture was purified by a preparative SEC (Bio-Beads S-X1, H 41.5 cm × O.D. 4.5 cm) in DMF. The intense bluish band which eluted first was confirmed to contain the desired product as determined by ^{1}H NMR. The corresponding SEC fractions were combined and dried extensively *in vacuo* to give **$\mathbf{H_{TEG}'}$** (structure not shown). Unfortunately, the analysis of **$\mathbf{H_{TEG}'}$** by ^{1}H NMR in DMSO-d_6 indicated that only *ca.* 3.9 RGD units were attached by cycloaddition reaction out of *ca.* 11 available azide groups of **$\mathbf{PH_{TEG}}$**. In an effort to bring the RGD attachment to completion, another round of cycloaddition (*i.e.*, click) reaction was pursued by adding a solution of freshly made **DBCO-c(RGDfK)** (35.9 mg, 30.4 μmol) in DMSO-d_6 (600 μL) to the solution of **$\mathbf{H_{TEG}'}$** (*ca.* 3.16 μmol) in DMSO-d_6 (1.5 mL). The reaction was protected from light and stirred at room temperature for 48 h under a dry Ar atmosphere. In a darkroom, the crude mixture was purified by the preparative SEC first using Bio-Beads S-X1 (H 31.5 cm × O.D. 4.5 cm) in DMF and then using Sephadex LH-20 (H 38.5 cm × O.D. 4.5 cm) in methanol. The SEC fractions containing the intense bluish band which eluted first were combined and dried extensively *in vacuo* to give 99.7 mg of **$\mathbf{H_{TEG}}$**. See Fig. S22b for ^{1}H NMR peak assignments. ^{1}H NMR (600 MHz, DMSO-d_6) δ 5.91 (d, 4.10H, J = 17.5 Hz, $H_{9'(A)}$), 5.84 (d, 4.93H, J = 16.6 Hz, $H_{9'(B)}$), 5.52 (d, 1.26H, J = 18.6 Hz, $H_{9'(C)}$), 4.95 (d, 1.08H, J = 19.8 Hz, $H_{9'(C)}$), 4.47 (d, J = 17.2 Hz, $H_{9'(A)}$), 4.46 (d, J = 16.6 Hz, $H_{9'(B)}$), 0.84 (t, 14.32H, J = 6.9 Hz, H_{1Sn}); MS (MALDI-TOF, DHB matrix) M_n 29445, M_w 31082, PDI 1.06 (calcd. region: 17532-46096).

Synthesis of H_{PEG}

To a solution of **PH_{PEG}** (81.4 mg, 1.52 μmol) in DMSO-d_6 (1.10 mL) was added a solution of **DBCO-c(RGDfK)** (29.4 mg, 24.9 μmol) in DMSO-d_6 (150 μL). The reaction was protected from light and stirred at room temperature for 42 h under a dry Ar atmosphere. In a darkroom, the crude mixture was purified by a preparative SEC (Bio-Beads S-X1, H 41.5 cm × O.D. 4.5 cm) in DMF. The intense bluish band which eluted first was confirmed to contain the desired product as determined by ^{1}H NMR. The corresponding SEC fractions were combined and dried extensively *in vacuo* to give **H_{PEG}'** (structure not shown). Unfortunately, the analysis of **H_{PEG}'** by ^{1}H NMR in DMSO-d_6 indicated that only *ca.* 6.9 RGD units were attached by cycloaddition reaction out of *ca.* 12 available azide groups of **PH_{PEG}**. In an effort to bring the RGD attachment to completion, another round of cycloaddition (*i.e.*, click) reaction was pursued by adding a solution of freshly made **DBCO-c(RGDfK)** (21.7 mg, 18.3 μmol) in DMSO-d_6 (370 μL) to the solution of **H_{PEG}'** (*ca.* 1.52 μmol) in DMSO-d_6 (900 μL). The reaction was protected from light and stirred at room temperature for 48 h under a dry Ar atmosphere. In a darkroom, the crude mixture was purified by the preparative SEC first using Bio-Beads S-X1 (H 39.5 cm × O.D. 4.5 cm) in DMF and then using Sephadex LH-20 (H 39 cm × O.D. 4.5 cm) in methanol. The SEC fractions containing the intense bluish band which eluted first were combined and dried extensively *in vacuo* to give 90.2 mg of **H_{PEG}**. See Fig. S23b for ^{1}H NMR peak assignments. ^{1}H NMR (600 MHz, DMSO-d_6) δ 5.91 (d, 4.47H, J = 16.7 Hz, $H_{9'(A)}$), 5.84 (d, 5.33H, J = 16.4 Hz, $H_{9'(B)}$), 5.52 (d, 0.90H, J = 19.6 Hz, $H_{9'(C)}$), 4.95 (d, 0.90H, J = 19.4 Hz, $H_{9'(C)}$), 4.47 (d, J = 17.6 Hz, $H_{9'(A)}$), 4.46 (d, J = 17.3 Hz, $H_{9'(B)}$), 0.84 (t, 15.65H, J = 7.3 Hz, H_{1Sn}); MS (MALDI-TOF, DHB matrix) M_n 50296, M_w 52053, PDI 1.03 (calcd. region: 33056-69945).

Synthesis of G3-32PEG

The methanolic solution of G3 PAMAM dendrimer was dried *in vacuo* overnight and the resulting solid was weighed (1.14 mg, 0.16 μmol). The anhydrous DMSO (110 μL) was added to this dried G3 PAMAM dendrimer to dissolve completely, and then to this stirred solution was slowly added DIEA (10.0 μL, 57.4 μmol) and mPEG-NHS ester (10.7 mg, 5.99 μmol). The reaction was stirred at room temperature for 44 h under a dry

Ar atmosphere. Next, the crude mixture was loaded on a SEC column (Bio-Beads S-X1, H 37.5 cm × O.D. 3.0 cm) in DMF to isolate the desired **G3-32PEG**. The SEC fractions containing the dendrimer conjugate were combined, concentrated under reduced pressure, and dried *in vacuo* to give 5.52 mg of **G3-32PEG**. For ^{1}H NMR peak assignments, see Fig. S31c for the labeling method. ^{1}H NMR (600 MHz, DMSO-d_6) δ 7.90 (s, NH_{G3}), 7.87 (s, NH_{PEG}), 7.78 (br s, 79.04H (for all three types of NH peaks including those at 7.90 and 7.87 ppm), NH_{G0}, NH_{G1}, and NH_{G2}), 3.63-3.38 (m, 4184.34H, H_{2PEG}, H_{3PEG}, and H_{4PEG}), 3.24 (s, 86.85H, H_{1PEG}), 3.07 (s, 183.35H, H_d, H_{fPEG} and H_{gPEG}), 2.65 (m, H_b), 2.42 (m, H_e and H_a), 2.30 (t, 64.17H, J = 6.5 Hz, H_{5PEG}), 2.18 (m, 120.00H, H_c); MS (MALDI-TOF, DHB matrix) M_n 47281, M_w 48795, PDI 1.03 (calcd. region: 31944-66492).

Cell Culture

The human malignant glioblastoma U87MG cell line was obtained from American Type Culture Collection (ATCC) and cultured in minimum essential medium (MEM; Welgene or Gibco) supplemented with 10% fetal bovine serum (FBS, Gibco) and antibiotic–antimycotic (100 U/mL penicillin, 100 μg/mL streptomycin, and 0.25 μg/mL Fungizone® (amphotericin B); Gibco) at 37 °C in a humidified atmosphere containing 5% carbon dioxide (CO_2).

Cytotoxicity Assays

Stock solutions of 10 multivalent ligands (**L**$_X$ and **H**$_X$) at 600 μM in deionized water were diluted serially with culture media to prepare samples of the following concentrations: 10^{-13}, 10^{-12}, 10^{-11}, 10^{-10}, 10^{-9}, 10^{-8}, 10^{-7}, 10^{-6}, and 10^{-5} M. U87MG cells were seeded in three flat-bottomed 96-well microculture plates each at a density of 5 × 10^3 cells per well (for 24 h incubation), 2 × 10^3 cells per well (for 48 h incubation), or 1 × 10^3 cells per well (for 72 h incubation). Each well was filled with 100 μL of culture media, and the plates were incubated for 24 h at 37 °C to allow cell attachment. Next, cells were treated with 100 μL of either each dilution or culture media (as a control) per well and placed in a humidified incubator at 37 °C with 5% CO_2 for 24, 48, or 72 h. The formulations were removed, and cells were quickly rinsed with Dulbecco's phosphate-

buffered saline (D-PBS; Gibco) once. Cells were then treated with a premixed solution (110 μL) composed of 100 μL of fresh MEM and 10 μL of Cell Counting Kit-8 (CCK-8; Dojindo) per well, and incubated for additional 2 h. The absorbance at 450 nm, which is proportional to the number of live cells, was measured and normalized against that of the control (*i.e.*, 100%) prepared under the same conditions. All experiments were repeated twice in triplicate (*i.e.*, $n = 6$), and the results are shown as the mean ± SD in Fig. S36.

***In Vitro* Competitive Binding Assays**

To determine the relative binding affinity of our multivalent ligands (*i.e.*, targeted agents) at the $\alpha_v\beta_3$ integrin receptor, their inhibitory effect was estimated in IC_{50} values against radiolabeled [^{125}I]echistatin (an $\alpha_v\beta_3$ integrin-specific binder; PerkinElmer).[6,7] Specifically, U87MG cells were harvested and seeded in 24-well plates at a density of 1×10^5 cells per well, each well was filled with 200 μL of serum-free MEM, and the cells were incubated overnight at 37 °C to allow their attachment. Stock solutions (600 μM) of targeted (**L$_X$** and **H$_X$**) and untargeted (**PL$_X$** and **PH$_X$**) agents and c(RGDfK) (**4**, Fig. S4) as a monovalent control, all in deionized water, were diluted serially with Hank's balanced salt solution (HBSS; Gibco) to prepare samples of different concentrations ranging from 2.4×10^{-14} to 2.4×10^{-5} M. Cells were treated immediately with 200 μL of each warmed dilution per well and incubated for 30 min at 37 °C. Subsequently, 50 μL of [^{125}I]echistatin diluted with HBSS (all at 37 Bq per well) was added to each well, and the cells were placed in a shaking incubator at 37 °C for 60 min. Cells were then washed twice with cold HBSS, and 0.5 mL of 1% SDS was added to each well to facilitate cell lysis. The lysates were collected from each well, and the radioactivity was measured using a Packard Cobra gamma counter. The radioactivity was normalized against the amount of protein included in each sample. The inhibitory concentrations of our dendrimer conjugates and c(RGDfK) (**4**) that reduced the specific binding of [^{125}I]echistatin at $\alpha_v\beta_3$ integrin by 50% (IC_{50}) were calculated by nonlinear regression analysis (sigmoidal dose response equation) using GraphPad Prism 6 software. All experiments were repeated twice in triplicate (*i.e.*, $n = 6$), and the results are shown as the mean ± SD in Fig. 3a,b and Table S4.

Cellular Uptake Studies

Stock solutions (600 μM) of targeted (**L_X** and **H_X**) and untargeted (**PL_X** and **PH_X**) agents in deionized water were diluted with culture media (MEM containing 10% FBS and antibiotic–antimycotic) to prepare solutions at 1.8 μM.

For confocal fluorescence microscopy experiments, U87MG cells were seeded in a μ-Slide 8-well microscope sample chamber at a density of 3×10^4 cells per well and incubated for 24 h at 37 °C to allow cell attachment. Cells were treated with 200 μL of each dilution per well, and incubated for 24 h at 37 °C. Cells were then washed with D-PBS and fixed by treating with 4% paraformaldehyde (PFA) for 10 min at room temperature. Subsequently, the fixed cells were washed with D-PBS (Welgene) to remove PFA, nuclear stained with 4′,6-diamidino-2-phenylindole (DAPI) for 5 min at room temperature, and washed with D-PBS. Next, the internalization profiles of our nano-sized agents into U87MG cells were obtained using a Zeiss LSM800 confocal scanning laser microscope (Carl Zeiss, Thornwood, CA, USA) with filter sets of Cy5 (λ_{ex} 633 nm, λ_{em} 633-710 nm) and DAPI (λ_{ex} 405 nm, λ_{em} 420-480 nm). The results are shown in Fig. 3c,d and S37.

For flow cytometry experiments, U87MG cells were seeded in a flat bottomed 6-well plate (Falcon) at a density of 2×10^5 cells per well and incubated for 24 h at 37 °C to allow cell attachment. Cells were treated with 2 mL of either each dilution or culture media (as a control) per well, and incubated for 24 h at 37 °C. Cells were washed three times with cold D-PBS (pH 7.4, Welgene), fixed by treating with 4% PFA for 2 min at room temperature, and washed three times with cold D-PBS. Next, the cells were detached from the 6-well plate by incubating with 0.25% trypsin-EDTA (1X, pH 7.2-8.0, Gibco) for 5 min at 37 °C, and centrifuged at 1000 rpm for 5 min to obtain a cell pellet. After removal of the supernatant, the cell pellet was treated with D-PBS and centrifuged. The previous procedure of removing the supernatant, adding D-PBS, and centrifugation to obtain the cell pellet was repeated. Subsequently, the cells were resuspended in 1 mL of D-PBS and analyzed using a Becton Dickinson FACSCalibur flow cytometer ($_{ex}$ 488 ± 5 nm, $_{em}$ 695 ± 20 nm (= FL3)) and the *CellQuest* Pro software. For each sample, 10000 cells were recorded. The results are shown in Fig. 3e,f.

In Vivo SPECT Imaging Experiments

All animal studies were approved by the Institutional Animal Care and Use Committee of the National Cancer Center.

20 μL (12 nmol) of each stock solution (600 μM) of targeted (**L_X** and **H_X**) and untargeted (**PL_X** and **PH_X**) agents in deionized water and 50 μL of [^{125}I]NaI (PerkinElmer) in saline at a concentration of 18.5 MBq were added into an Iodogen (pre-coated iodination tube; Pierce), and the reaction mixture was placed in a shaking incubator at 25 °C for 10 min. The degree of radioiodination (*i.e.*, labeling efficiency) was higher than 95% for all of our dendrimer conjugates as determined by instant thin layer chromatography (ITLC) in 0.9% saline (eluent). The entire crude reaction mixture of each ^{125}I-labeled dendrimer conjugate (70 μL) was used for intravascular injection into a mouse without purification.

Next, U87MG cells (1 × 10^6 cells in 200 μL of PBS) were inoculated subcutaneously into the right hind hip of each 4-week-old female BALB/c nude mouse (Orient Bio). Once the tumor size reached 5 mm or larger by diameter, the crude reaction mixture of each ^{125}I-labeled dendrimer conjugate (*ca.* 12 nmol, 70 μL, 18.5 MBq) was injected into the mouse tail vein. The SPECT images of these mice were acquired using a NanoSPECT animal SPECT imaging system (Bioscan) at 2 h post-injection (hpi) and 24 hpi. The obtained SPECT images were analyzed using InVivoScope software (Bioscan), and the results (range: 0-3 kBq) are shown in Fig. 4 and S38, and Movies S1 and S2.

Biodistribution Studies

5.0 μL (3.0 nmol) of each stock solution (600 μM) of selected targeted (**H_{NH2}**, **H_{SA}**, and **H_{PEG}**) and untargeted (**PH_{NH2}**, **PH_{SA}**, and **PH_{PEG}**) agents in deionized water and 50 μL of [^{131}I]NaI (Korea Atomic Energy Research Institute) in saline at a concentration of 3.7 MBq were added into an Iodogen (pre-coated iodination tube; Pierce), and the reaction mixture was placed in a shaking incubator at 25 °C for 10 min. The degree of radioiodination (*i.e.*, labeling efficiency) was higher than 95% for all of the dendrimer conjugates used herein as determined by ITLC in 0.9% saline (eluent). The entire crude reaction mixture of each ^{131}I-labeled dendrimer conjugate (55 μL) was used for intravascular injection into a mouse without purification.

Next, U87MG cells (1×10^6 cells in 200 μL of PBS) were inoculated subcutaneously into the right hind hip of each 4-week-old female BALB/c nude mouse (Orient Bio). Once the tumor size reached 5 mm or larger by diameter, the crude reaction mixture of each ^{131}I-labeled dendrimer conjugate (*ca.* 3.0 nmol, 55 μL, 3.7 MBq (*i.e.*, 100 μCi)) was injected into the mouse tail vein. The mice were sacrificed at either 2 hpi or 24 hpi, and their target organs (brain, blood, heart, lung, liver, spleen, kidney, stomach, intestine, femur, muscle, and tumor; Fig. S39) were collected, harvested, and weighed. The radioactivity of each organ was measured using a PerkinElmer 1480 WIZARD 3″ automatic gamma counter. Finally, the percent of injected dose per gram of tissue (% ID/g) for each organ was calculated from the radioactivity data. All experiments were performed in triplicate, and the results are shown as the mean ± SD in Fig. 5a,b and Table S5. Statistical analysis was performed by the unpaired *t*-test using the GraphPad Prism 5.0 software. P values less than 0.05 were considered significant. Additionally, from the biodistribution results, tumor-targeting efficiency for up to 24 hpi was estimated quantitatively as the area-under-the-curve (AUC; Fig. 5c) based on the non-compartmental linear trapezoidal analysis model.[8] For all compounds, one additional time point, 0 hpi (*i.e.*, before injection, 0% ID/g), was considered for the estimation of AUC. The relative tumor-targeting efficiency was also calculated as the tumor-to-organ ratios (mean ± SD, $n = 3$) as shown in Fig. 5d and Table S6.

Table S1. Structural analysis of synthesized dendrimer conjugates by NMR and MALDI-TOF MS.

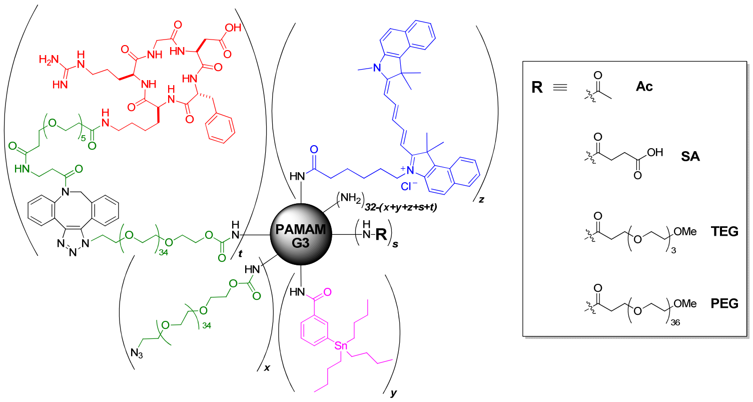

cmpd	NMR[a]									MALDI-TOF MS[d]		
	x	y	z[b]	s				t	MW[c]	M_n[e]	M_w[f]	PDI[g]
				**Ac**	**SA**	**TEG**	**PEG**					
**PPL**	5.09	—	—	—	—	—	—	—	15336	15214	15615	1.03
**PL$_{NH2}$**	5.07	1.93	0.50	—	—	—	—	—	16360	13858	14292	1.03
**L$_{NH2}$**	0.62	1.93	0.50	—	—	—	—	4.45	21621	24549	25697	1.05
**PL$_{12Ac}$**	5.04	1.86	0.50	12.14	—	—		—	16801	13888	14177	1.02
**L$_{12Ac}$**	1.04	1.86	0.50	12.14	—	—	—	4.00	21530	20286	21159	1.04
**PL$_{19Ac}$**	5.00	2.04	0.50	18.65	—	—	—	—	17072	13411	13967	1.04
**L$_{19Ac}$**	0.79	2.04	0.50	18.65	—	—		4.21	22050	18994	20065	1.06
**PL$_{SA}$**	5.08	1.93	0.50	—	18.53	—	—	—	18223	15426	15686	1.02
**L$_{SA}$**	0.12	1.93	0.50	—	18.53	—	—	4.96	24087	20772	22092	1.06
**PL$_{TEG}$**	5.06	1.97	0.50	—	—	20.97	—	—	20927	16888	17711	1.05
**L$_{TEG}$**	0.54	1.97	0.50	—	—	20.97	—	4.52	26265	21848	23172	1.06
**PL$_{PEG}$**	4.72	2.00	0.50	—	—	—	20.18	—	49547	40921	41946	1.03
**L$_{PEG}$**	0.45	2.00	0.50	—	—	—	20.18	4.27	54590	45533	47176	1.04
**PPH**	10.89	—	—	—	—	—	—	—	24937	18658	19848	1.06
**PH$_{NH2}$**	10.57	1.59	0.50	—	—	—	—	—	25327	19790	20302	1.03
**H$_{NH2}$**	0.21	1.59	0.50	—	—	—	—	10.36	37570	31426	33447	1.06
**PH$_{SA}$**	11.18	1.82	0.50	—	18.38	—	—	—	28263	21796	22785	1.05
**H$_{SA}$**	1.03	1.82	0.50	—	18.38	—	—	10.15	40264	30584	33414	1.09
**PH$_{TEG}$**	10.75	1.59	0.50	—	—	17.28	—	—	29403	22545	23275	1.03
**H$_{TEG}$**	0.55	1.59	0.50	—	—	17.28	—	10.20	41463	29445	31082	1.06
**PH$_{PEG}$**	11.66	1.74	0.50	—	—	—	15.83	—	53651	41295	42156	1.02
**H$_{PEG}$**	0.96	1.74	0.50	—	—	—	15.83	10.70	66302	50296	52053	1.03
**G3-32PEG**	—	—	—	—	—	—	32.00	—	60412	47281	48795	1.03

[a] Based on the NMR integration in DMSO-d_6 (see Fig. S12-S23): x = number of azido-PEG units; y = number of 3-(tri-n-butylstannyl)benzoyl groups; z = number of Cy5.5; s = number of modified surface functional groups; t = number of c(RGDfK). [b] Based on the stoichiometry of addition. [c] For MW estimation, dendrimer conjugates were assumed to be made from the G3 PAMAM dendrimer with 32 peripheral amino groups without any structural defects. Also, each Cy5.5 moiety was assumed to have chloride anion as a counterion. [d] Determined using DHB as a matrix. [e] Number-average molar mass. [f] Weight-average molar mass. [g] Polydispersity index.

Table S2. Structural parameters (homogeneous sphere model) obtained from SAXS data of dendrimer conjugates at 25 °C in 2.5 mM NaCl solution (450 μM, pH 7.4).

cmpd	$R_{g,G}{}^{a}$ (nm)	$R^{b} \pm \sigma_{R}{}^{c}$ (nm)	R^{d} (nm)
**L_{NH2}**	8.91 ± 0.38	9.84 ± 0.44	11.50
**L_{12Ac}**	5.16 ± 0.12	7.48 ± 0.42	6.66
**L_{19Ac}**	5.25 ± 0.13	6.72 ± 0.38	6.78
**L_{SA}**	4.55 ± 0.15	6.39 ± 0.41	5.87
**L_{TEG}**	5.23 ± 0.14	6.69 ± 0.44	6.75
**L_{PEG}**	4.04 ± 0.11	5.30 ± 0.39	5.22
**H_{NH2}**	5.18 ± 0.11	7.59 ± 0.44	6.69
**H_{SA}**	5.60 ± 0.13	7.40 ± 0.49	7.23
**H_{TEG}**	6.85 ± 0.20	8.40 ± 0.50	8.84
**H_{PEG}**	4.06 ± 0.07	5.08 ± 0.31	5.24
**PL_{NH2}**	3.68 ± 0.13	5.47 ± 0.43	4.75
**PL_{12Ac}**	3.29 ± 0.18	4.89 ± 0.47	4.25
**PL_{19Ac}**	3.85 ± 0.18	5.44 ± 0.38	4.97
**PL_{SA}**	4.43 ± 0.14	6.08 ± 0.43	5.72
**PL_{TEG}**	4.33 ± 0.21	5.56 ± 0.41	5.59
**PL_{PEG}**	N/A[e]	N/A[e]	N/A[e]
**PH_{NH2}**	4.29 ± 0.16	5.89 ± 0.50	5.54
**PH_{SA}**	3.28 ± 0.08	4.74 ± 0.47	4.23
**PH_{TEG}**	5.56 ± 0.25	5.85 ± 0.50	7.18
**PH_{PEG}**	3.72 ± 0.09	4.57 ± 0.37	4.80

[a] Radius of gyration obtained from the scattering data by the Guinier analysis.
[b] Average sphere radius estimated from the particle size distribution.
[c] Relative standard deviation (RSD), σ_R = standard deviation/mean value.
[d] Radius obtained as $R = R_{g,G} / (3/5)^{1/2}$.
[e] Data were not obtained due to the limited supply of samples.

Table S3. Surface charge of targeted (**L_X** and **H_X**) and untargeted (**PL_X** and **PH_X**) agents at 25 °C.[a]

cmpd	zeta potential[b] (mV)	cmpd	zeta potential[b] (mV)
**L_{NH2}**	6.29 ± 0.29	**PL_{NH2}**	16.50 ± 0.26
**L_{12Ac}**	4.69 ± 1.52	**PL_{12Ac}**	5.88 ± 0.78
**L_{19Ac}**	2.65 ± 0.48	**PL_{19Ac}**	3.64 ± 0.05
**L_{SA}**	-5.75 ± 0.90	**PL_{SA}**	-9.59 ± 0.56
**L_{TEG}**	3.74 ± 1.10	**PL_{TEG}**	5.50 ± 1.08
**L_{PEG}**	-3.94 ± 0.08	**PL_{PEG}**	1.33 ± 0.41
**H_{NH2}**	4.77 ± 0.48	**PH_{NH2}**	8.31 ± 0.46
**H_{SA}**	-5.59 ± 0.59	**PH_{SA}**	-13.33 ± 0.45
**H_{TEG}**	1.86 ± 0.97	**PH_{TEG}**	2.50 ± 0.28
**H_{PEG}**	-1.74 ± 1.20	**PH_{PEG}**	2.72 ± 0.23

[a] Results are shown as the mean ± standard deviation (SD) ($n = 3$).
[b] Samples were prepared in 10 mM NaCl (pH 7.4) at 100 µg/mL.

Table S4. Inhibitory effect (IC_{50}) of multivalent dendritic ligands (*i.e.*, targeted agents) and c(RGDfK) (a monovalent control) on binding of [^{125}I]echistatin to the $\alpha_v\beta_3$ integrin receptors on U87MG cells.[a]

cmpd	$\log(IC_{50})^b$	IC_{50} (M)^c
$\mathbf{L_{NH2}}$	-8.53 ± 0.20	2.95×10^{-9}
$\mathbf{L_{12Ac}}$	-7.47 ± 0.12	3.35×10^{-8}
$\mathbf{L_{19Ac}}$	-6.53 ± 0.24	2.95×10^{-7}
$\mathbf{L_{SA}}$	-6.27 ± 0.32	5.38×10^{-7}
$\mathbf{L_{TEG}}$	-6.40 ± 0.13	3.96×10^{-7}
$\mathbf{L_{PEG}}$	-6.39 ± 0.23	4.05×10^{-7}
$\mathbf{H_{NH2}}$	-9.42 ± 0.13	3.77×10^{-10}
$\mathbf{H_{SA}}$	-6.47 ± 0.13	3.41×10^{-7}
$\mathbf{H_{TEG}}$	-6.42 ± 0.12	3.84×10^{-7}
$\mathbf{H_{PEG}}$	-6.36 ± 0.31	4.40×10^{-7}
c(RGDfK)	-5.38 ± 0.08	4.22×10^{-6}

[a] No binding was detected (or binding was not measurable) for the untargeted agents ($\mathbf{PL_X}$ or $\mathbf{PH_X}$) under the given assay conditions, and thus their IC_{50} values are not listed herein. See Fig. 3a,b in the main text for the binding curves. [b] Results, determined by the nonlinear regression analysis (sigmoidal dose response equation) using the GraphPad Prism 6 software, are shown as the mean ± SD (n = 6). [c] Calculated from the mean value of the corresponding $\log(IC_{50})$.

Table S5. Biodistribution (% ID/g) at 2 hpi and 24 hpi of selected tumor-targeting agents radiolabeled with iodine-131 (mean ± SD, n = 3).

time	organ	targeted agents			untargeted agents		
		[^{131}I]$\mathbf{H}_{\mathbf{NH2}}$	[^{131}I]$\mathbf{H}_{\mathbf{SA}}$	[^{131}I]$\mathbf{H}_{\mathbf{PEG}}$	[^{131}I]$\mathbf{PH}_{\mathbf{NH2}}$	[^{131}I]$\mathbf{PH}_{\mathbf{SA}}$	[^{131}I]$\mathbf{PH}_{\mathbf{PEG}}$
2 h post-injection	brain	0.24 ± 0.07	0.21 ± 0.09	0.21 ± 0.04	0.32 ± 0.21	0.26 ± 0.04	0.31 ± 0.07
	blood	7.01 ± 0.69	8.61 ± 0.37	7.58 ± 0.33	1.87 ± 0.29	5.76 ± 0.34	5.57 ± 1.14
	heart	3.73 ± 1.27	2.19 ± 0.62	2.60 ± 0.11	1.87 ± 0.49	2.65 ± 0.64	2.24 ± 0.42
	lung	5.49 ± 2.13	4.27 ± 1.56	5.00 ± 0.19	3.62 ± 1.31	4.79 ± 1.89	4.30 ± 0.89
	liver	8.33 ± 0.34	8.19 ± 0.56	7.04 ± 1.48	5.02 ± 1.43	7.12 ± 0.85	3.69 ± 0.93
	spleen	7.51 ± 1.68	7.02 ± 1.36	5.04 ± 0.75	2.77 ± 0.53	3.09 ± 0.69	2.82 ± 0.59
	kidney	7.96 ± 1.51	7.88 ± 1.71	5.69 ± 0.88	7.22 ± 1.21	3.61 ± 1.41	3.95 ± 0.95
	stomach	6.91 ± 0.49	6.13 ± 1.01	5.32 ± 1.59	2.27 ± 0.34	5.07 ± 2.32	2.79 ± 0.46
	intestine	3.87 ± 1.58	2.08 ± 1.06	2.25 ± 0.69	3.08 ± 1.00	3.88 ± 2.01	3.48 ± 1.24
	femur	2.26 ± 0.99	1.73 ± 0.67	1.80 ± 0.43	0.46 ± 0.38	0.92 ± 0.67	0.79 ± 0.29
	muscle	1.79 ± 1.32	0.89 ± 0.31	1.26 ± 0.61	1.53 ± 0.35	1.55 ± 0.24	1.06 ± 0.27
	tumor	4.75 ± 2.21	7.50 ± 1.88	3.64 ± 1.13	2.51 ± 1.80	7.89 ± 0.68	3.68 ± 0.69
24 h post-injection	brain	0.07 ± 0.01	0.22 ± 0.10	0.24 ± 0.05	0.03 ± 0.00	0.11 ± 0.03	0.11 ± 0.04
	blood	2.67 ± 0.33	2.05 ± 0.55	2.41 ± 0.26	0.80 ± 0.04	2.34 ± 0.74	4.53 ± 0.62
	heart	1.28 ± 0.14	1.68 ± 0.64	1.78 ± 0.06	0.69 ± 0.07	1.39 ± 0.29	1.93 ± 0.42
	lung	2.21 ± 0.11	2.46 ± 0.89	1.69 ± 0.37	0.54 ± 0.13	2.32 ± 0.85	3.51 ± 1.10
	liver	7.58 ± 0.94	6.61 ± 2.41	6.41 ± 1.39	3.44 ± 0.30	5.02 ± 1.44	3.23 ± 0.61
	spleen	6.08 ± 1.80	5.48 ± 1.62	3.35 ± 0.42	0.69 ± 0.18	2.32 ± 0.85	2.96 ± 0.38
	kidney	7.39 ± 0.83	2.65 ± 0.40	2.21 ± 0.19	5.52 ± 0.67	2.12 ± 0.97	1.59 ± 1.12
	stomach	1.81 ± 0.22	2.26 ± 0.09	2.22 ± 0.89	0.57 ± 0.12	1.47 ± 0.60	1.78 ± 0.50
	intestine	1.55 ± 0.28	1.24 ± 0.46	1.79 ± 0.74	0.70 ± 0.20	1.06 ± 0.31	1.25 ± 0.30
	femur	1.26 ± 0.19	1.18 ± 0.57	1.57 ± 0.50	0.28 ± 0.13	0.74 ± 0.25	0.68 ± 0.15
	muscle	0.40 ± 0.07	0.82 ± 0.25	0.81 ± 0.48	0.11 ± 0.02	0.42 ± 0.21	0.63 ± 0.21
	tumor	2.90 ± 0.30	3.75 ± 0.48	3.44 ± 0.61	0.82 ± 0.37	7.82 ± 0.44	6.85 ± 1.60

Table S6. Relative tumor-targeting efficiency of selected nano-sized dendritic agents radiolabeled with iodine-131 estimated as the tumor-to-organ ratios (mean ± SD, n = 3) from biodistribution (% ID/g) at 2 hpi and 24 hpi.

time	ratio	targeted agents			untargeted agents		
		$[^{131}I]\mathbf{H}_{\mathbf{NH2}}$	$[^{131}I]\mathbf{H}_{\mathbf{SA}}$	$[^{131}I]\mathbf{H}_{\mathbf{PEG}}$	$[^{131}I]\mathbf{PH}_{\mathbf{NH2}}$	$[^{131}I]\mathbf{PH}_{\mathbf{SA}}$	$[^{131}I]\mathbf{PH}_{\mathbf{PEG}}$
2 h post-injection	tumor/brain	18.69 ± 4.06	39.45 ± 11.50	17.15 ± 5.39	10.95 ± 9.74	31.42 ± 7.27	12.18 ± 3.75
	tumor/blood	0.67 ± 0.28	0.87 ± 0.23	0.48 ± 0.15	1.38 ± 0.99	1.38 ± 0.20	0.69 ± 0.26
	tumor/heart	1.29 ± 0.50	3.53 ± 0.88	1.39 ± 0.39	1.29 ± 0.64	3.09 ± 0.71	1.72 ± 0.62
	tumor/lung	0.86 ± 0.21	1.88 ± 0.57	0.72 ± 0.21	0.65 ± 0.24	1.82 ± 0.69	0.90 ± 0.35
	tumor/liver	0.58 ± 0.28	0.91 ± 0.19	0.53 ± 0.21	0.47 ± 0.23	1.12 ± 0.11	1.05 ± 0.38
	tumor/spleen	0.61 ± 0.18	1.06 ± 0.07	0.75 ± 0.34	0.87 ± 0.51	2.64 ± 0.59	1.35 ± 0.43
	tumor/kidney	0.60 ± 0.26	0.96 ± 0.19	0.63 ± 0.14	0.34 ± 0.21	2.47 ± 1.11	1.00 ± 0.43
	tumor/stomach	0.68 ± 0.27	1.25 ± 0.42	0.68 ± 0.06	1.19 ± 0.98	1.80 ± 0.81	1.31 ± 0.07
	tumor/intestine	1.28 ± 0.54	3.99 ± 1.23	1.62 ± 0.33	0.87 ± 0.67	2.46 ± 1.34	1.12 ± 0.38
	tumor/femur	2.11 ± 0.53	4.59 ± 1.13	2.01 ± 0.33	10.83 ± 13.03	11.71 ± 7.02	5.28 ± 2.49
	tumor/muscle	2.99 ± 0.89	8.94 ± 2.50	3.20 ± 1.47	1.53 ± 0.75	5.17 ± 0.81	3.56 ± 0.84
24 h post-injection	tumor/brain	40.81 ± 8.91	20.18 ± 10.73	15.19 ± 4.67	31.07 ± 15.59	75.92 ± 26.76	67.01 ± 24.55
	tumor/blood	1.10 ± 0.24	1.95 ± 0.75	1.46 ± 0.40	1.01 ± 0.43	3.59 ± 1.26	1.56 ± 0.58
	tumor/heart	2.30 ± 0.49	2.54 ± 1.29	1.93 ± 0.29	1.20 ± 0.57	5.85 ± 1.56	3.83 ± 1.88
	tumor/lung	1.31 ± 0.09	1.67 ± 0.65	2.06 ± 0.27	1.47 ± 0.53	3.74 ± 1.61	2.20 ± 1.23
	tumor/liver	0.39 ± 0.06	0.62 ± 0.22	0.54 ± 0.02	0.23 ± 0.09	1.67 ± 0.59	2.14 ± 0.41
	tumor/spleen	0.52 ± 0.24	0.72 ± 0.20	1.04 ± 0.25	1.18 ± 0.46	3.79 ± 1.81	2.37 ± 0.78
	tumor/kidney	0.39 ± 0.01	1.44 ± 0.32	1.55 ± 0.15	0.16 ± 0.08	4.32 ± 2.24	8.15 ± 8.95
	tumor/stomach	1.63 ± 0.36	1.65 ± 0.15	1.86 ± 1.15	1.54 ± 0.86	6.23 ± 3.46	4.29 ± 2.43
	tumor/intestine	1.92 ± 0.46	3.28 ± 1.12	2.36 ± 1.59	1.20 ± 0.60	7.84 ± 2.61	5.87 ± 2.78
	tumor/femur	2.37 ± 0.65	3.93 ± 2.34	2.47 ± 1.31	3.17 ± 1.17	11.46 ± 3.73	10.57 ± 3.48
	tumor/muscle	7.53 ± 2.13	4.75 ± 0.87	6.02 ± 4.75	7.93 ± 4.42	22.58 ± 11.98	12.69 ± 8.26

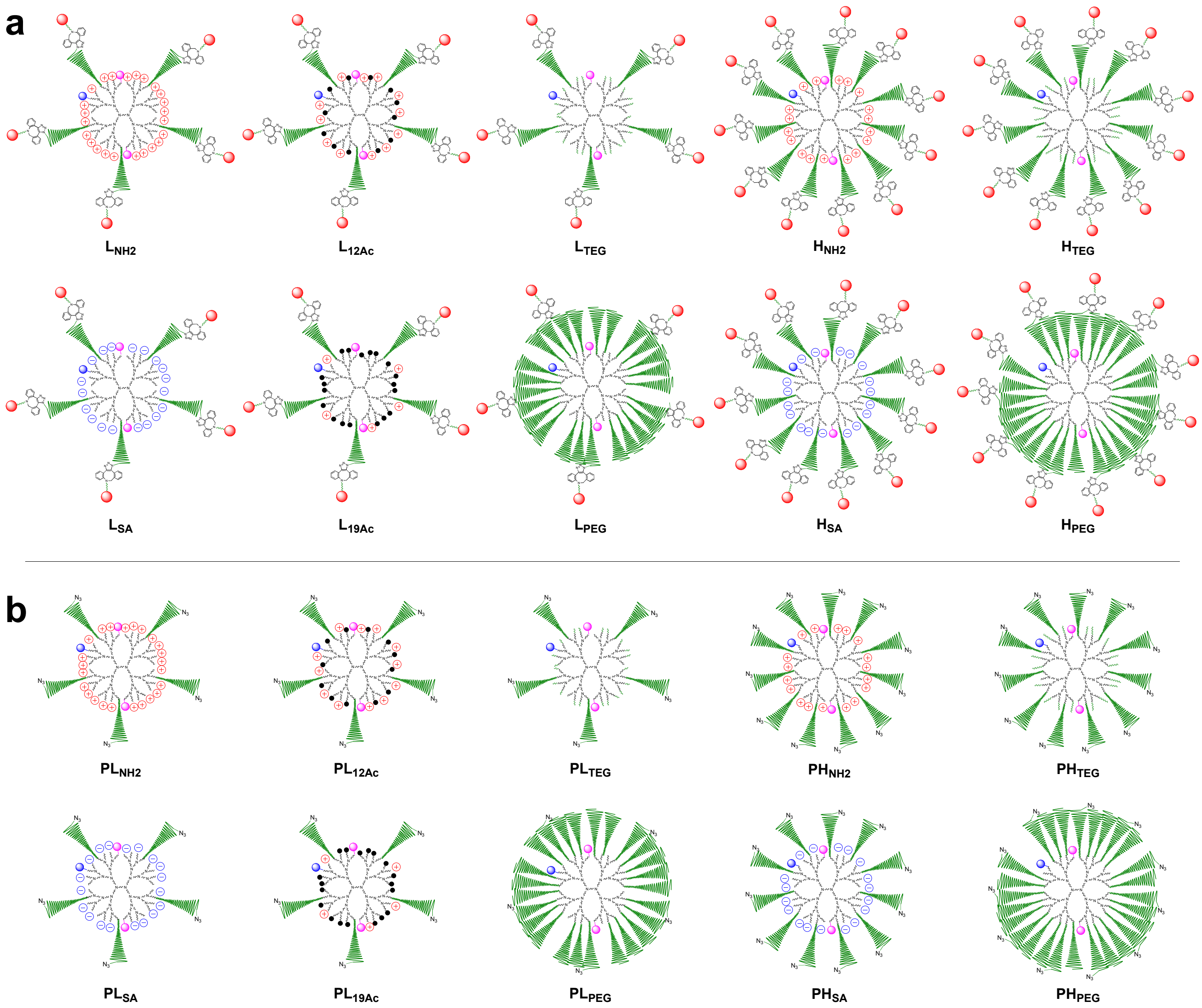

Fig. S1. Schematic illustrations of (a) 10 targeted (**L_X** and **H_X**) and (b) 10 untargeted (**PL_X** and **PH_X**) agents synthesized in this study, which varied by the surface composition (inner functional groups and PEG density), for the systematic comparison of tumor-targeting efficiency. Untargeted agents are the synthetic precursors of the targeted agents.

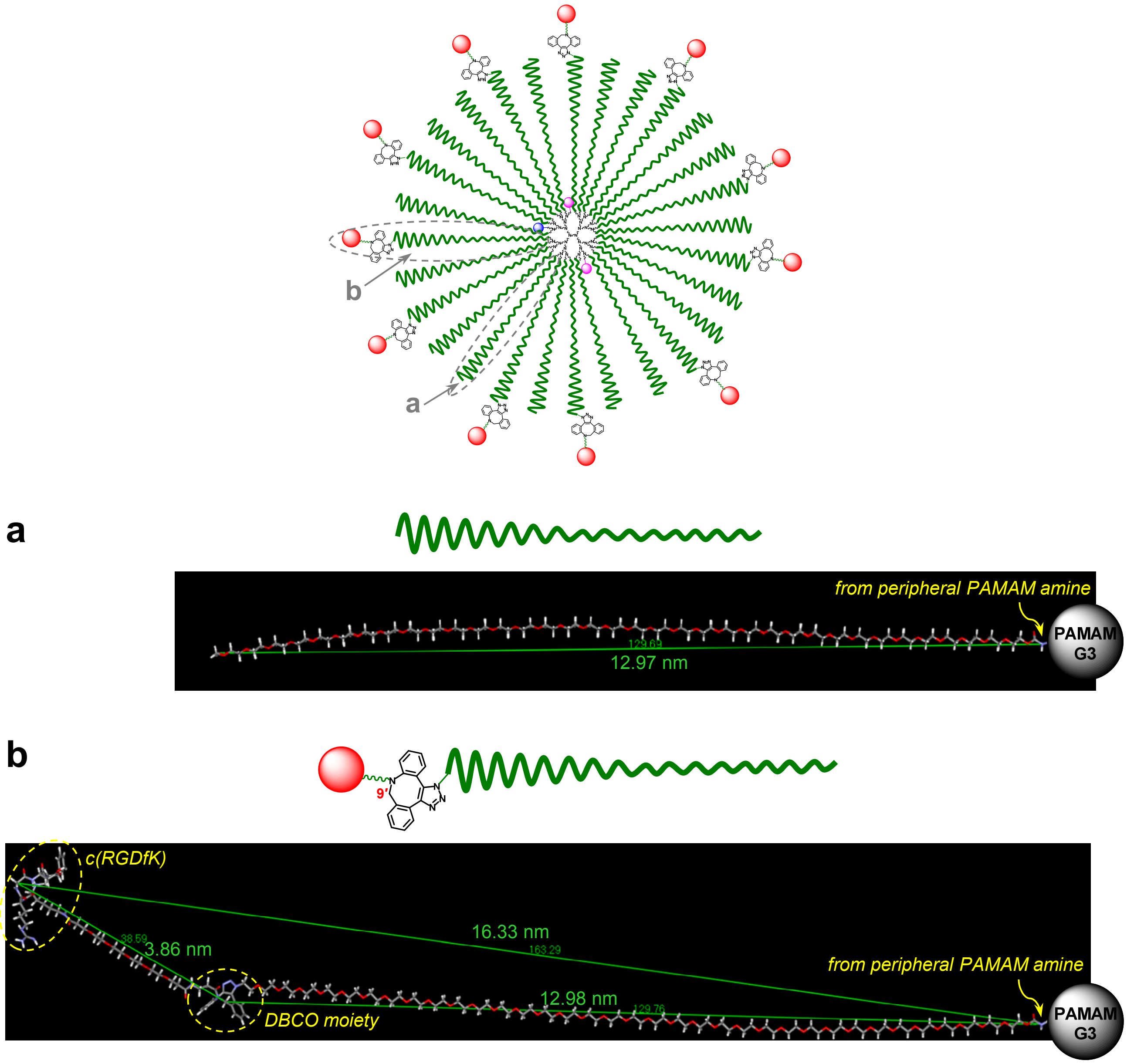

Fig. S2. Comparison of the length of (a) **PEG** as the inner functional group (IFG) and (b) the spacer unit (including the DBCO moiety) in the multivalent ligands, $\mathbf{L_{PEG}}$ and $\mathbf{H_{PEG}}$ (type C, see Fig. 1b in the main text). Energy-minimized structures were obtained using the HyperChem7.5.2 software through a semi-empirical AM1 method, with oligo(ethylene oxide) units in all-*anti* (*i.e.*, fully stretched) conformations. Distances were measured between the following atoms: (a) $C_{methoxy(PEG)}$-$N_{PAMAM\ terminus}$; (b) $C_{methylene(Gly)}$-$N_{PAMAM\ terminus}$, $C9'_{DBCO}$-$N_{PAMAM\ terminus}$, and $C9'_{DBCO}$-$C_{methylene(Gly)}$. The IFG **PEG** was determined to be *ca.* 3 nm shorter than the spacer unit.

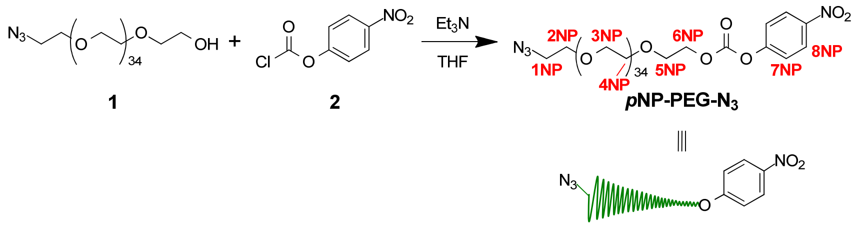

Fig. S3. Synthetic scheme to prepare ***p*NP-PEG-N$_3$**. The labeling method for ^{1}H NMR peak assignments is shown here.

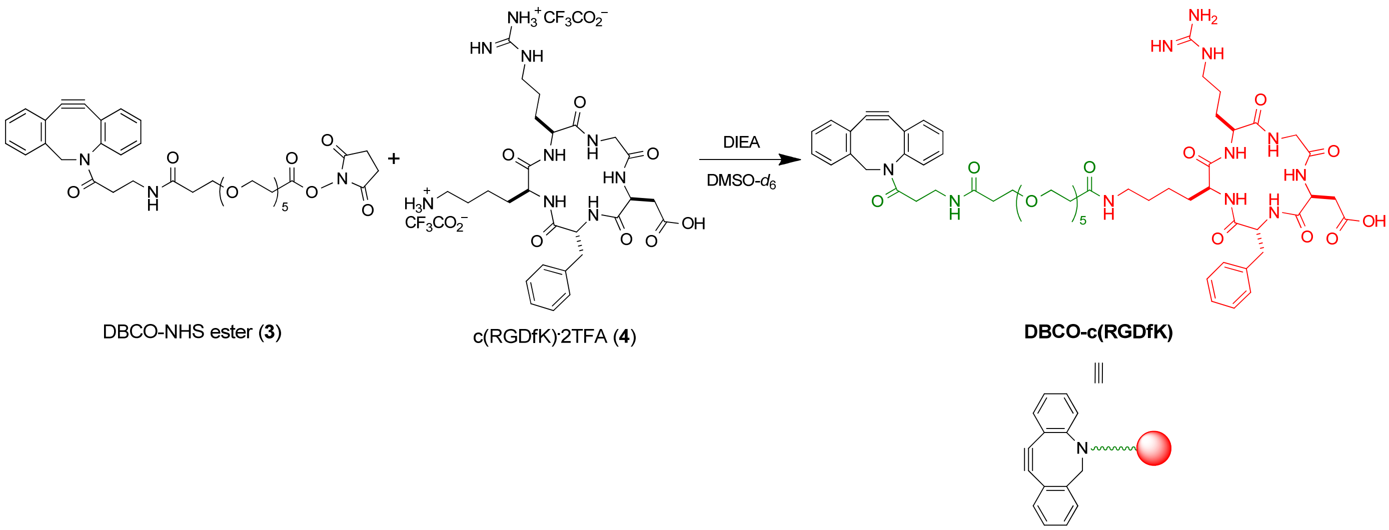

Fig. S4. Synthetic scheme to prepare **DBCO-c(RGDfK)**.

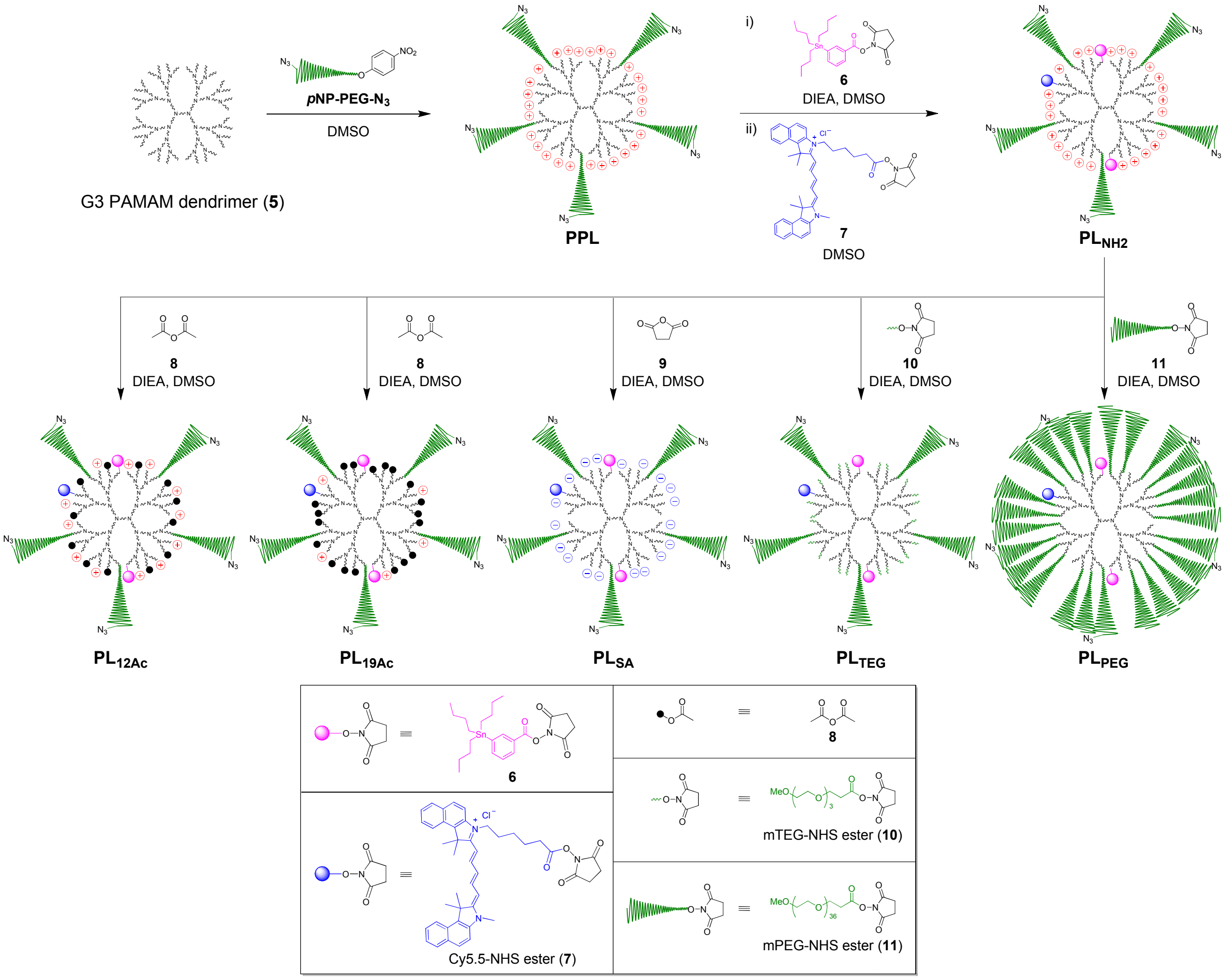

Fig. S5. Synthetic scheme to prepare the precursors of six low-avidity ligands (**PL**$_X$), which were used as passive tumor-targeting agents. For all reactions shown here, anhydrous DMSO was used as a solvent.

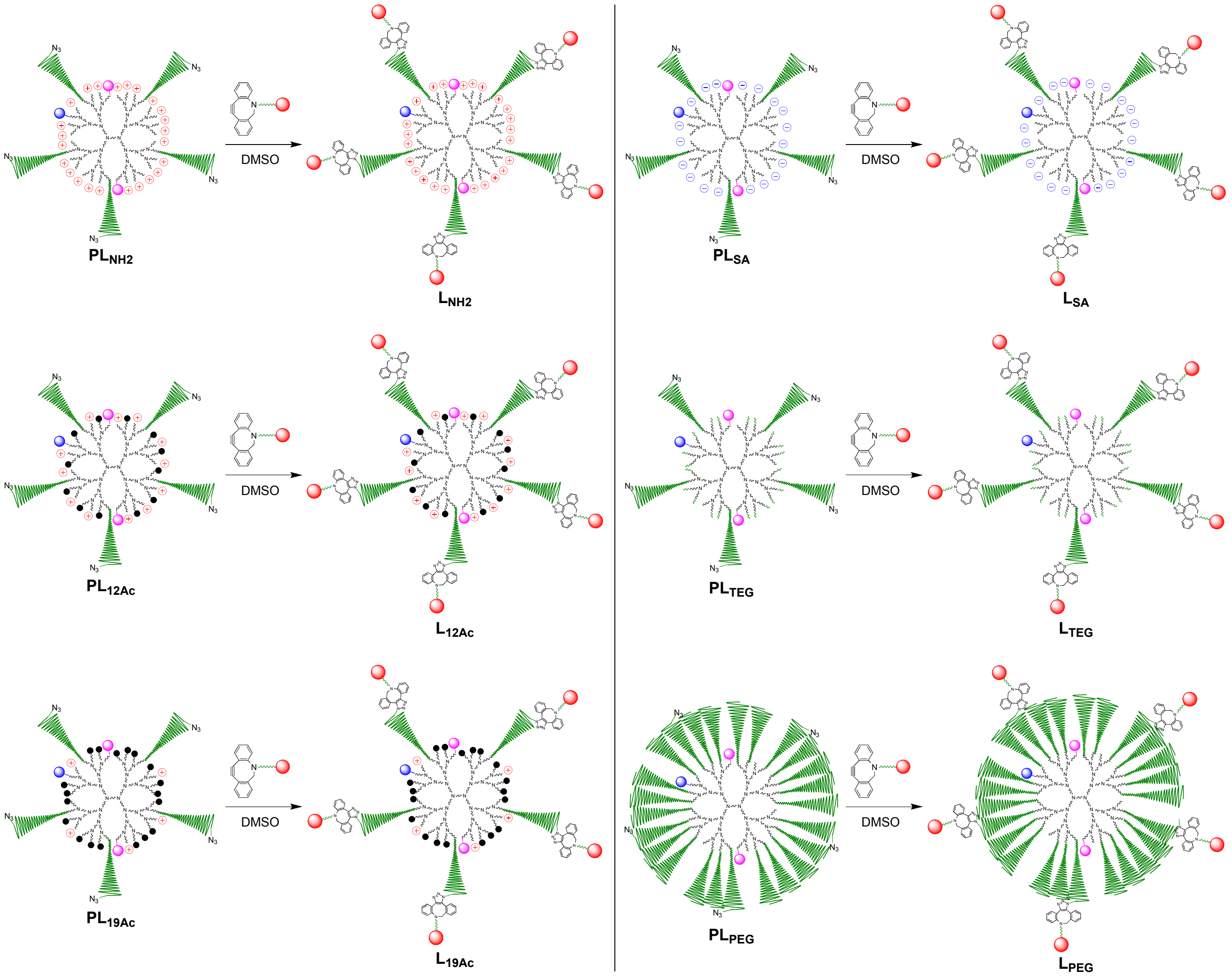

Fig. S6. Synthetic scheme to prepare six low-avidity ligands (**L$_X$**), which were used as active tumor-targeting agents.

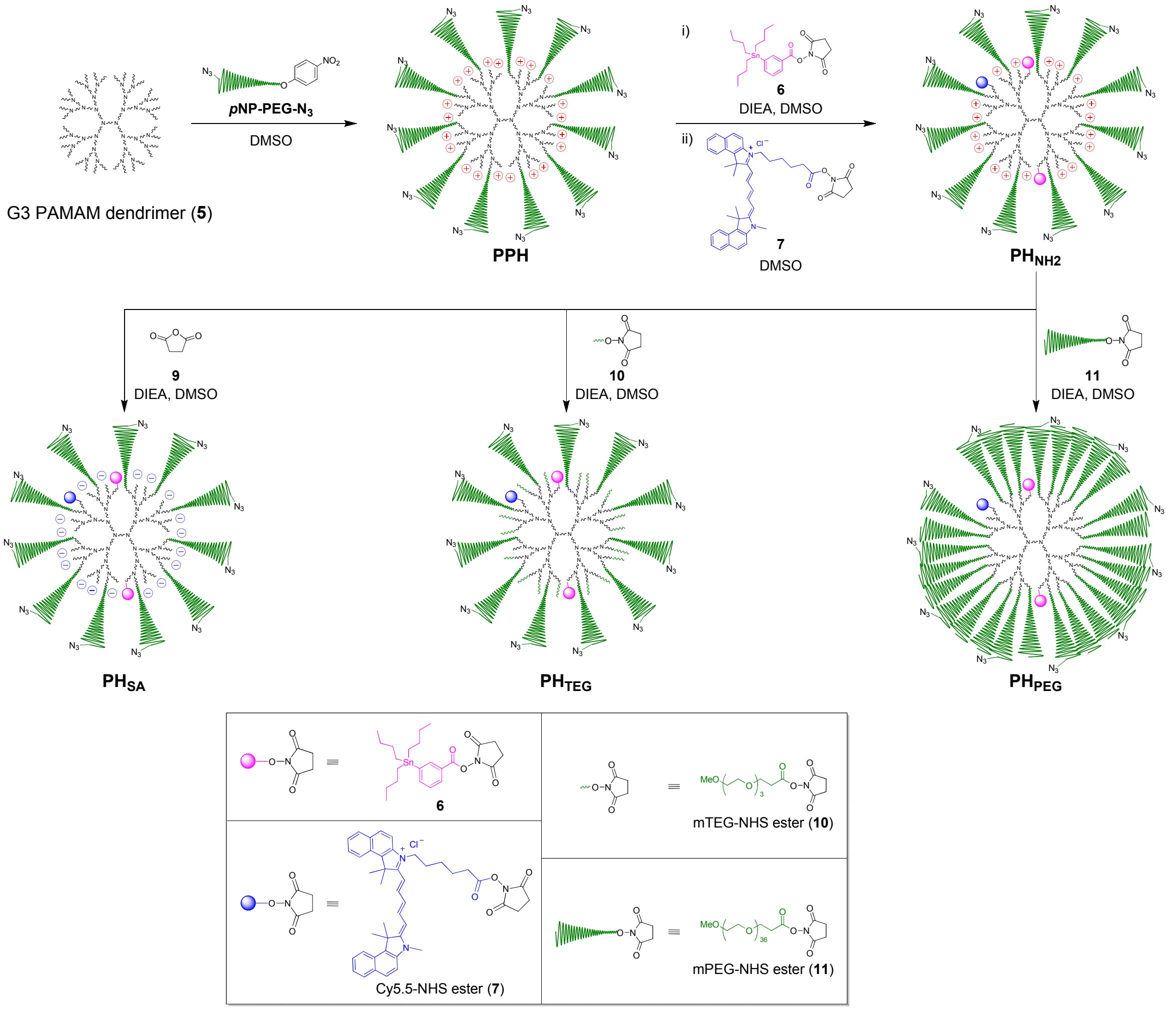

Fig. S7. Synthetic scheme to prepare the precursors of four high-avidity ligands (**PH**$_{\mathbf{X}}$), which were used as passive tumor-targeting agents. For all reactions shown here, anhydrous DMSO was used as a solvent.

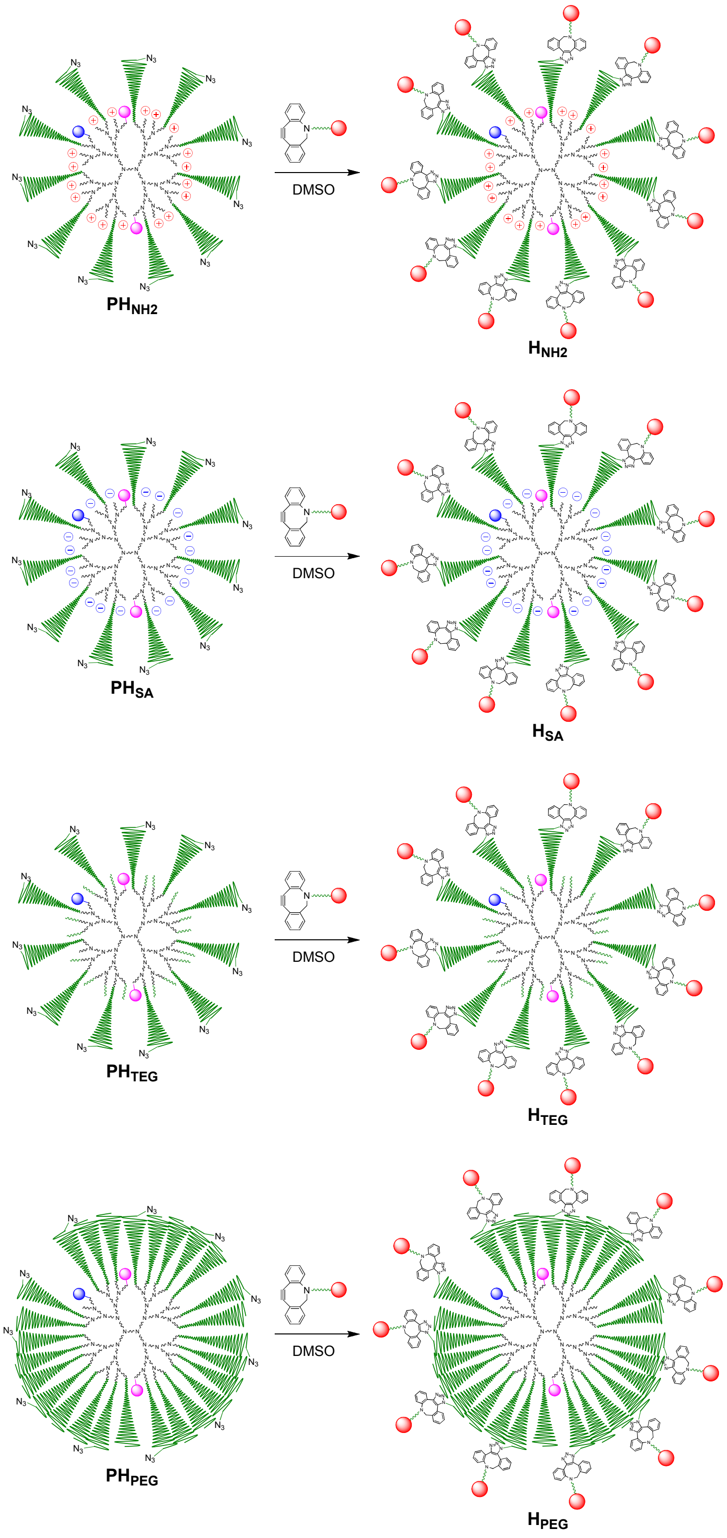

Fig. S8. Synthetic scheme to prepare four high-avidity ligands (**H$_X$**), which were used as active tumor-targeting agents.

a

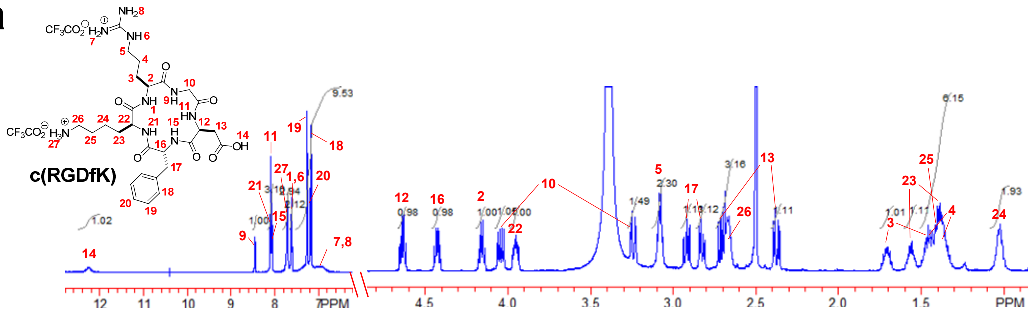

Peak Assignment:
^{1}H NMR (600 MHz, DMSO-d_6) δ 12.26 (br s, 1H, H_{14}), 8.45 (dd, 1H, J = 7.4, 4.6 Hz, H_9), 8.10 (d, 1H, J = 8.0 Hz, H_{21}), 8.08 (d, 1H, J = 8.8 Hz, H_{11}), 8.06 (d, 1H, J = 7.5 Hz, H_{15}), 7.71 (s, 3H, H_{27}), 7.62 (d, 1H, J = 7.8 Hz, H_1), 7.61 (t, 1H, J = 5.7 Hz, H_6), 7.26 (t, 2H, J = 7.5 Hz, H_{19}), 7.18 (t, 1H, J = 7.2 Hz, H_{20}), 7.16 (d, 2H, J = 7.6 Hz, H_{18}), 7.31-6.86 (m, 4H, H_7 and H_8), 4.64 (td, 1H, J = 8.5, 6.1 Hz, H_{12}), 4.43 (q, 1H, J = 7.2 Hz, H_{16}), 4.16 (q, 1H, J = 7.4 Hz, H_2), 4.04 (dd/AMX, 1H, J = 15.0, 7.6 Hz, H_{10}), 3.95 (m, 1H, H_{22}), 3.24 (dd/AMX, 1H, J = 14.8, 4.0 Hz, H_{10}), 3.08 (q, 2H, J = 7.0 Hz, H_5), 2.92, 2.83 (ABX, 2H, H_{17}), 2.71, 2.38 (ABX, 2H, H_{13}), 2.67 (m, 2H, H_{26}), 1.70, 1.47 (m, 2H, H_3), 1.55, 1.39 (m, 2H, H_{23}), 1.46, 1.37 (m, 2H, H_4), 1.41 (m, 2H, H_{25}), 1.03 (m, 2H, H_{24}).

b

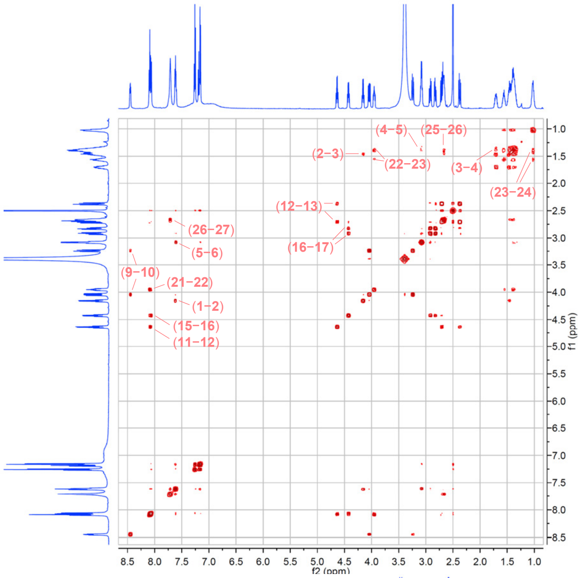

c

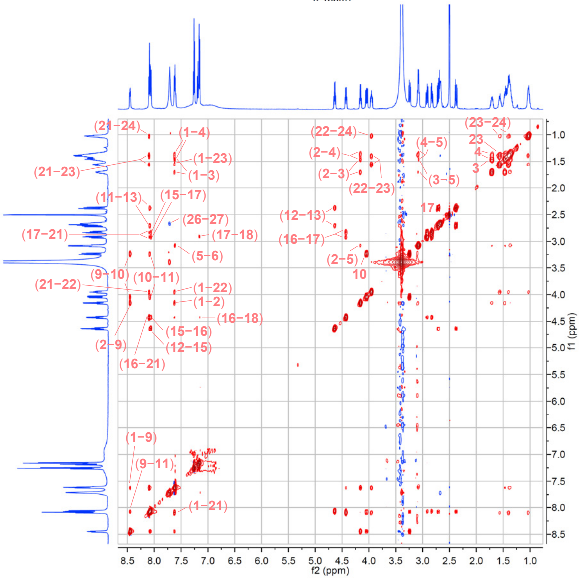

Fig. S9. (a) ^{1}H NMR, (b) COSY, and (c) NOESY spectra of c(RGDfK) in DMSO-d_6.

a

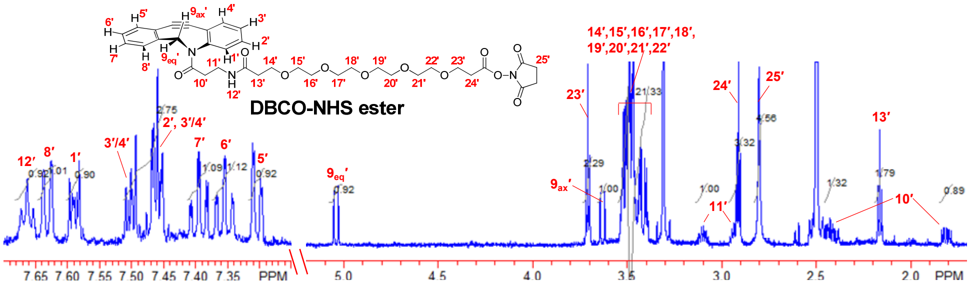

Peak Assignment:

^{1}H NMR (600 MHz, DMSO-d_6) δ 7.66 (t, 1H, J = 5.7 Hz, $H_{12'}$), 7.63 (d, 1H, J = 7.2 Hz, $H_{8'}$), 7.59 (m, 1H, $H_{1'}$), 7.50 (m, 1H, $H_{3'}/H_{4'}$), 7.46 (m, 2H, $H_{3'}/H_{4'}$ and $H_{2'}$), 7.39 (td, 1H, J = 7.4, 1.6 Hz, $H_{7'}$), 7.35 (td, 1H, J = 7.4, 1.2 Hz, $H_{6'}$), 7.30 (dd, 1H, J = 7.6, 1.4 Hz, $H_{5'}$), 5.04 (d, 1H, J = 14.0 Hz, $H_{9eq'}$), 3.71 (t, 2H, J = 6.0 Hz, $H_{23'}$), 3.63 (d, 1H, J = 14.0 Hz, $H_{9ax'}$), 3.54-3.39 (m, 18H, $H_{14'}$, $H_{15'}$, $H_{16'}$, $H_{17'}$, $H_{18'}$, $H_{19'}$, $H_{20'}$, $H_{21'}$, and $H_{22'}$), 3.10 (m, 1H, $H_{11'}$), 2.92 (m, 1H, $H_{11'}$), 2.91 (t, 2H, J = 6.0 Hz, $H_{24'}$), 2.80 (s, 4H, $H_{25'}$), 2.42 (m, 1H, $H_{10'}$), 2.16 (t, 2H, J = 6.5 Hz, $H_{13'}$), 1.81 (m, 1H, $H_{10'}$).

b

c

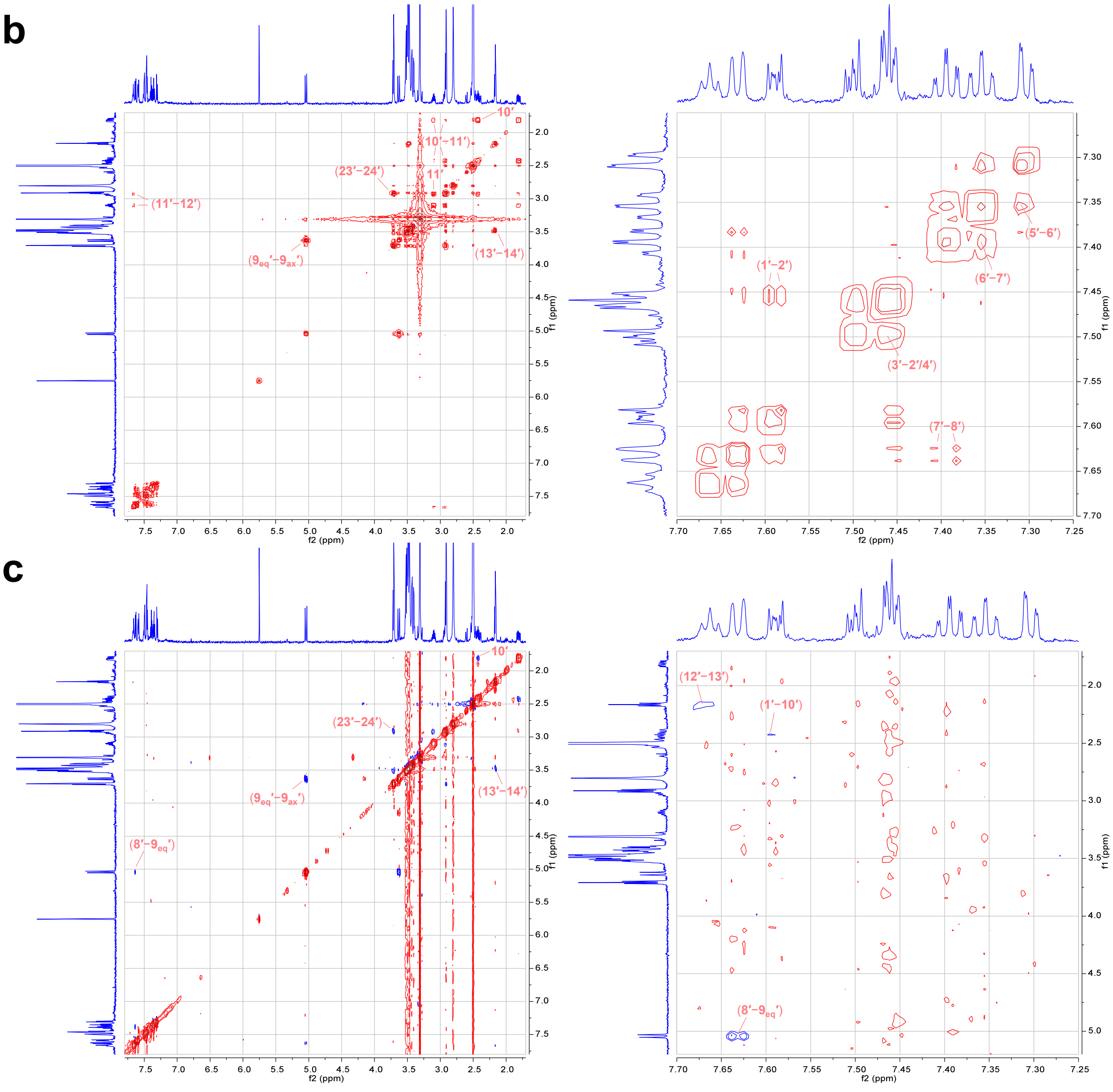

Fig. S10. (a) ^{1}H NMR, (b) COSY, and (c) NOESY spectra of DBCO-NHS ester in DMSO-d_6.

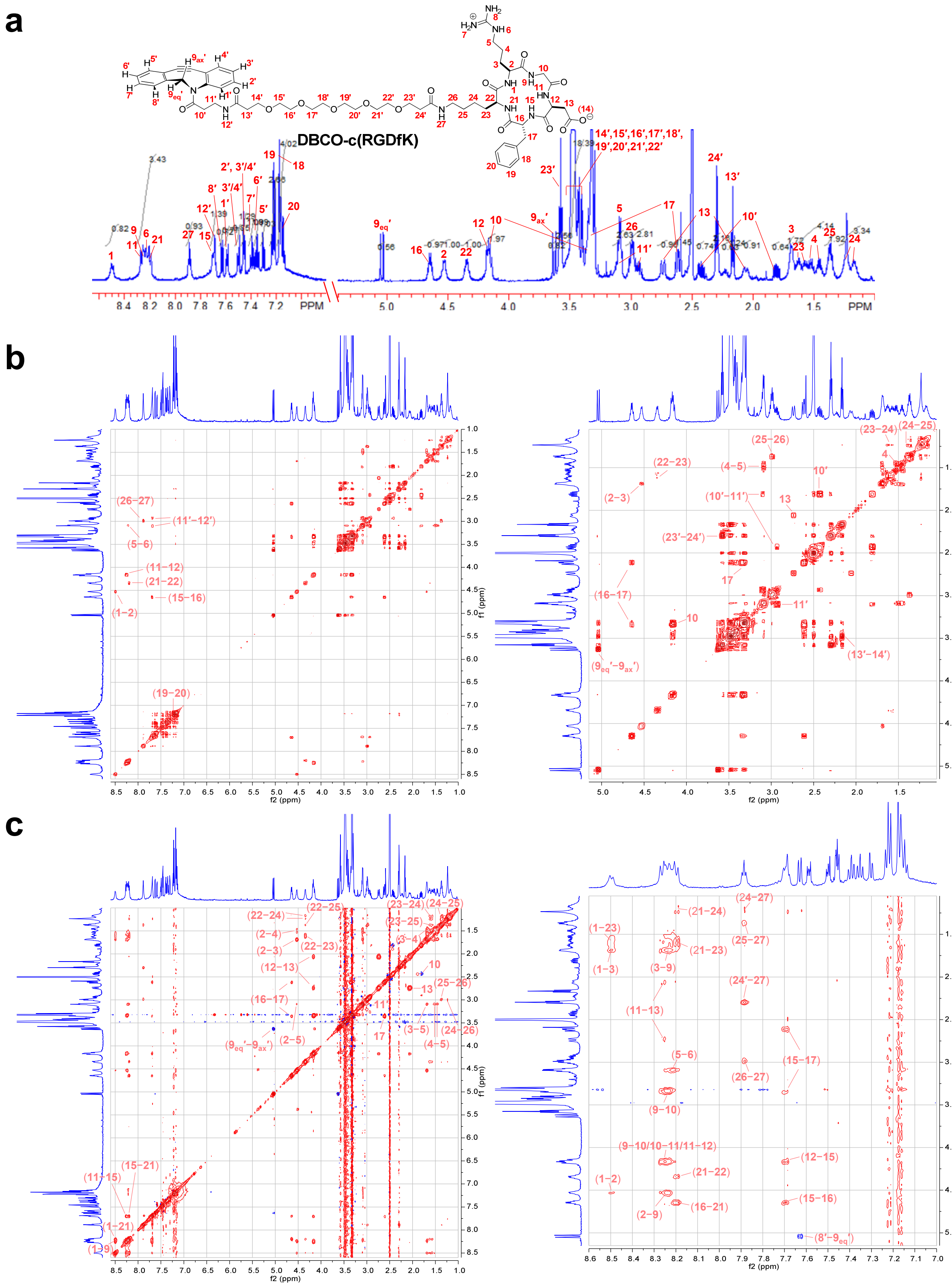

Fig. S11. (a) ^{1}H NMR, (b) COSY, and (c) NOESY spectra of **DBCO-c(RGDfK)** in DMSO-d_6.

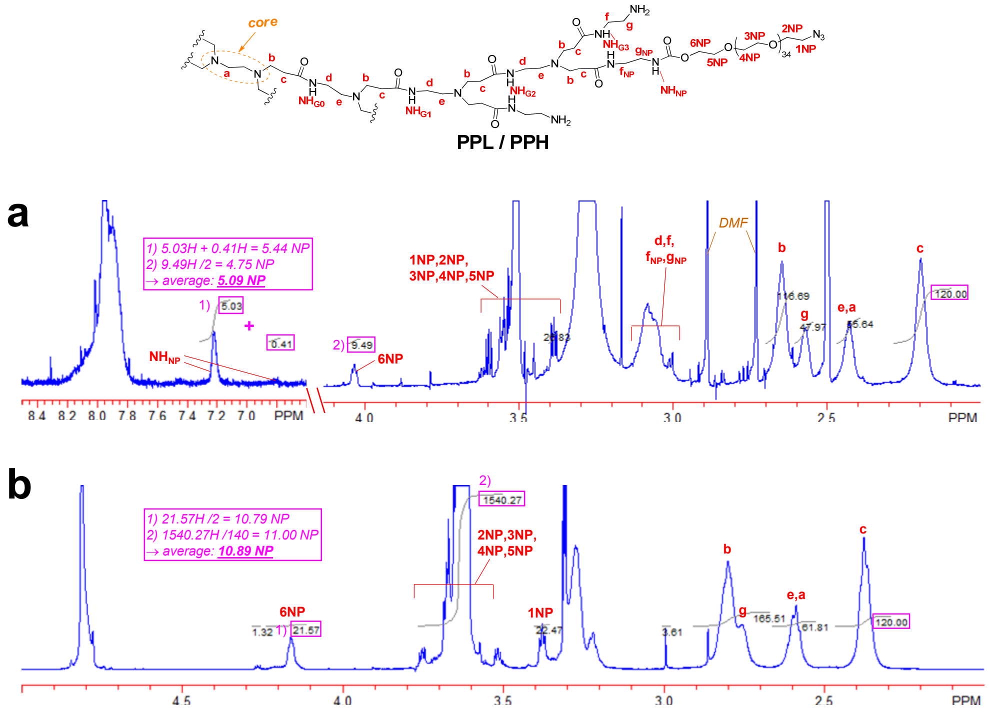

Fig. S12. ^{1}H NMR spectra of (a) **PPL** recorded in DMSO-d_6 and (b) **PPH** recorded in CD_3OD.

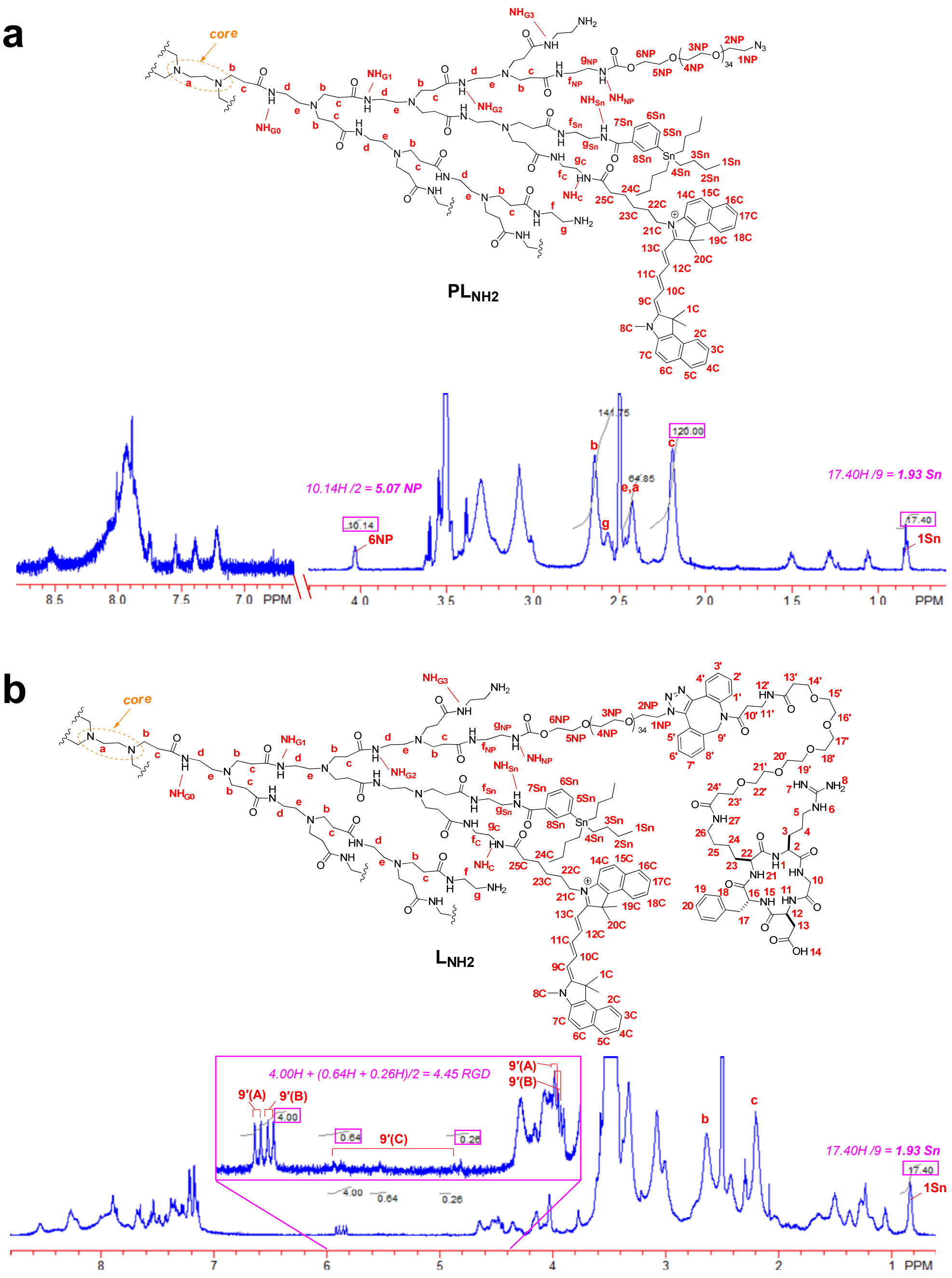

Fig. S13. ^{1}H NMR spectra of (a) $\mathbf{PL_{NH2}}$ and (b) $\mathbf{L_{NH2}}$ recorded in DMSO-d_6. For the peak assignment of three isomeric methylene protons ($H_{9'(A)}$, $H_{9'(B)}$, and $H_{9'(C)}$) of the DBCO moiety (inset), see Fig. S17.

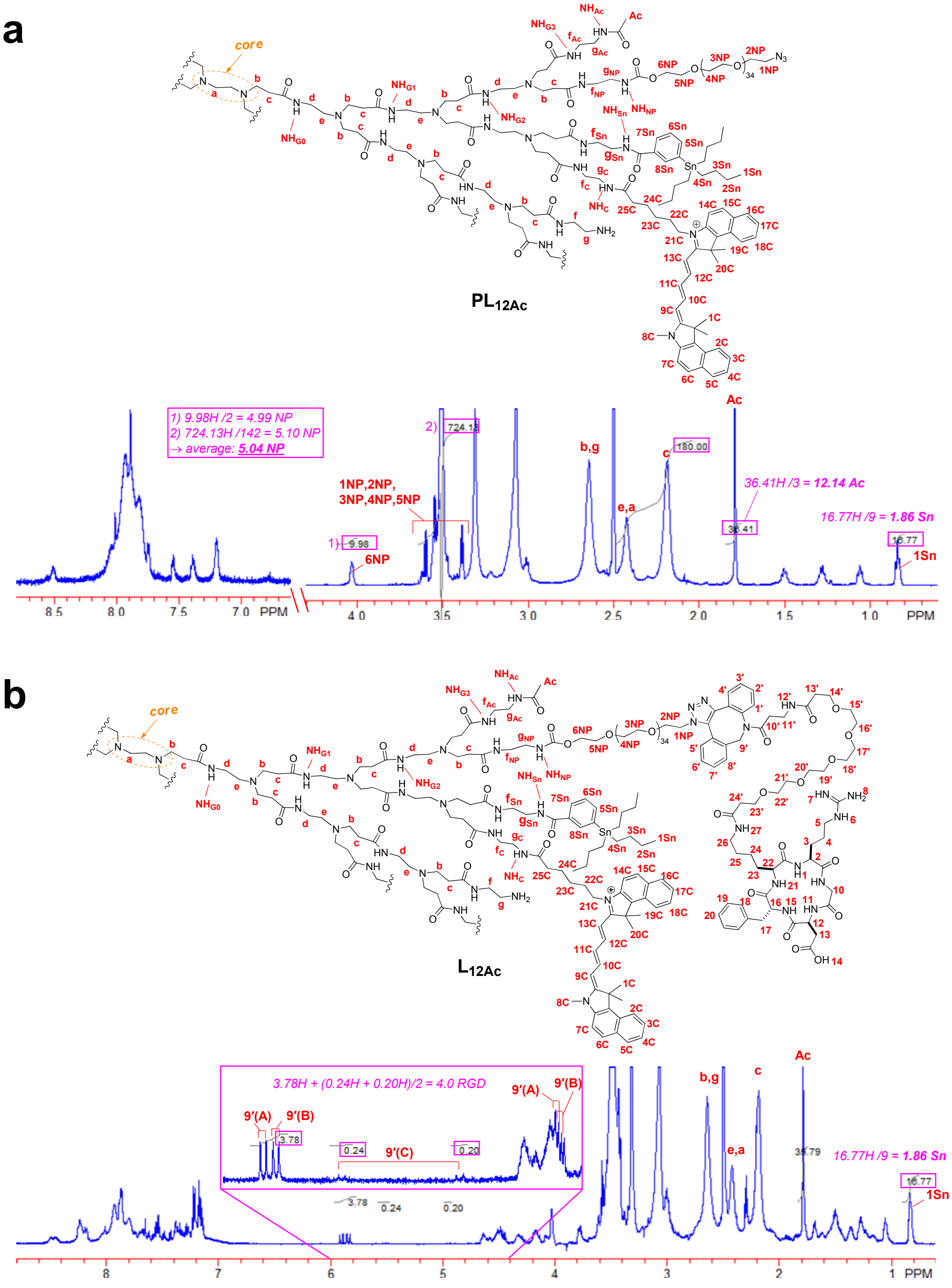

Fig. S14. ^{1}H NMR spectra of (a) **PL$_{12Ac}$** and (b) **L$_{12Ac}$** recorded in DMSO-d_6. For the peak assignment of three isomeric methylene protons ($H_{9'(A)}$, $H_{9'(B)}$, and $H_{9'(C)}$) of the DBCO moiety (inset), see Fig. S17.

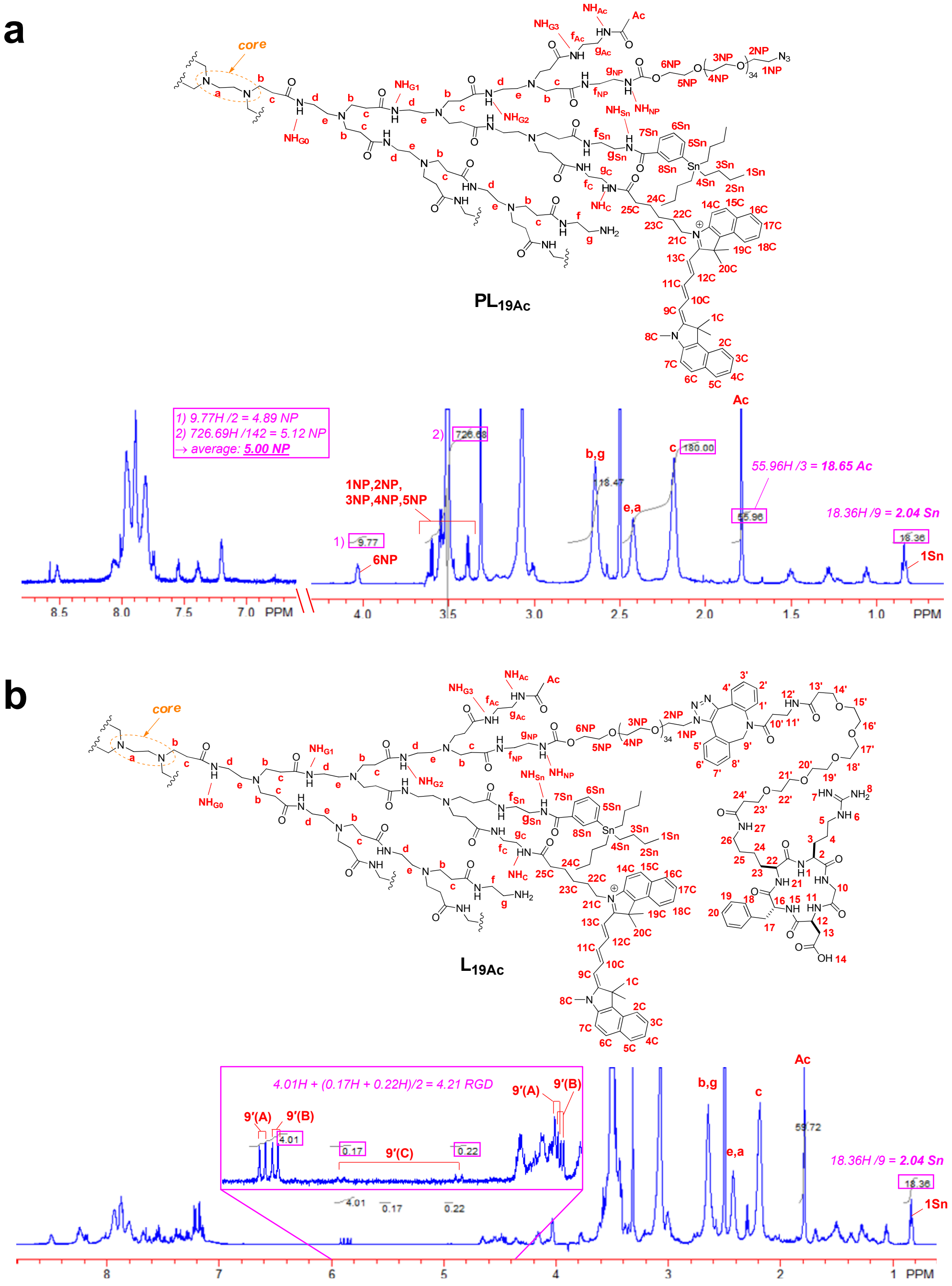

Fig. S15. ¹H NMR spectra of (a) **PL$_{19Ac}$** and (b) **L$_{19Ac}$** recorded in DMSO-*d*$_6$. For the peak assignment of three isomeric methylene protons (H$_{9'(A)}$, H$_{9'(B)}$, and H$_{9'(C)}$) of the DBCO moiety (inset), see Fig. S17.

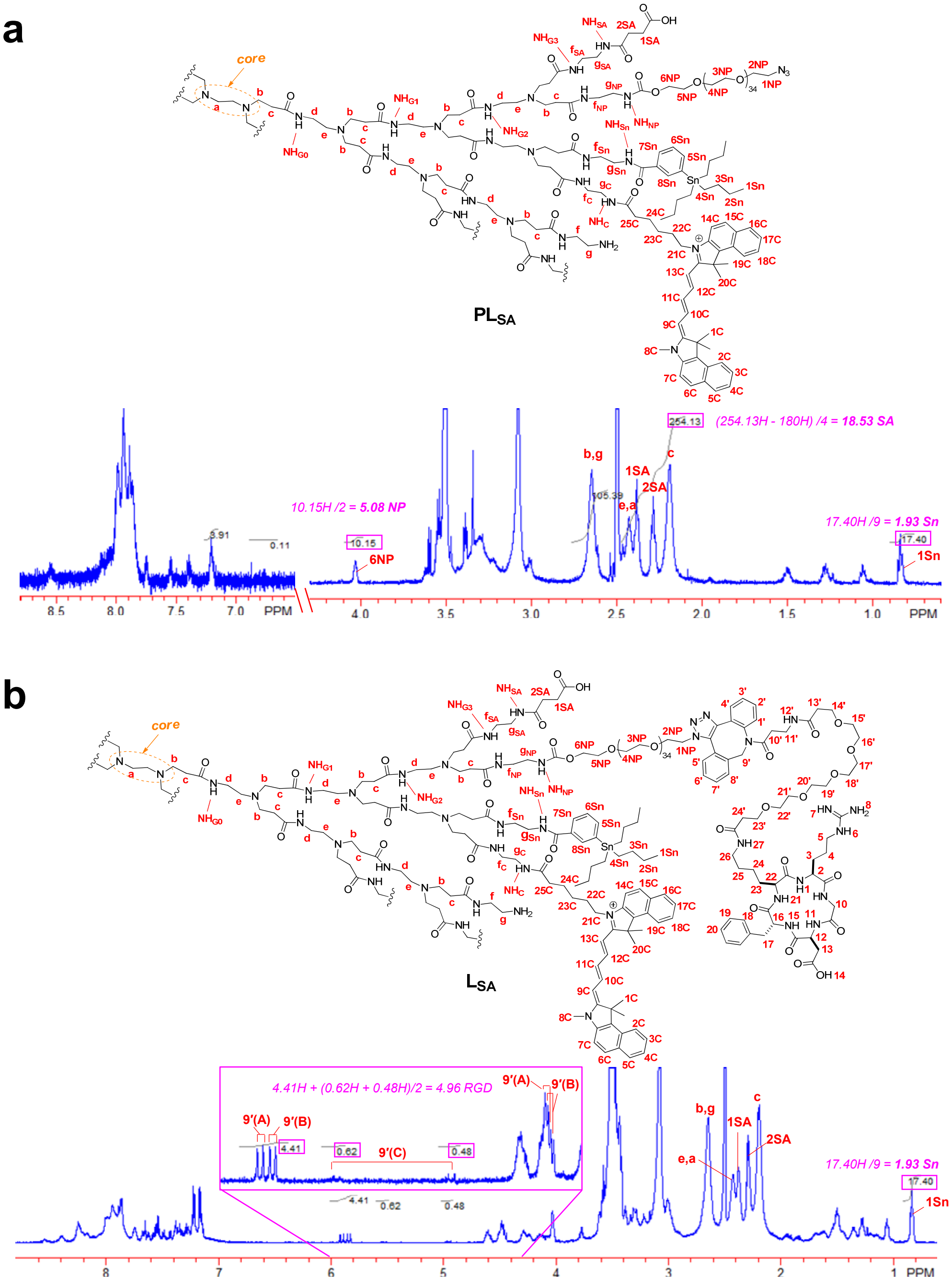

Fig. S16. ^{1}H NMR spectra of (a) **PL$_{SA}$** and (b) **L$_{SA}$** recorded in DMSO-d_6. For the peak assignment of three isomeric methylene protons ($H_{9'(A)}$, $H_{9'(B)}$, and $H_{9'(C)}$) of the DBCO moiety (inset), see Fig. S17.

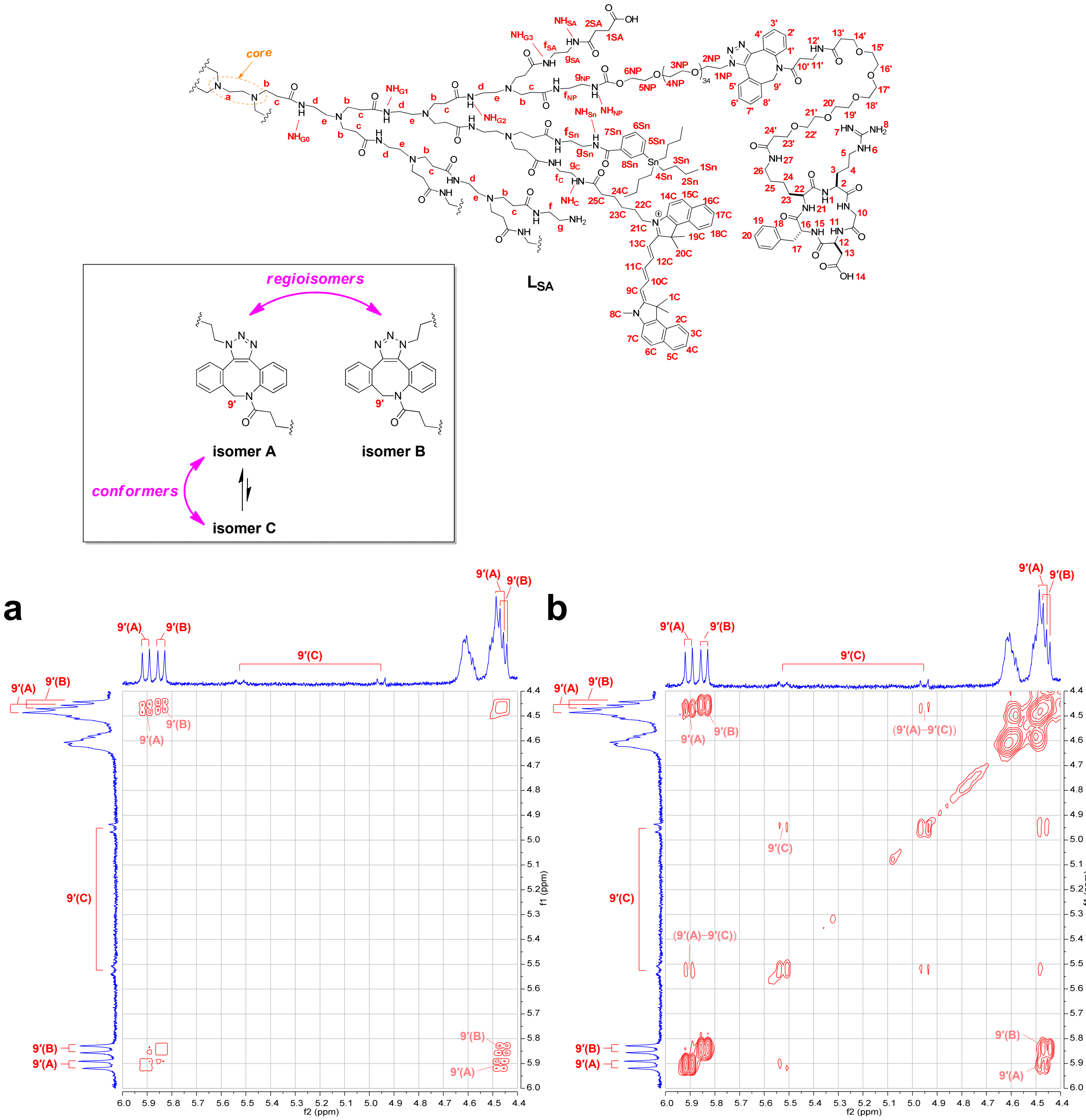

Fig. S17. (a) COSY and (b) NOESY spectra of $\mathbf{L_{SA}}$ recorded in DMSO-d_6. Regions containing the peaks corresponding to the methylene protons ($H_{9'}$) of the DBCO moiety are expanded to clearly distinguish the connectivity among three isomers (A, B, and C) found in the NMR spectra. Based on the NMR analysis, the integration values of three isomeric $H_{9'}$ peaks were combined to determine the total number of c(RGDfK) units attached to the dendrimer conjugate $\mathbf{L_{SA}}$.

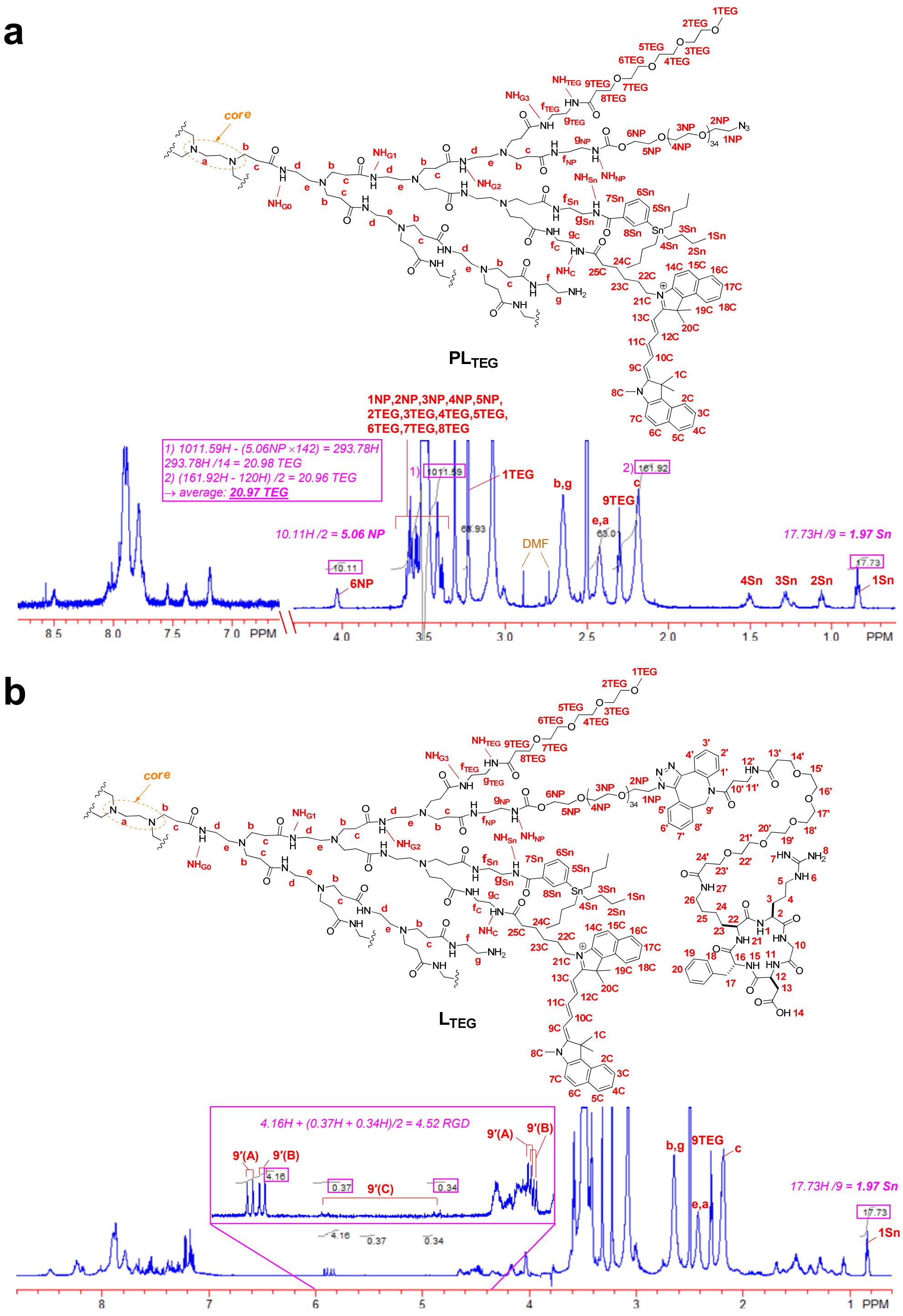

Fig. S18. ¹H NMR spectra of (a) **PL$_{TEG}$** and (b) **L$_{TEG}$** recorded in DMSO-d_6. For the peak assignment of three isomeric methylene protons ($H_{9'(A)}$, $H_{9'(B)}$, and $H_{9'(C)}$) of the DBCO moiety (inset), see Fig. S17.

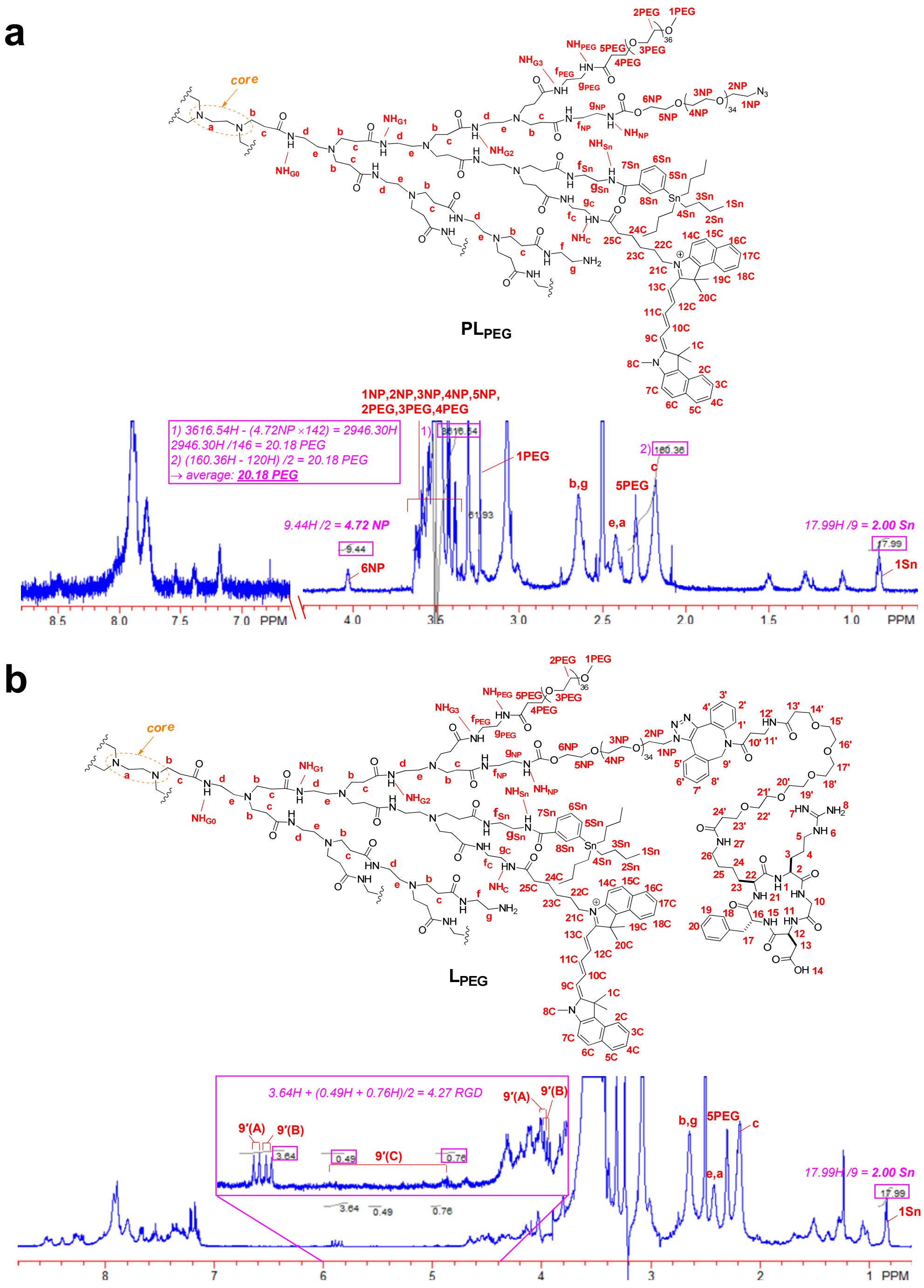

Fig. S19. ^{1}H NMR spectra of (a) **PL**$_{\mathbf{PEG}}$ and (b) **L**$_{\mathbf{PEG}}$ recorded in DMSO-d_6. For the peak assignment of three isomeric methylene protons ($H_{9'(A)}$, $H_{9'(B)}$, and $H_{9'(C)}$) of the DBCO moiety (inset), see Fig. S17.

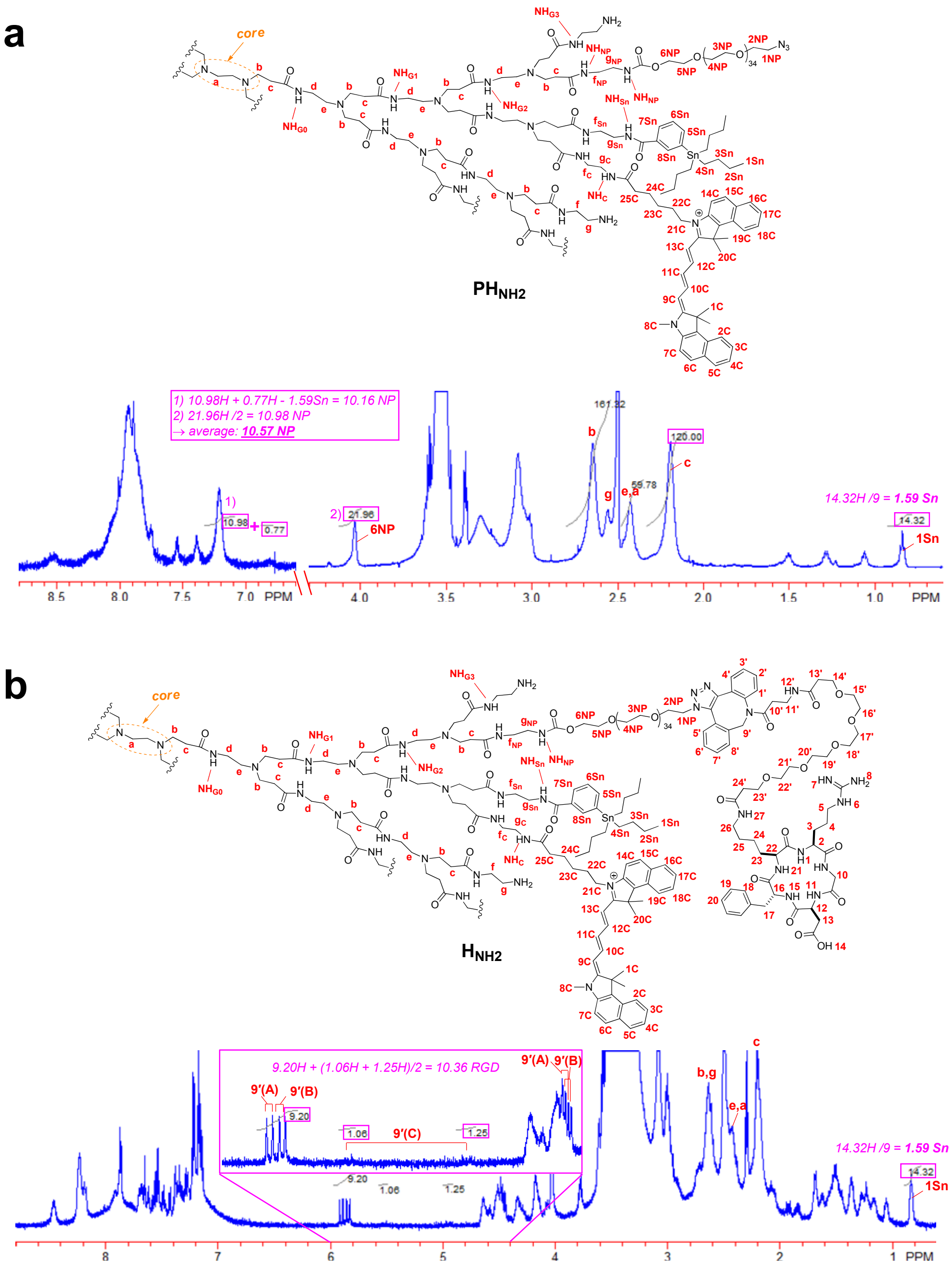

Fig. S20. ^{1}H NMR spectra of (a) $\mathbf{PH_{NH2}}$ and (b) $\mathbf{H_{NH2}}$ recorded in DMSO-d_6. For the peak assignment of three isomeric methylene protons ($H_{9'(A)}$, $H_{9'(B)}$, and $H_{9'(C)}$) of the DBCO moiety (inset), see Fig. S17.

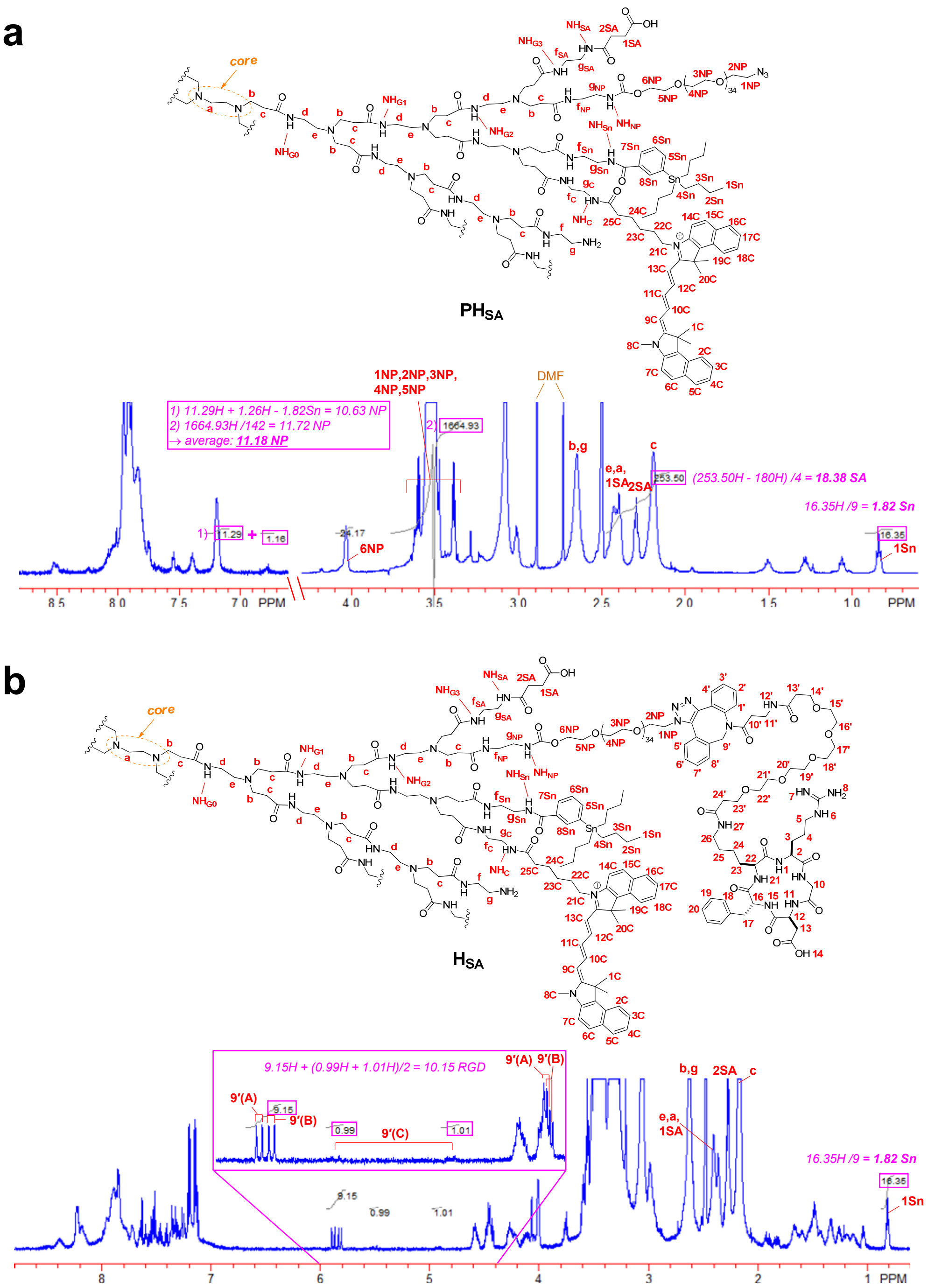

Fig. S21. ^{1}H NMR spectra of (a) **PH$_{SA}$** and (b) **H$_{SA}$** recorded in DMSO-d_6. For the peak assignment of three isomeric methylene protons ($H_{9'(A)}$, $H_{9'(B)}$, and $H_{9'(C)}$) of the DBCO moiety (inset), see Fig. S17.

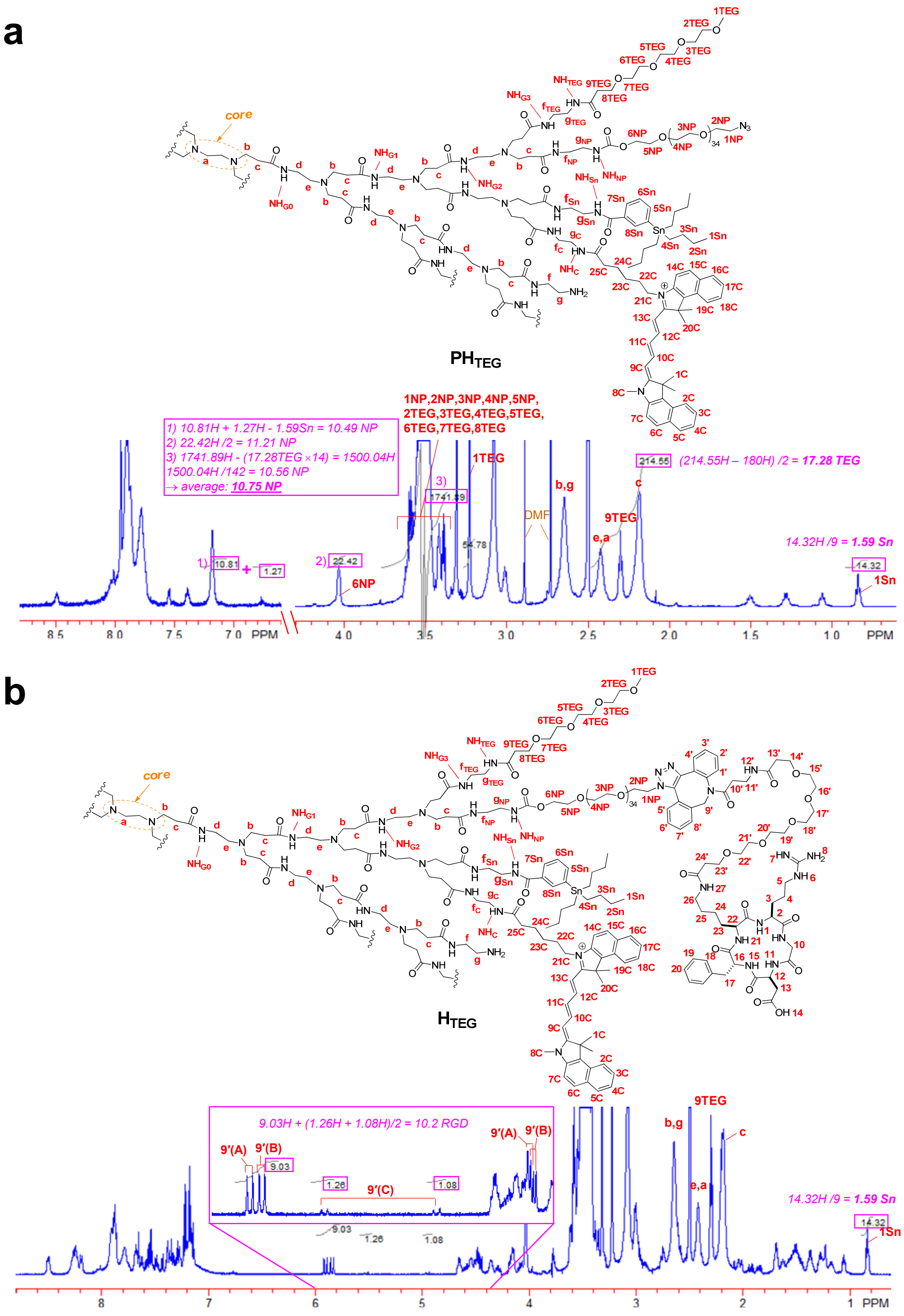

Fig. S22. ^{1}H NMR spectra of (a) $\mathbf{PH_{TEG}}$ and (b) $\mathbf{H_{TEG}}$ recorded in DMSO-d_6. For the peak assignment of three isomeric methylene protons ($H_{9'(A)}$, $H_{9'(B)}$, and $H_{9'(C)}$) of the DBCO moiety (inset), see Fig. S17.

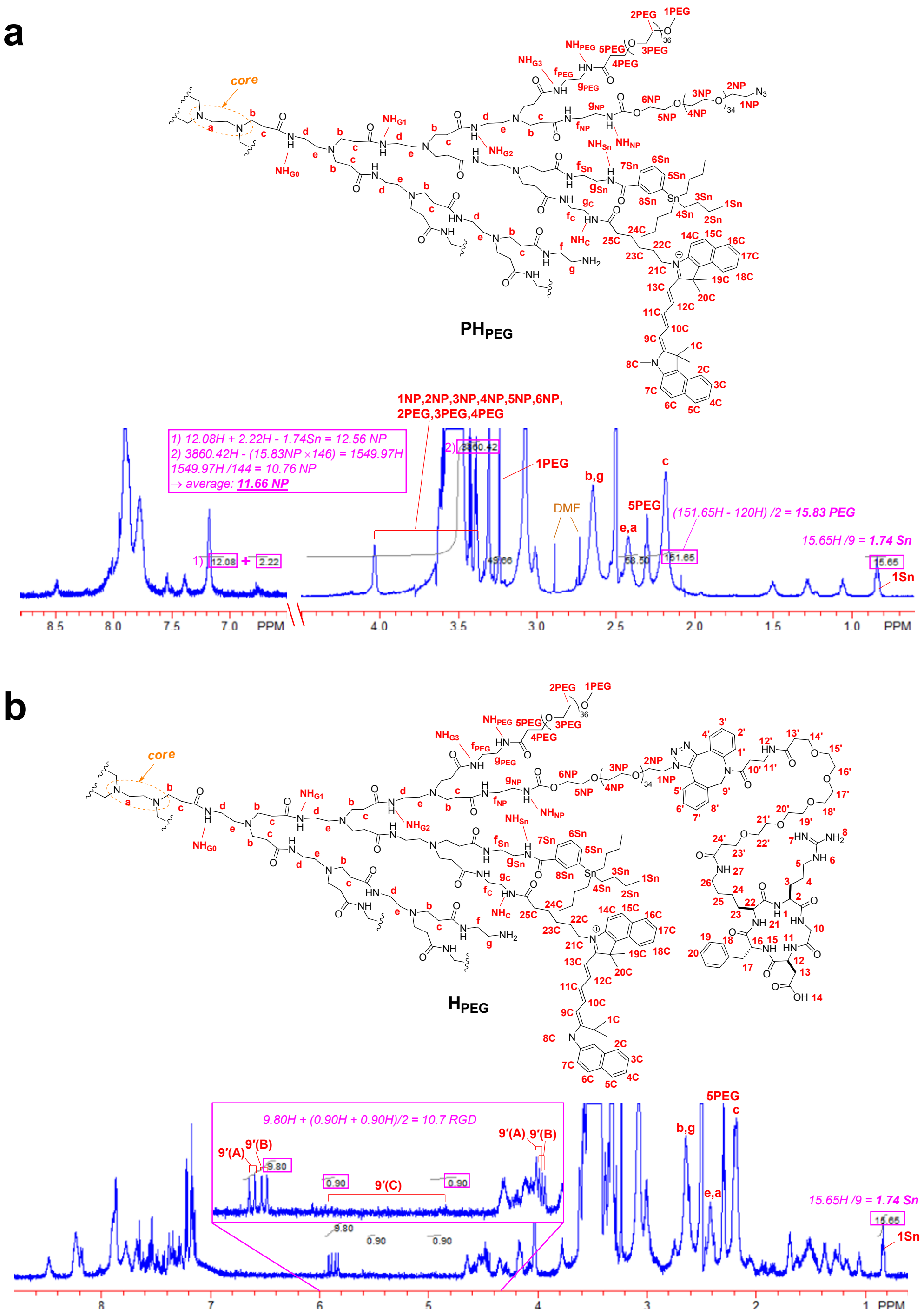

Fig. S23. ^{1}H NMR spectra of (a) **PH$_{PEG}$** and (b) **H$_{PEG}$** recorded in DMSO-d_6. For the peak assignment of three isomeric methylene protons ($H_{9'(A)}$, $H_{9'(B)}$, and $H_{9'(C)}$) of the DBCO moiety (inset), see Fig. S17.

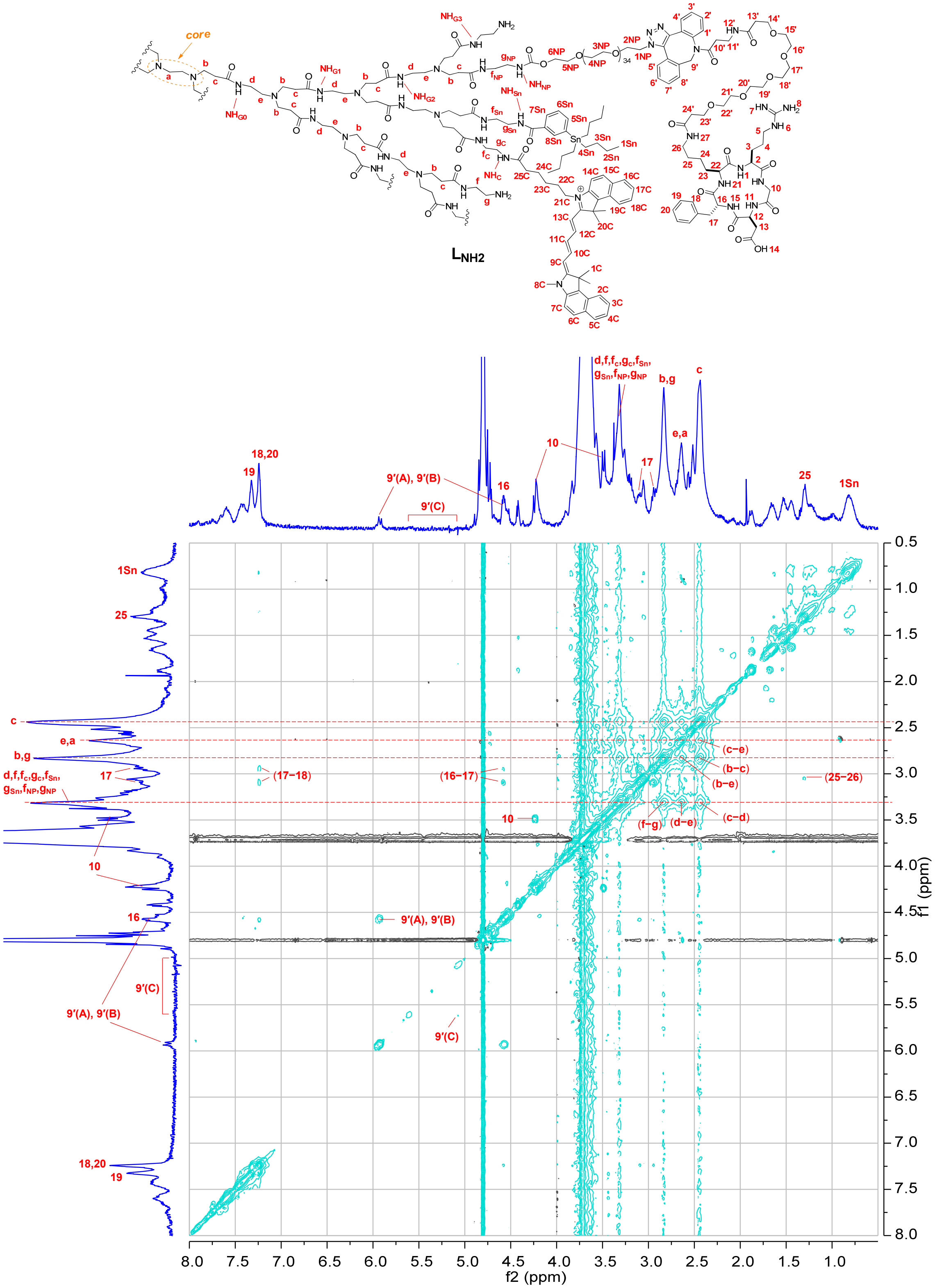

Fig. S24. NOESY spectrum of $\mathbf{L_{NH2}}$ recorded in D_2O. The peak assignment of three isomeric methylene protons ($H_{9'(A)}$, $H_{9'(B)}$, and $H_{9'(C)}$) of the DBCO moiety was done similarly as described in Fig. S17.

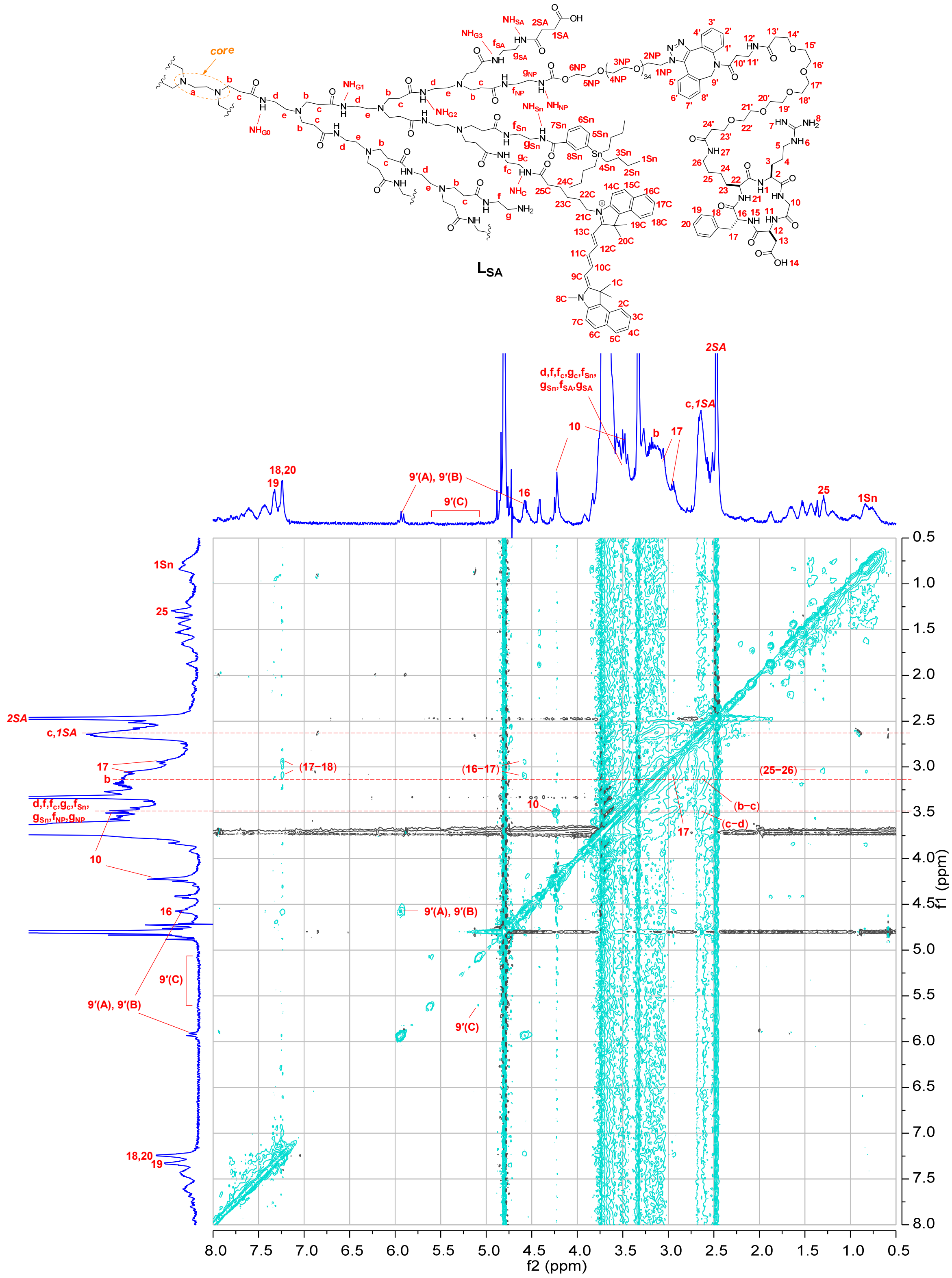

Fig. S25. NOESY spectrum of $\mathbf{L_{SA}}$ recorded in D_2O. Some tentative peak assignments are shown in italics. The peak assignment of three isomeric methylene protons ($H_{9'(A)}$, $H_{9'(B)}$, and $H_{9'(C)}$) of the DBCO moiety was done similarly as described in Fig. S17.

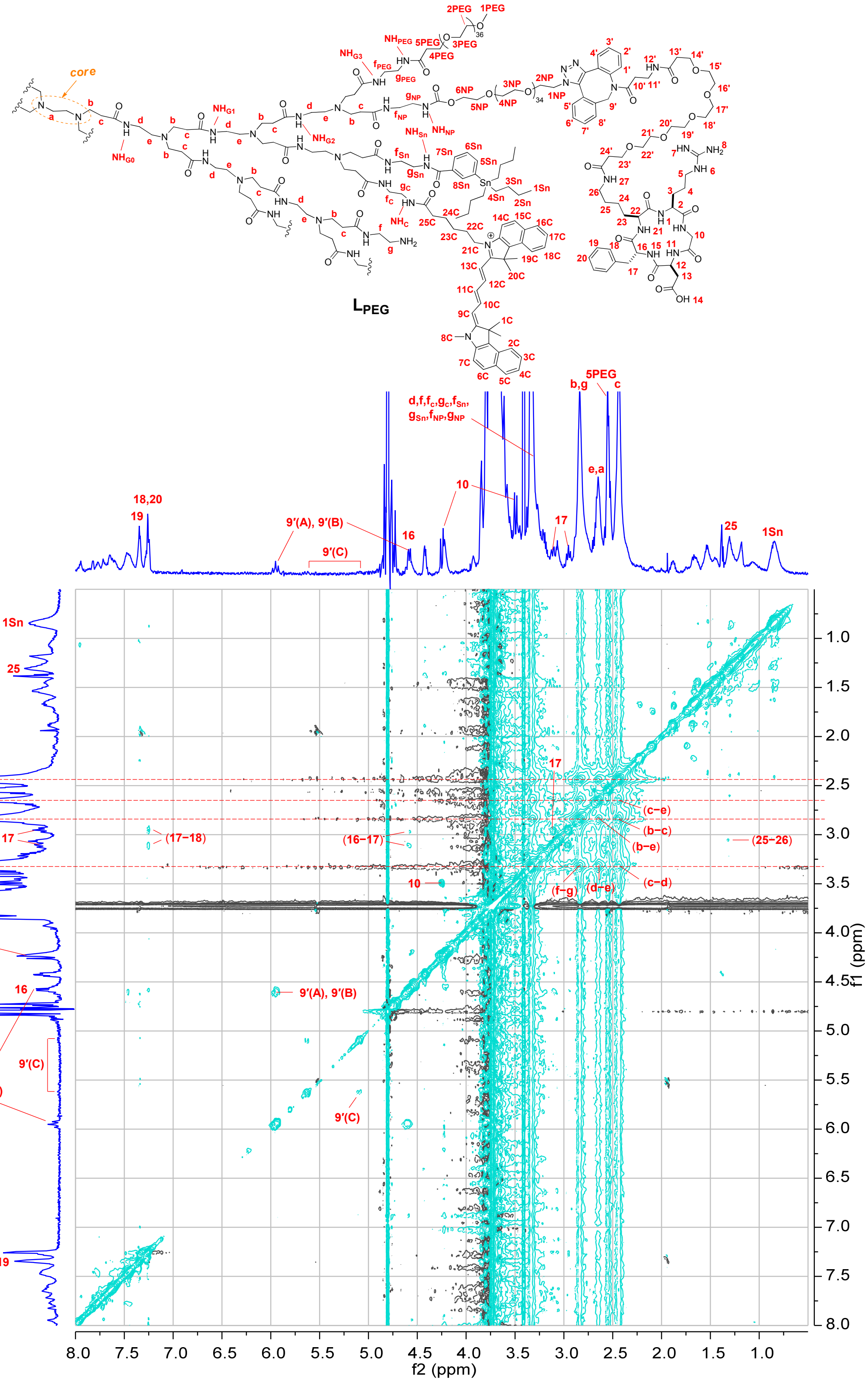

Fig. S26. NOESY spectrum of **L_{PEG}** recorded in D_2O. The peak assignment of three isomeric methylene protons ($H_{9'(A)}$, $H_{9'(B)}$, and $H_{9'(C)}$) of the DBCO moiety was done similarly as described in Fig. S17.

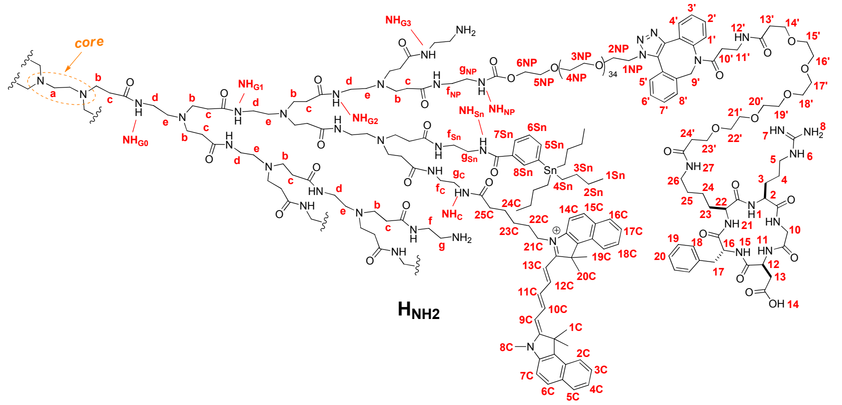

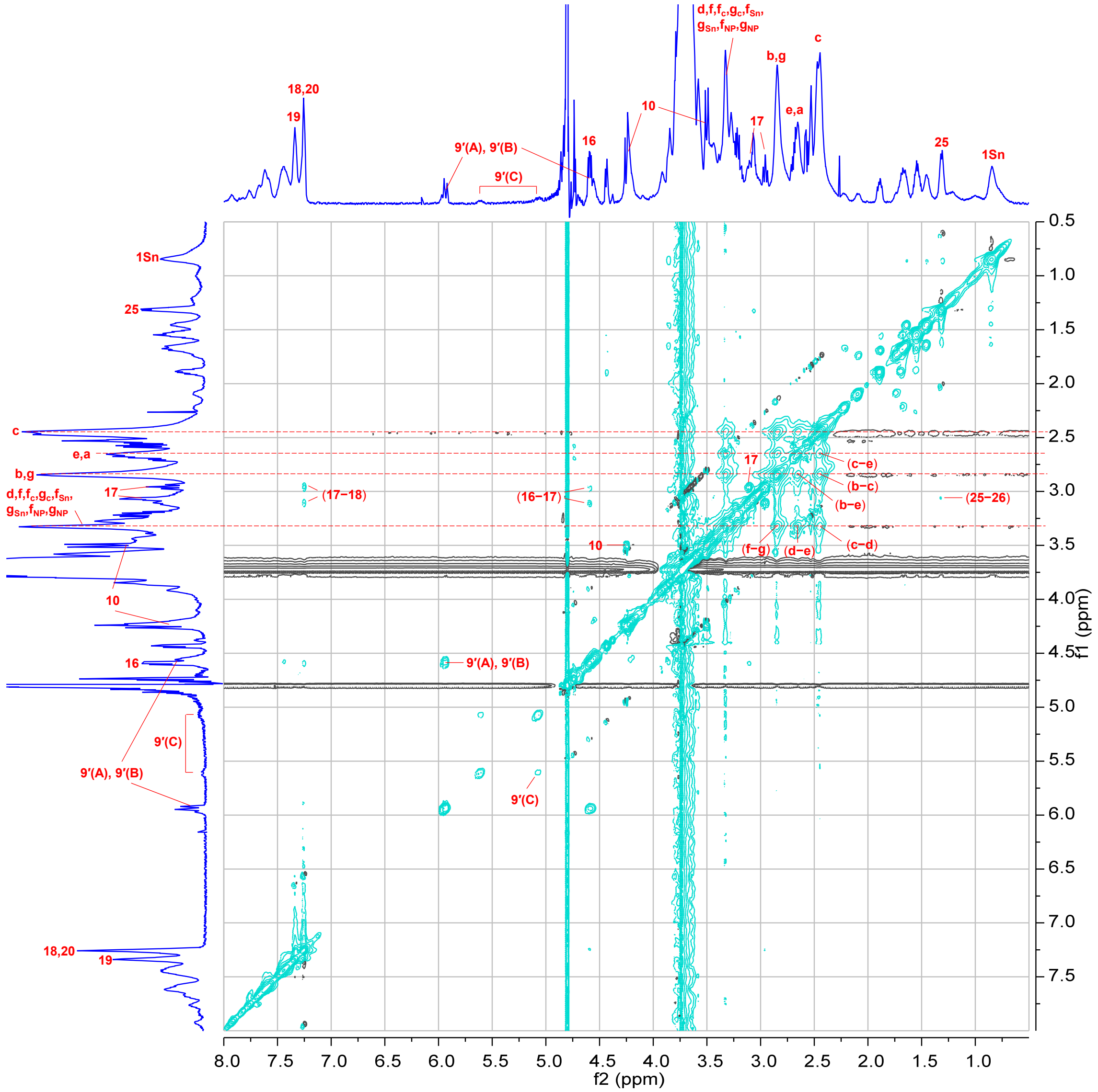

Fig. S27. NOESY spectrum of $\mathbf{H_{NH2}}$ recorded in D_2O. The peak assignment of three isomeric methylene protons ($H_{9'(A)}$, $H_{9'(B)}$, and $H_{9'(C)}$) of the DBCO moiety was done similarly as described in Fig. S17.

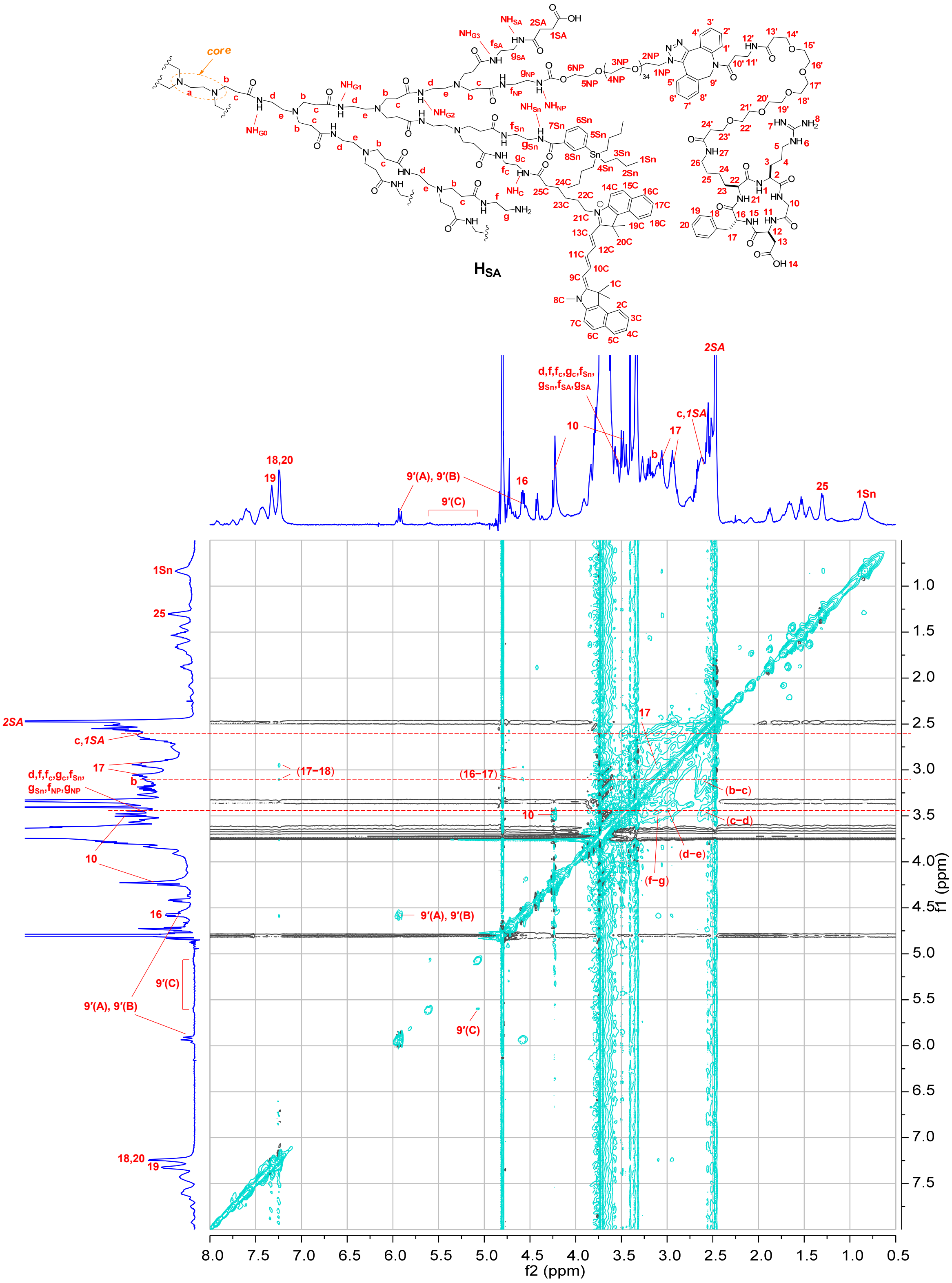

Fig. S28. NOESY spectrum of $\mathbf{H_{SA}}$ recorded in D_2O. Some tentative peak assignments are shown in italics. The peak assignment of three isomeric methylene protons ($H_{9'(A)}$, $H_{9'(B)}$, and $H_{9'(C)}$) of the DBCO moiety was done similarly as described in Fig. S17.

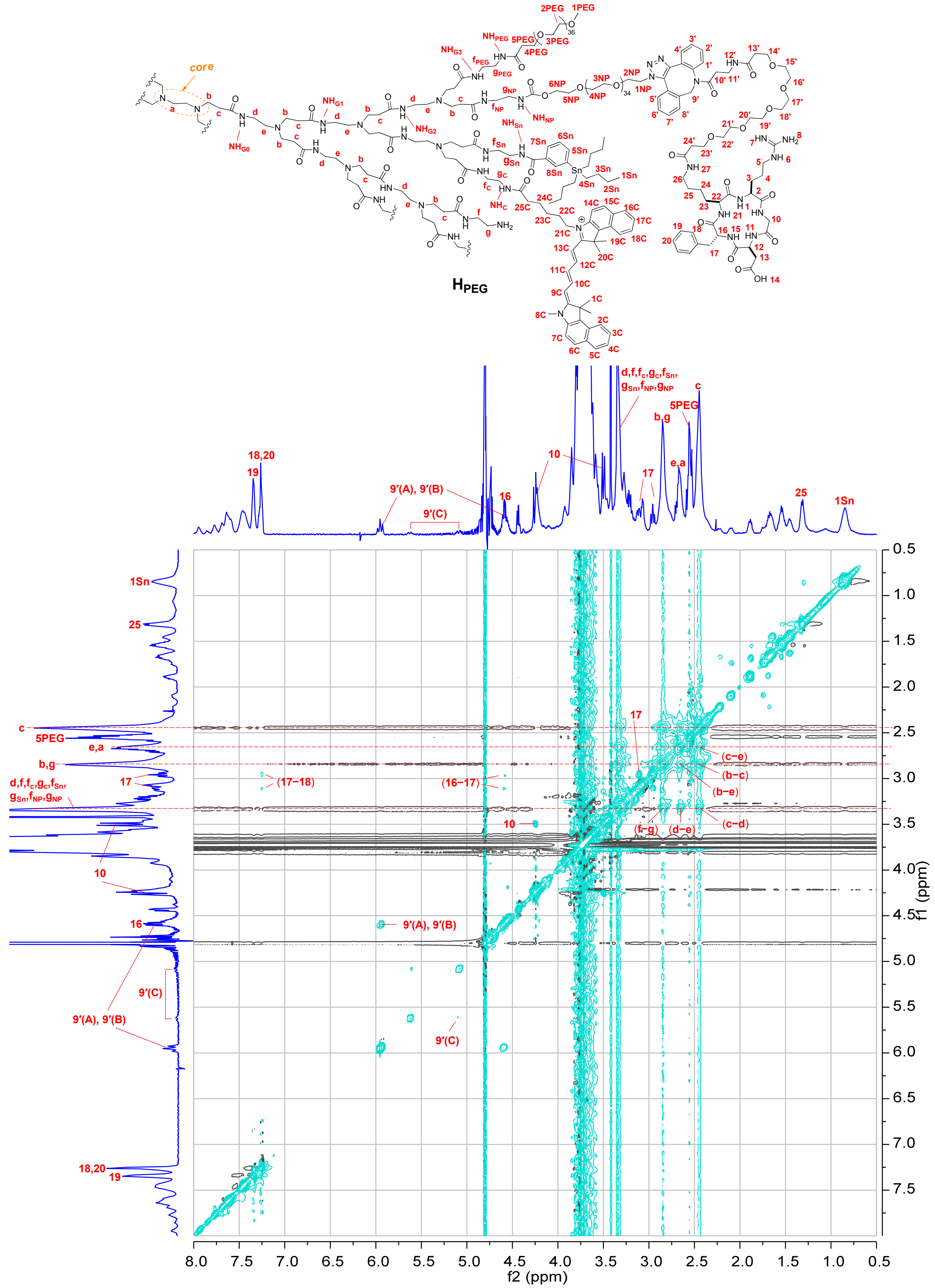

Fig. S29. NOESY spectrum of $\mathbf{H_{PEG}}$ recorded in D_2O. The peak assignment of three isomeric methylene protons ($H_{9'(A)}$, $H_{9'(B)}$, and $H_{9'(C)}$) of the DBCO moiety was done similarly as described in Fig. S17.

a

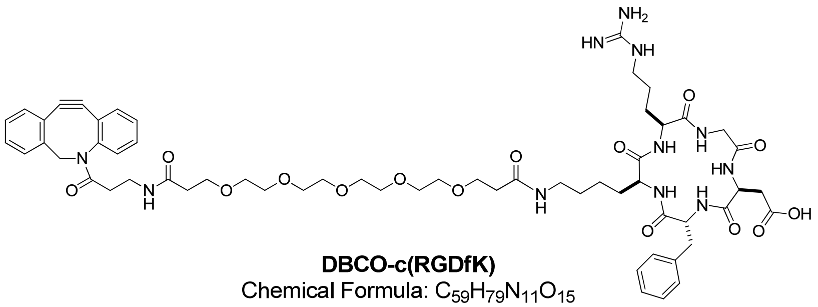

DBCO-c(RGDfK)
Chemical Formula: $C_{59}H_{79}N_{11}O_{15}$
Exact Mass: 1181.5757
Molecular Weight: 1182.3430
Elemental Analysis: C, 59.94; H, 6.74; N, 13.03; O, 20.30

Ju Young Heo, jy1074 University of Illnois, SCS, Mass Spectrometry Lab Q-tof UE521
Qtof_44231 42 (1.757) AM (Cen,3, 80.00, Ar,15000.0,716.46,0.70,LS 3); Sm (SG, 2x3.00); Cm (39:42) 1: TOF MS ES+
4.81e+003

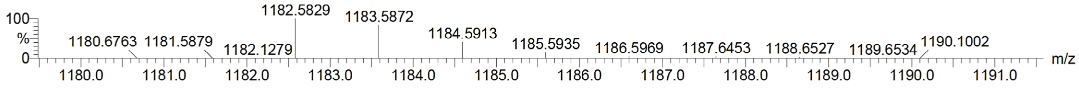

Minimum:				-1.5		
Maximum:		5.0	10.0	600.0		
Mass	Calc. Mass	mDa	PPM	DBE	i-FIT	Formula
1182.5829	1182.5835	-0.6	-0.5	25.5	89.2	C59 H80 N11 O15
	1182.5894	-6.5	-5.5	16.5	186.1	C52 H84 N11 O20

b

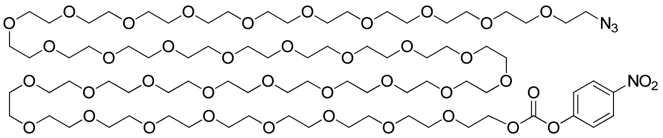

***p*NP-PEG-N$_3$**
Chemical Formula: $C_{79}H_{148}N_4O_{40}$
Exact Mass: 1792.9670
Molecular Weight: 1794.0410
Elemental Analysis: C, 52.89; H, 8.32; N, 3.12; O, 35.67

Ju Young Heo, jy1066-f8_NaCl University of Illnois, SCS, Mass Spectrometry Lab Q-tof UE521
Qtof_44229A 54 (2.257) AM (Cen,3, 80.00, Ar,15000.0,716.46,0.70,LS 3); Sm (SG, 2x5.00); Cm (54:62) 1: TOF MS ES+
4.88e+003

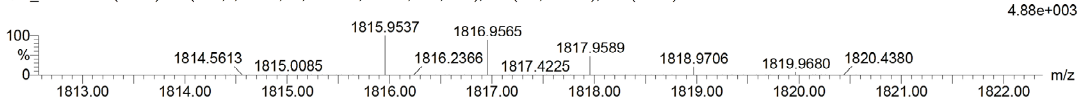

Minimum:				-1.5		
Maximum:		5.0	10.0	600.0		
Mass	Calc. Mass	mDa	PPM	DBE	i-FIT	Formula
1815.9537	1815.9568	-3.1	-1.7	7.5	5.4	C79 H148 N4 O40 Na
	1815.9592	-5.5	-3.0	10.5	12.5	C81 H147 N4 O40
	1815.9426	11.1	6.1	82.5	48.8	C88 H18 N5 O42
	1815.9402	13.5	7.4	79.5	35.1	C86 H19 N5 O42 Na

Fig. S30. ESI HRMS results of (a) **DBCO-c(RGDfK)** and (b) ***p*NP-PEG-N$_3$**.

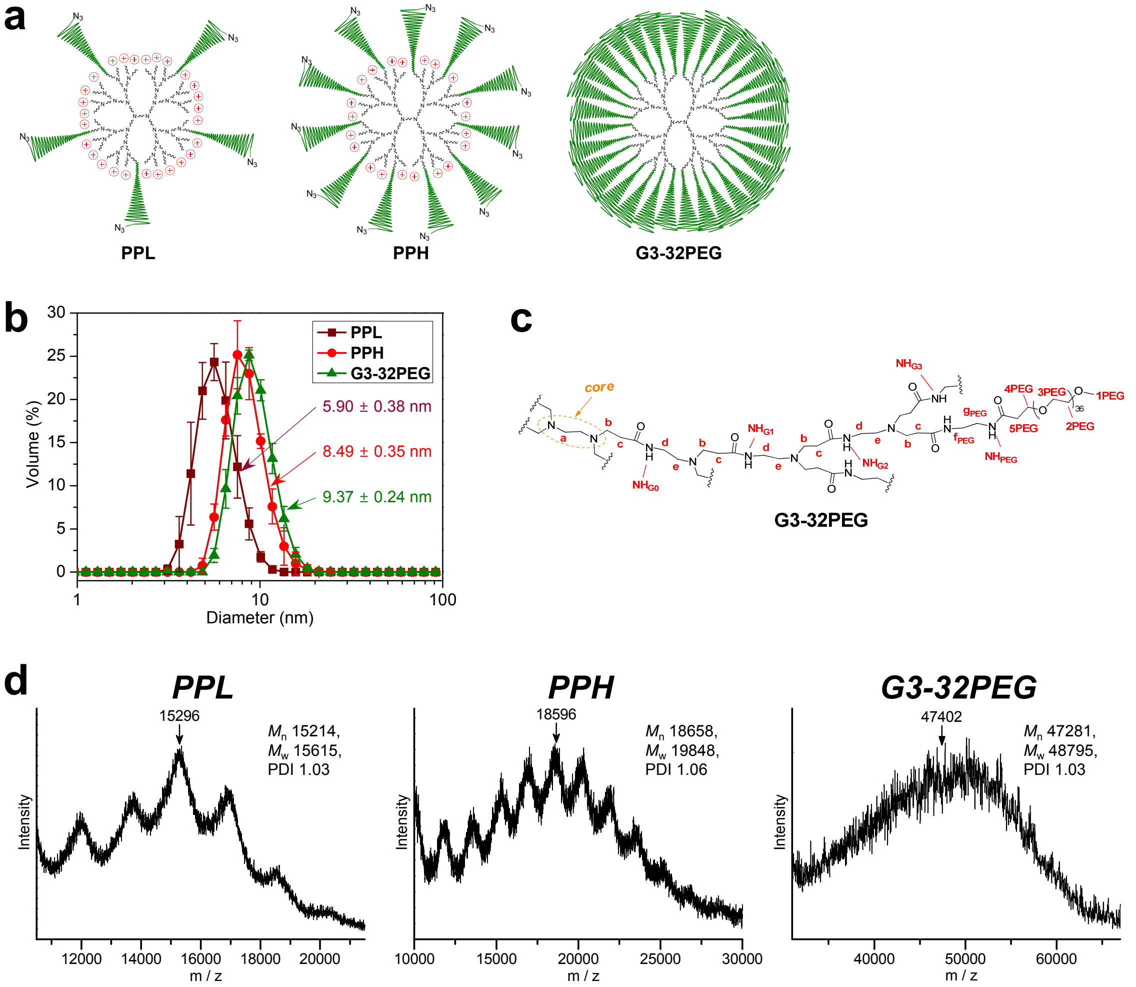

Fig. S31. Characterization of synthetic intermediates, **PPL** and **PPH**, and a fully PEGylated G3 PAMAM dendrimer, **G3-32PEG**. **G3-32PEG** was prepared as a control using mPEG-NHS ester (**11**, see Fig. S5) in order to roughly estimate the hydrodynamic diameter of fully PEGylated compounds, such as **PL$_{PEG}$** and **PH$_{PEG}$**, by dynamic light scattering (DLS) (see Experimental Section in the main text for details). (a) Schematic illustrations; (b) size distribution plots obtained by DLS at a sample concentration of 2 mg/mL in 10 mM NaCl solution (pH 7.4) at 25 °C; (c) the proton labeling method of **G3-32PEG** for the peak assignment of its ^{1}H NMR spectrum; (d) MALDI-TOF mass spectra using DHB as a matrix. The peak-average molar mass (M_p) values were assigned (arrow) approximately at the center of the bandwidth at the half-height of each peak.

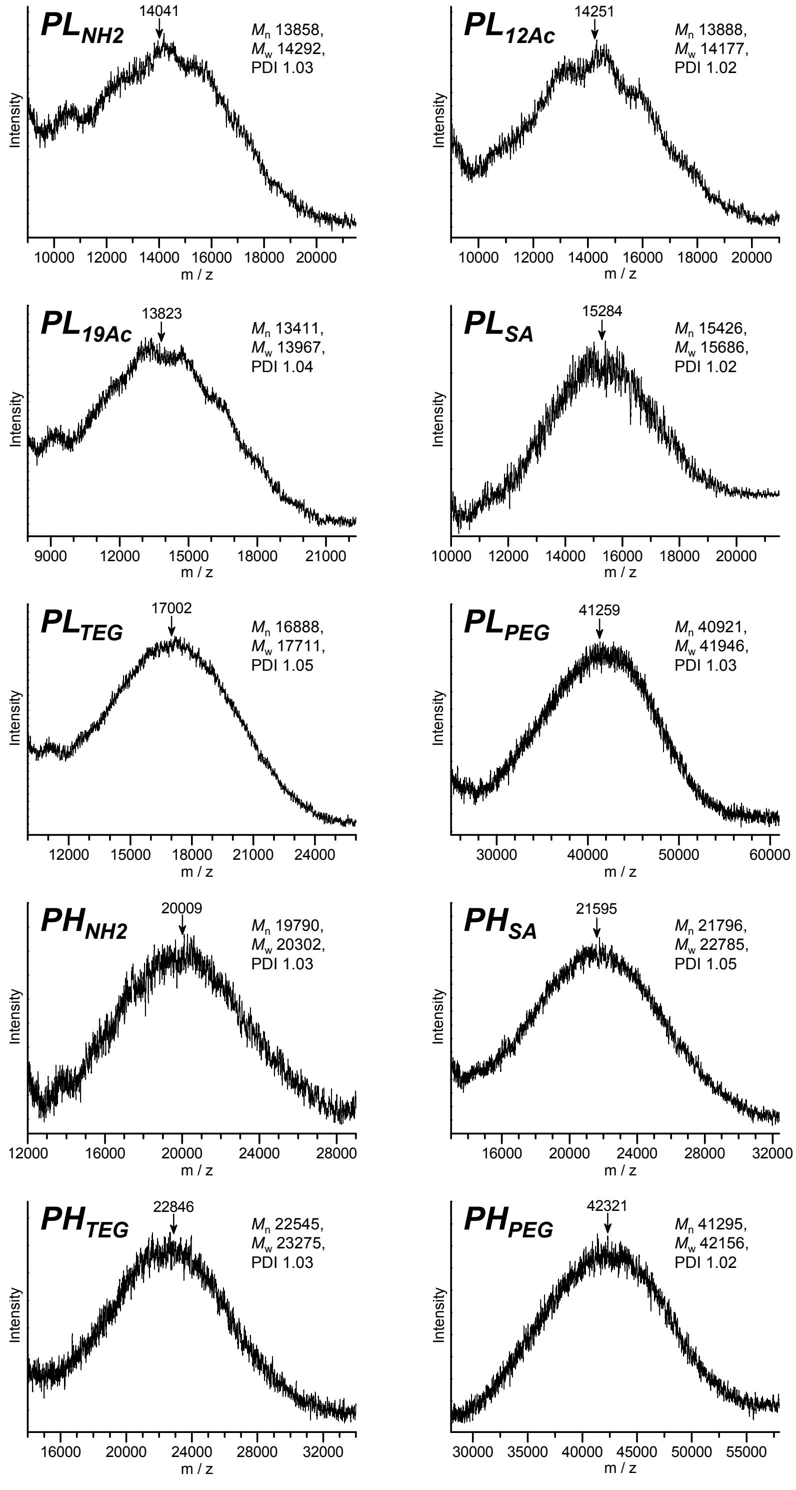

Fig. S32. MALDI-TOF mass spectra of 10 untargeted agents using DHB as a matrix. M_p values were assigned (arrow) approximately at the center of the bandwidth at the half-height of each peak. The number-average molar mass (M_n), weight-average molar mass (M_w), and polydispersity index (PDI, M_w/M_n) were calculated within the mass range shown here (*i.e.*, calculated region; see Experimental Section) using the Data Explorer software.

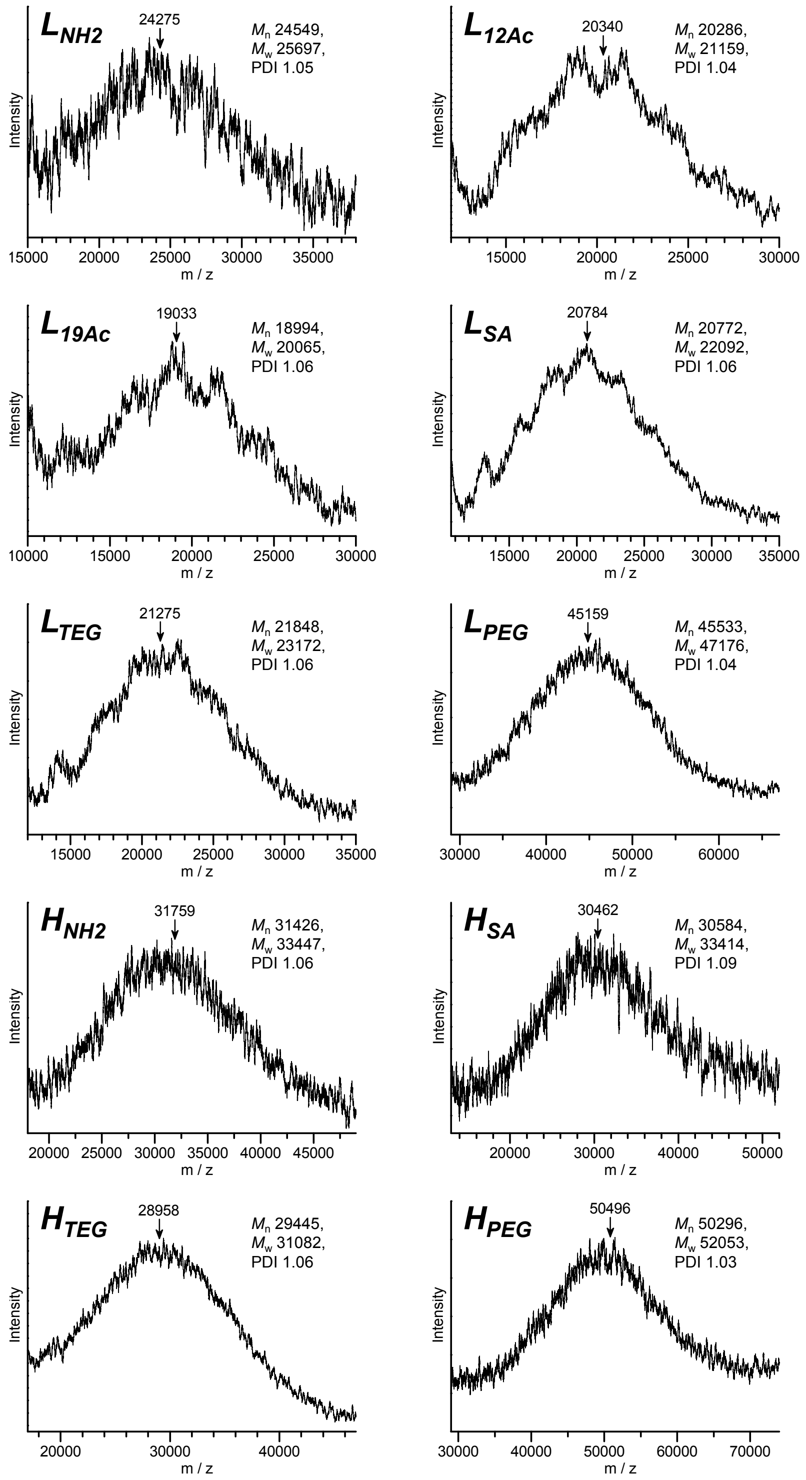

Fig. S33. MALDI-TOF mass spectra of 10 targeted agents using DHB as a matrix. M_p values were assigned (arrow) approximately at the center of the bandwidth at the half-height of each peak. M_n, M_w, and PDI (M_w/M_n) were calculated within the mass range shown here (*i.e.*, calculated region; see Experimental Section) using the Data Explorer software.

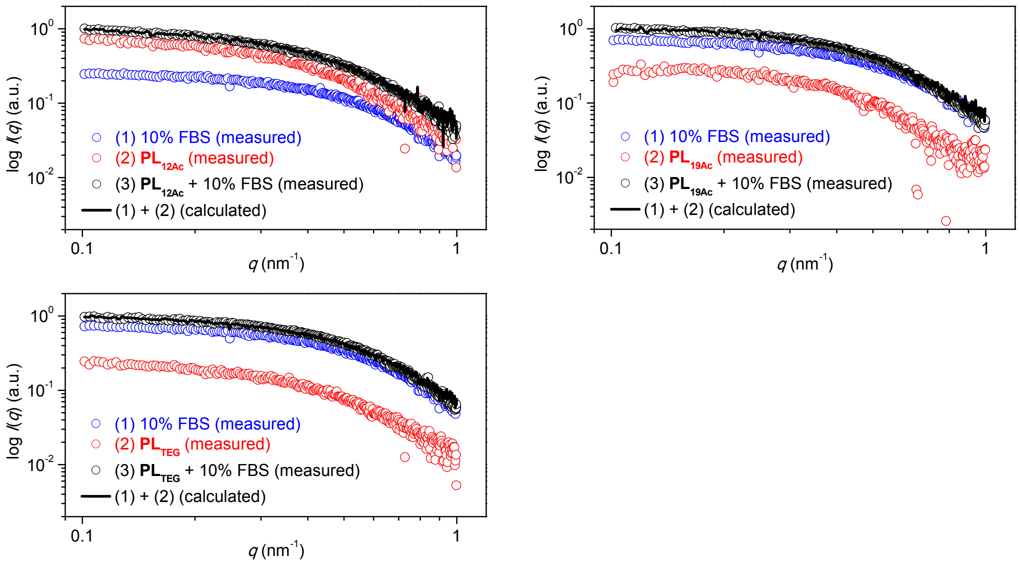

Fig. S34. Size (radius) of some untargeted agents (450 μM) estimated by SAXS at 25 °C in 2.5 mM NaCl solution (pH 7.4) including 10% (v/v) fetal bovine serum (FBS). The open symbols, (1)-(3), are experimentally obtained data. The SAXS profile of $\mathbf{PL_X}$ determined as such (3) coincided well with the linear sum (black line) of the individual profiles of (1) FBS and (2) $\mathbf{PL_X}$ (Fig. 2b), suggesting that these untargeted agents remained stable by retaining their original sizes in the media containing 10% FBS. The average $R_{g,\mathrm{G}}$ (radius of gyration) of the particles (*e.g.*, bovine serum albumin (BSA), $R_{g,\mathrm{G}}$ = 3.17 ± 0.05 nm) in FBS, 3.18 ± 0.04 nm (mean ± standard deviation (SD)), was estimated from the slope of the linear scattering data in the q^2-region using Guinier analysis. Data were not obtained for other untargeted agents due to the limited supply of samples for this study.

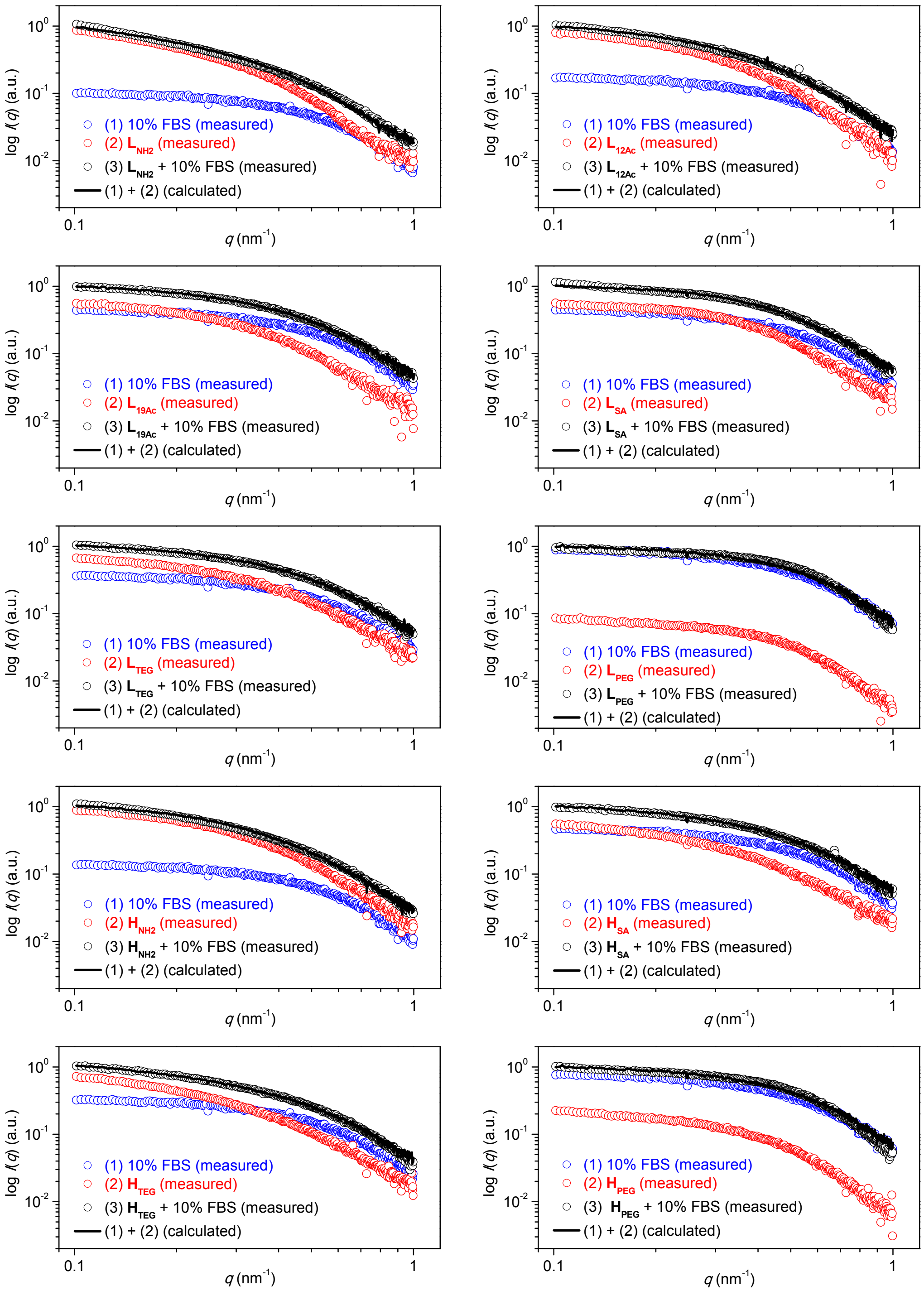

Fig. S35. Size (radius) of the targeted agents (**L_X** and **H_X**; 450 μM) estimated by SAXS at 25 °C in 2.5 mM NaCl solution (pH 7.4) including 10% (v/v) FBS. The open symbols, (1)-(3), are experimentally obtained data. The SAXS profile of (3) **L_X/H_X** determined as such coincided well with the linear sum (black line) of the individual profiles of (1) FBS and (2) **L_X/H_X** (Fig. 2a), suggesting that these compounds remained stable by retaining their original sizes in the media containing 10% FBS. The average $R_{g,G}$ (radius of gyration) of the particles (*e.g.*, BSA) in FBS, 3.18 ± 0.04 nm (mean ± SD), was estimated from the slope of the linear scattering data in the q^2-region using Guinier analysis.

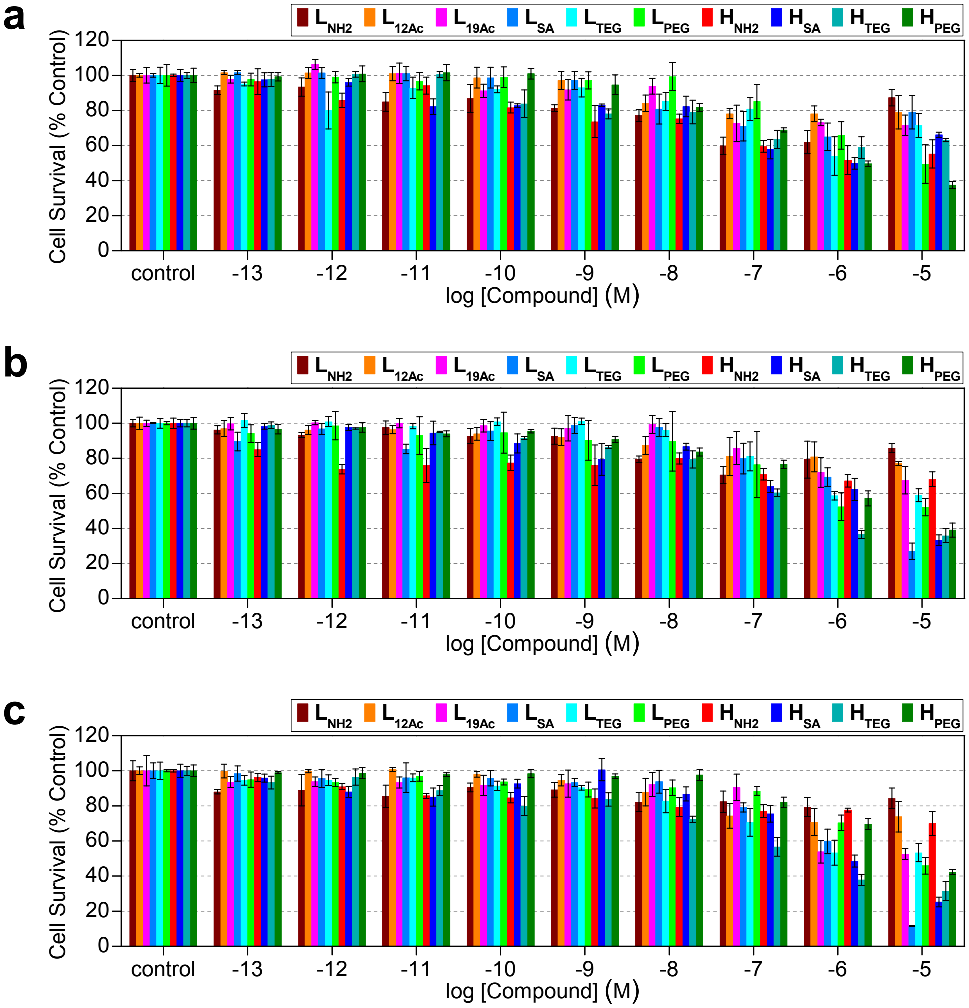

Fig. S36. Cytotoxicity assay results of targeted agents (*i.e.*, multivalent ligands) at U87MG cell cultures: (a) 24 h incubation; (b) 48 h incubation; (c) 72 h incubation. Our compounds were deemed relatively nontoxic under microdosing conditions for *in vivo* studies conducted herein (SPECT imaging and biodistribution studies). See Experimental Section for details.

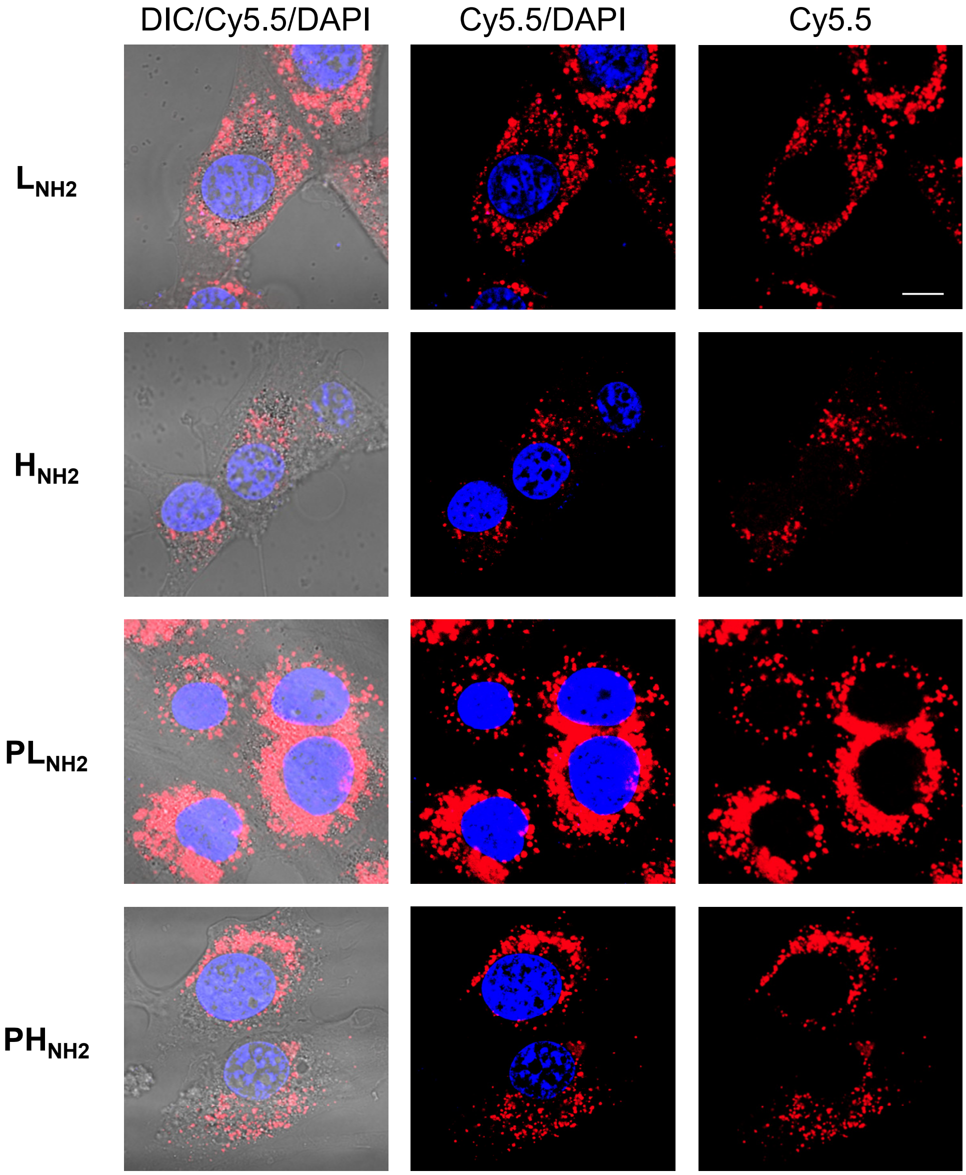

Fig. S37. Confocal laser fluorescence micrographs (1000× magnification) of U87MG cells incubated for 24 h at 37 °C with targeted or untargeted agents with amine as the IFG (1.8 μM). DIC: differential interference contrast; Cy5.5: red fluorescence; DAPI: blue fluorescence. Scale bar, 10 μm.

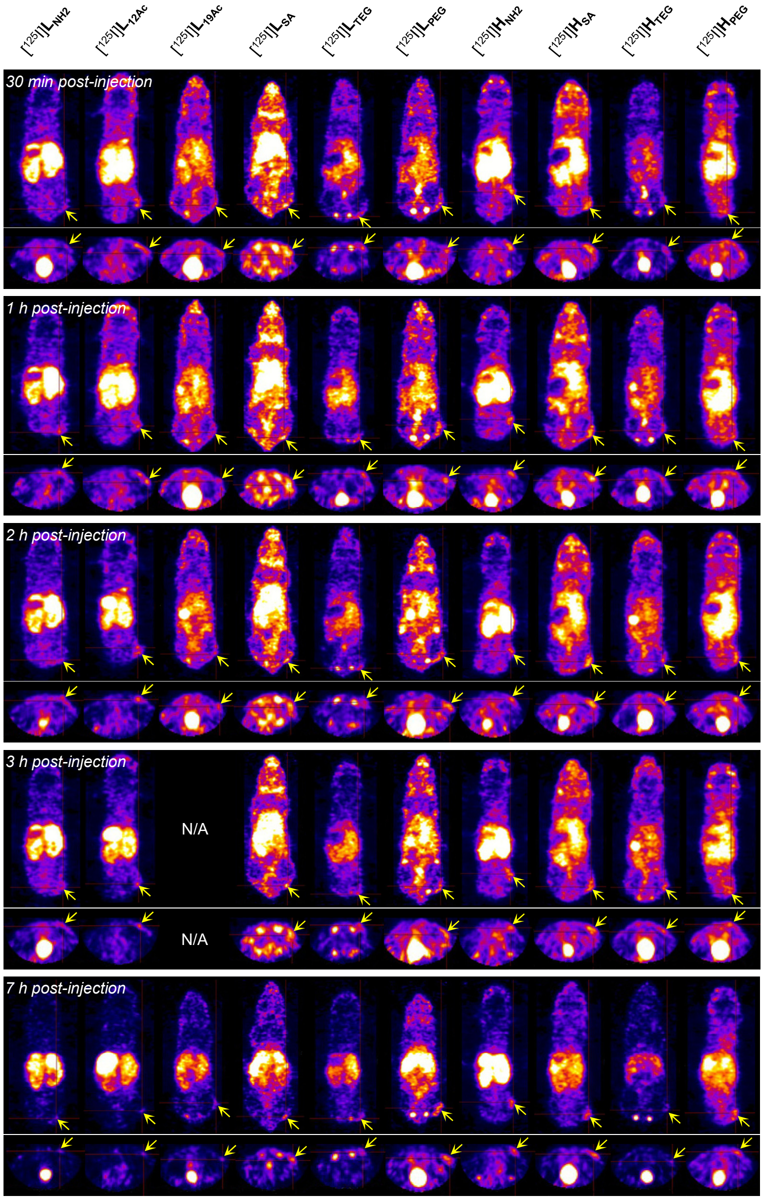

Fig. S38. SPECT images (top: coronal views; bottom: axial views) at earlier time points of mice bearing U87MG tumors injected with targeted agents (*i.e.*, multivalent ligands). These results are a part of the time-course SPECT imaging series to estimate the tumor-targeting efficiency of our nano-sized agents, which are also partially shown (2 hpi, 7 hpi, and 24 hpi) in Fig. 4a in the main text. Here, the targeted agents were injected as radiolabeled with iodine-125. See Experimental Section for details.

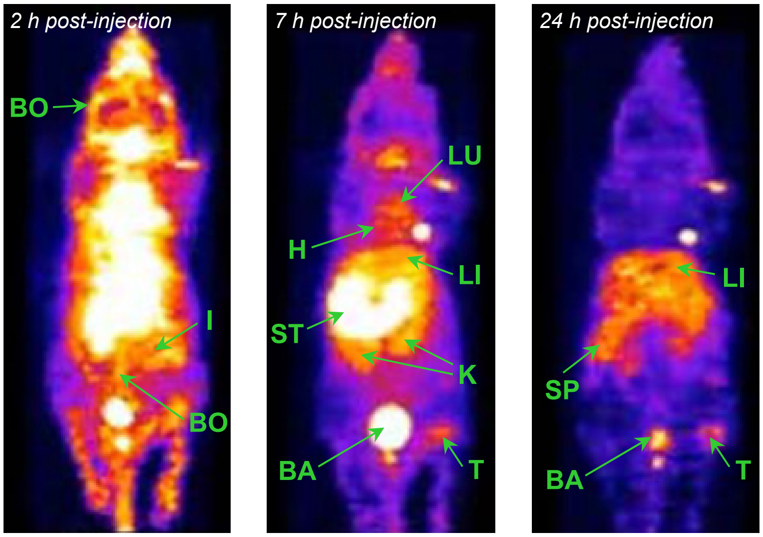

Fig. S39. Position of each organ marked in the SPECT images of a mouse injected with radiolabeled $\mathbf{H}_{\mathbf{SA}}$. Here the snapshot images taken from the video file (Movie S1) are shown. LU: lung; H: heart; LI: liver; SP: spleen; K: kidney; ST: stomach; I: intestine; BO: blood (*i.e.*, vein/artery); BA: bladder; T: tumor.

Movie S1. Time-course SPECT images of mice bearing U87MG tumors injected with targeted agents radiolabeled with iodine-125. See Experimental Section for details.

Movie S2. Time-course SPECT images of mice bearing U87MG tumors injected with untargeted agents radiolabeled with iodine-125. See Experimental Section for details.

REFERENCES

1. Y. Kim, A. M. Klutz and K. A. Jacobson, *Bioconjugate Chem.*, 2008, **19**, 1660.
2. S. You, H.-y. Jung, C. Lee, Y. H. Choe, J. Y. Heo, G.-T. Gang, S.-K. Byun, W. K. Kim, C.-H. Lee, D.-E. Kim, Y. I. Kim and Y. Kim, *J. Control. Release*, 2016, **226**, 258.
3. O. Glatter and O. Kratky, *Small angle X-ray scattering*, Academic Press, London, 1982.
4. S. Förster, L. Apostol and W. Bras, *J. Appl. Cryst.*, 2010, **43**, 639.
5. S. Förster, A. Timmann, C. Schellbach, A. Meyer, S. S. Funari, P. Mulvaney and R. Knott, *J. Phys. Chem. B*, 2005, **109**, 1347.
6. Y.-H. Kim, J. Jeon, S. H. Hong, W.-K. Rhim, Y.-S. Lee, H. Youn, J.-K. Chung, M. C. Lee, D. S. Lee, K. W. Kang and J.-M. Nam, *Small*, 2011, **7**, 2052.
7. A. P. Davenport and F. D. Russell, *Curr. Direc. Radiopharm. Res. Dev.*, 1996, 169.
8. S. Wilhelm, A. J. Tavares, Q. Dai, S. Ohta, J. Audet, H. F. Dvorak and W. C. W. Chan, *Nat. Rev. Mater.*, 2016, **1**, 16014.
